# Prognoses and genomic analyses of proteasome 26S subunit, ATPase (PSMC) family genes in clinical breast cancer

**DOI:** 10.18632/aging.203345

**Published:** 2021-07-30

**Authors:** Tzu-Jen Kao, Chung-Che Wu, Nam Nhut Phan, Yen-Hsi Liu, Hoang Dang Khoa Ta, Gangga Anuraga, Yung-Fu Wu, Kuen-Haur Lee, Jian-Ying Chuang, Chih-Yang Wang

**Affiliations:** 1 The Ph.D. Program for Neural Regenerative Medicine, Taipei Medical University, Taipei 11031, Taiwan; 2 Division of Neurosurgery, Department of Surgery, School of Medicine, College of Medicine, Taipei Medical University, Taipei 11031, Taiwan; 3 Division of Neurosurgery, Department of Surgery, Taipei Medical University Hospital, Taipei 11031, Taiwan; 4 NTT Institute of Hi-Technology, Nguyen Tat Thanh University, Ho Chi Minh 700000, Vietnam; 5 School of Chinese Medicine for Post-Baccalaureate, I-Shou University, Kaohsiung 82445, Taiwan; 6 Ph.D. Program for Cancer Molecular Biology and Drug Discovery, College of Medical Science, Taipei Medical University, Taipei 11031, Taiwan; 7 Graduate Institute of Cancer Biology and Drug Discovery, College of Medical Science and Technology, Taipei Medical University, Taipei 11031, Taiwan; 8 Department of Statistics, Faculty of Science and Technology, PGRI Adi Buana University, Surabaya, East Java 60234, Indonesia; 9 Department of Medical Research, Tri-Service General Hospital, School of Medicine, National Defense Medical Center, Taipei 11490, Taiwan; 10 Cancer Center, Wan Fang Hospital, Taipei Medical University, Taipei 11031, Taiwan; 11 TMU Research Center of Cancer Translational Medicine, Taipei Medical University, Taipei 11031, Taiwan; 12 Cell Physiology and Molecular Image Research Center, Wan Fang Hospital, Taipei Medical University, Taipei 11031, Taiwan; 13 Department of Biomedical Science and Environmental Biology, Kaohsiung Medical University, Kaohsiung 80708, Taiwan

**Keywords:** PSMC family genes, breast cancer, bioinformatics

## Abstract

Breast cancer is a complex disease, and several processes are involved in its development. Therefore, potential therapeutic targets need to be discovered for these patients. Proteasome 26S subunit, ATPase gene (PSMC) family members are well reported to be involved in protein degradation. However, their roles in breast cancer are still unknown and need to be comprehensively researched. Leveraging publicly available databases, such as cBioPortal and Oncomine, for high-throughput transcriptomic profiling to provide evidence-based targets for breast cancer is a rapid and robust approach. By integrating the aforementioned databases with the Kaplan–Meier plotter database, we investigated potential roles of six PSMC family members in breast cancer at the messenger RNA level and their correlations with patient survival. The present findings showed significantly higher expression profiles of PSMC2, PSMC3, PSMC4, PSMC5, and PSMC6 in breast cancer compared to normal breast tissues. Besides, positive correlations were also revealed between PSMC family genes and ubiquinone metabolism, cell cycle, and cytoskeletal remodeling. Meanwhile, we discovered that high levels of PSMC1, PSMC3, PSMC4, PSMC5, and PSMC6 transcripts were positively correlated with poor survival, which likely shows their importance in breast cancer development. Collectively, PSMC family members have the potential to be novel and essential prognostic biomarkers for breast cancer development.

## INTRODUCTION

In 2020, breast cancer accounted for 30% of all types of cancer in women in the United States. Expressions of the estrogen receptor (ER), progesterone receptor, and human epidermal growth factor receptor (HER)-2 are used to subgroup breast cancer cases. Currently, salvage therapy for breast cancer patients includes fulvestrant (selective ER downregulators) [1, 2], cyclin-dependent kinase 4/6 inhibitors [3], aromatase inhibitors combined with everolimus (a mammalian analog of rapamycin which acts as a mammalian target of rapamycin (mTOR) inhibitor) [4], and histone deacetylase (HDAC) inhibitors [5]. High expression of B-cell lymphoma 2 was detected in nearly 70% of metastatic breast cancer patients, and treatment with a selective inhibitor improved apoptosis in a preclinical model of breast cancer [6, 7]. Meanwhile, proteasome 26S subunit ATPase (PSMC), proteasome 20S subunit beta (PSMB), GATA-binding protein, serine/threonine kinase, and matrix metallopeptidase family genes, signal transducer and activator of transcription (STAT), Notch, and phosphatidylinositol 3-kinase (PI3K) were reported to be causes of those alterations [8–11].

The PSMC family is comprised of six members, namely PSMC1, PSMC2, PSMC3, PSMC4, PSMC5, and PSMC6, which partially constitute formation of the 19S regulatory complex. This complex plays an important role in regulating the 26S proteasome, which in turn, catalyzes the unfolding and translocation of substrates into the 20S proteasome. In addition, members of the PSMC gene family, except for PSMC3, are known to cause N-CoR degradation [12]. Previous studies showed that PSMC6 promotes osteoblast apoptosis and cancer cell proliferation, while PSMC2 inhibits apoptosis. Furthermore, PSMC6 also inhibited activation of the PI3K/AKT signaling pathway in an animal model of ovariectomy-induced osteoporosis [13–15]. PSMC5 participates in degradation of Tln1 and angiogenesis [16]. In hepatocellular carcinoma cells, knockdown of PSMC3IP resulted in suppression of xenograft proliferation and tumorigenesis [17].

High-throughput technologies are widely used as systemic approaches to explore differences in expressions of thousands of genes for both biological and genomics systems [18–20]. It is well recognized that many upregulated and downregulated genes are associated with oncogenic or tumor-suppressive functions in cancer development [21–26]. Nevertheless, a holistic approach to exploring messenger (m)RNA levels of the entire PSMC family in breast cancer has not been conducted.

Therefore, in the present study, we analyzed all available mRNA data from public breast cancer databases, comparing datasets from breast cancer patients with those from normal tissues. We also predicted interactive networks and gene regulatory networks related to the PSMC family to determine potential biomarkers. A meta-analysis approach was adopted to screen downstream molecules associated with PSMC genes. Based on our analysis, PSMC family members and their downstream-regulated genes are potential candidates for new therapeutic targets in breast cancer progression.

## RESULTS

### PSMC family members play crucial roles in breast cancer progression

**Figure 1:**
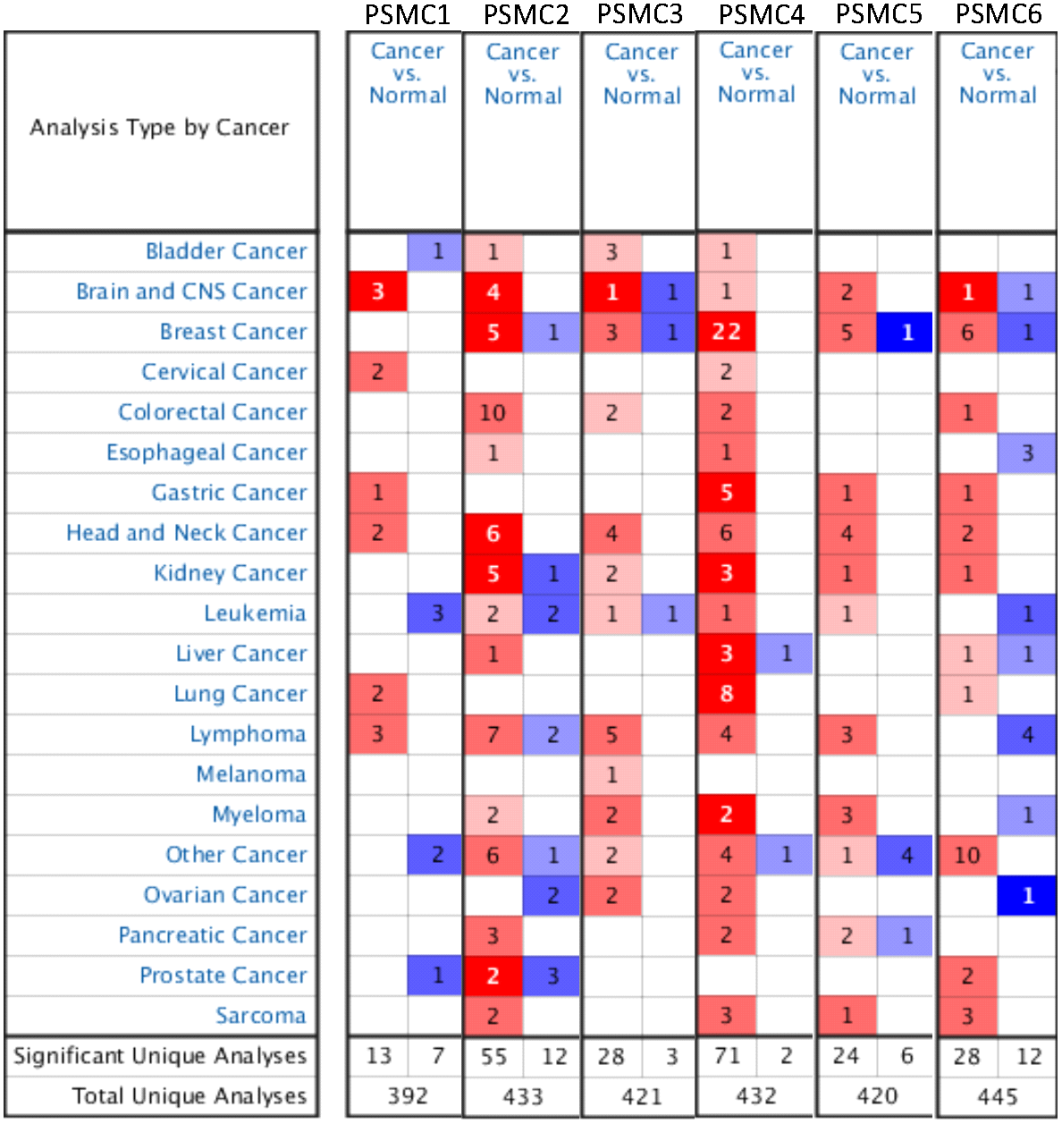
**Overview of mRNA expression levels of proteasome 26S subunit, ATPase (PSMC) genes in multiple types and subtypes of cancer from the Oncomine database.** The analysis compared expressions of target genes in breast cancer tissues relative to normal matched tissues. Red and blue gradients display the top-ranked genes in specific datasets. Significant unique analysis means the number of datasets that satisfied the threshold of >2 multiples of change, *p*<0.05, and in the top 10% gene ranking.

Previous studies identified six PSMC family members in *Homo* species, and some of these genes play crucial roles in cancer progression. Oncomine platform contained a total of 392 unique analyses for PSMC1 expression, and PSMC1 had significant in 13 of 392 unique analyses. PSMC2 had significant in 55 of 433 unique analyses, PSMC3 had significant in 28 of 421 unique analyses, PSMC4 had significant in 71 of 432 unique analyses, PSMC5 had significant in 24 of 420 unique analyses, PSMC6 had significant in 28 of 445 unique analyses (Figure 1). However, a meta-analysis is needed to clarify gene signatures of PSMC family members in breast cancer. According to our results from an Oncomine analysis of mRNA expressions of PSMC2, PSMC3, PSMC4, PSMC5, and PSMC6, these members are highly upregulated in breast cancer tissues; therefore, we chose breast cancer to perform further bioinformatics analyses (Figure 1). Furthermore, in the METABRIC database, expressions of PSMC members in breast cancer tissues were significantly higher than those in normal tissues; *p* values ranged from 2.16E-45 to 0.023 for PSMC1, 1.37E-29 to 0.016 for PSMC2, 3.18E-21 to 0.001 for PSMC3, 1.28E-53 to 0.018 for PSMC4, 7.02E-36 to 0.041 for PSMC5, and 9.03E-12 to 0.039 for PSMC6 (Supplementary Table 1). Meanwhile, to further explore gene expressions of the entire PSMC family in breast cancer, we compared transcript levels of different breast cancer subtypes, such as the triple-negative, HER-2, and luminal subtypes, relative to normal breast tissues, in TCGA database (Supplementary Figure 1). Interestingly, we discovered that PSMC genes were overexpressed in a subtype-specific manner: specifically, PSMC1, PSMC2, PSMC3, PSMC4 were highly expressed in the triple-negative subtype, PSMC5 in HER-2, and PSMC6 in luminal cancer. These results suggest oncogenic effects of PSMC family genes on tumor progression.

### Associations between mRNA levels of the PSMC family and clinicopathological parameters in breast cancer patients and cell lines

**Figure 2:**
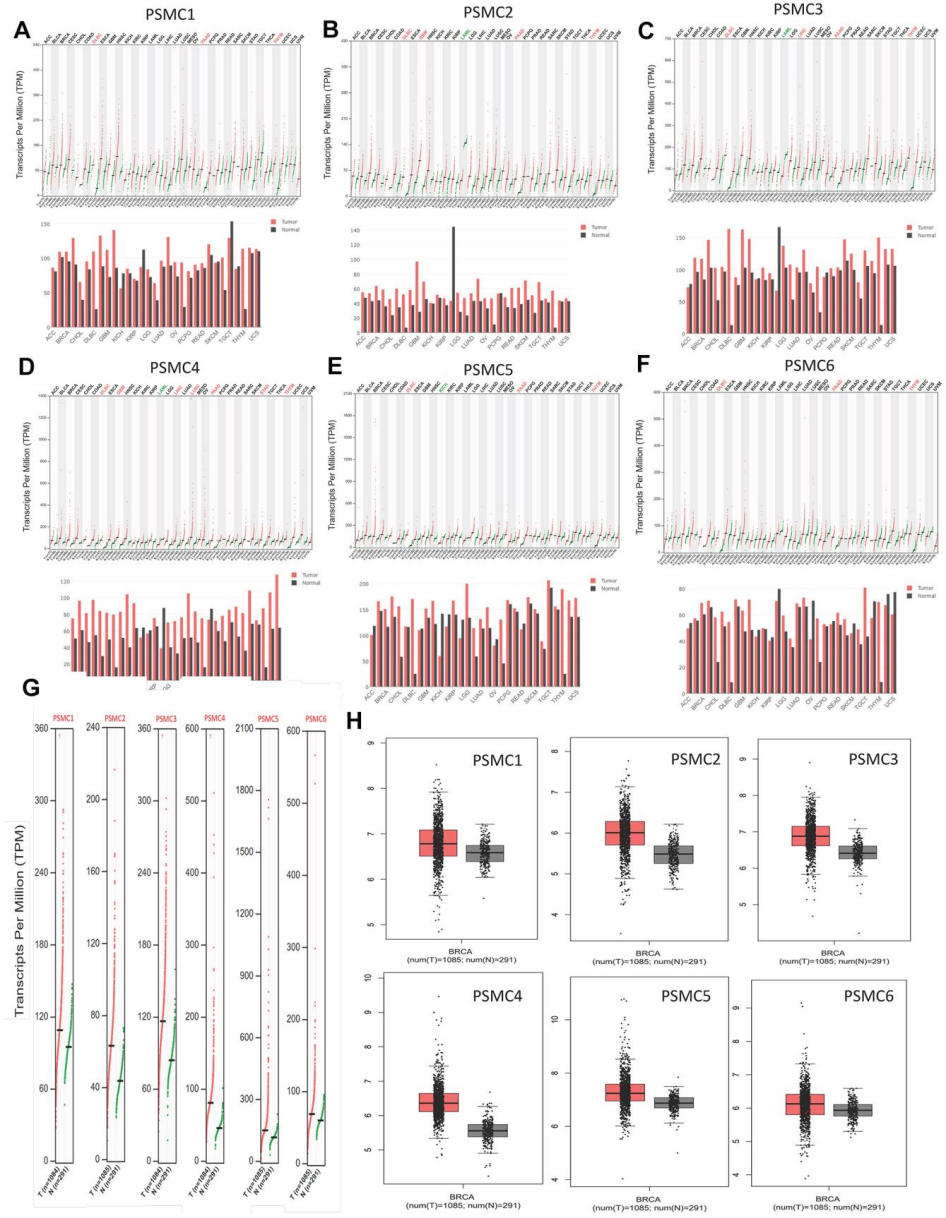
**Transcript expressions of proteasome 26S subunit, ATPase (PSMC) genes in breast cancer.** (**A**–**F**) Expressions of PSMC members in multiple types of cancer. (**G**, **H**) Transcript expressions of PSMC members in clinical breast cancer patients. Red bar and box plots show tumor expression while green/gray colors represent normal breast tissues.

**Figure 3:**
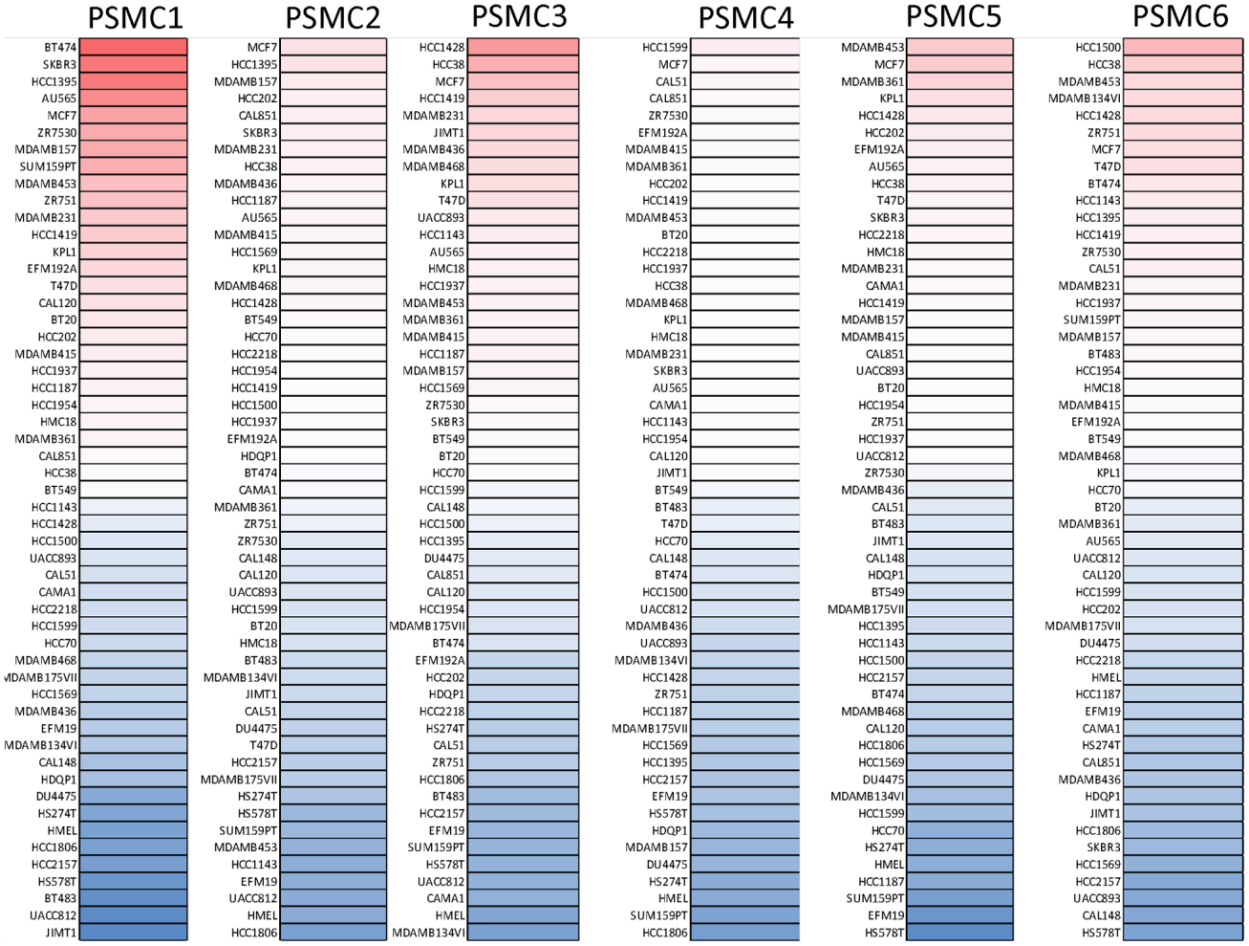
**Expressions of proteasome 26S subunit, ATPase (PSMC) genes in different breast cancer cell lines.** Heatmap plots were acquired from the CCLE database, which indicated expression levels of six PSMC members in breast cancer cell lines. The upper blocks in red indicate over-expression, whereas the bottom blocks indicate under-expression.

GEPIA2 datasets were used to analyze mRNA levels of PSMC members in breast cancer tissues compared to normal tissues. We found that levels of all six PSMCs were upregulated in breast cancer tissues relative to normal breast tissues (Figure 2A–2H). Additionally, analysis of the CCLE dataset (https://www.broadinstitute.org/ccle) also showed differential expressions of PSMC family members in breast cell lines (Figure 3).

### Genes coexpressed with PSMC family genes in breast cancer

**Figure 4:**
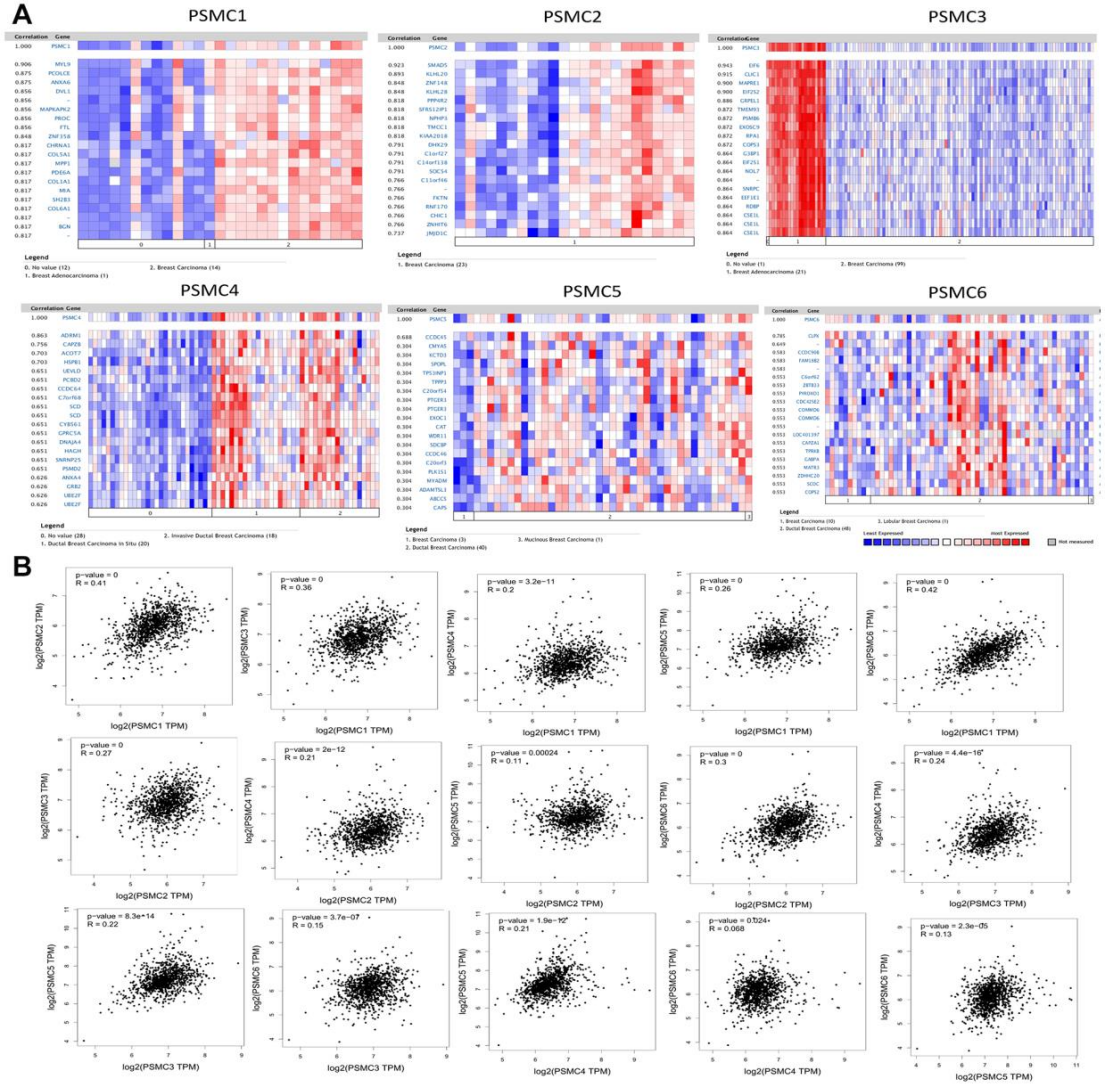
**Genes coexpressed with the proteasome 26S subunit, ATPase (PSMC) family and correlations among the six PSMC genes in breast cancer.** (**A**) Genes coexpressed with PSMC genes in breast cancer from the Oncomine platform. (**B**) Correlations among PSMC genes in breast cancer from the GEPIA2 platform.

We analyzed genes coexpressed with PSMC1 in the Perou Breast 2 dataset from the Oncomine platform. We found that PSMC1 was positively correlated with *MYL9*, *PCOLCE*, *ANXA6*, *DVL1*, *MAPKAPK2*, *PROC*, *FTL*, *ZNF358*, *CHRNA1*, *COL5A1*, *MPP1*, *PDE6A*, *COL1A1*, *MIA*, *SH2B3*, *COL6A1*, and *BGN*. We used the Landemaine dataset to analyze genes coexpressed with PSMC2 and found that its expression was positively correlated with *SMAD5*, *KLHL20*, *ZNF148*, *KLHL28*, *PPP4R2*, *SFRS12IP1*, *NPHP3*, *TMCC1*, *KIAA2018*, *DHX29*, *Clorf27*, *C14orf138*, *SOCS4*, *Cllorf46*, *FKTN*, *RNF170*, *CHIC1*, *ZNHIT6*, and *JMIDIC*. We analyzed genes coexpressed with PSMC3 in the Minn dataset and found that its expression was positively correlated with *EIF6*, *CLIC1*, *MAPRE1*, *EIF2S2*, *GRPEL1*, *TMEM93*, *PSMB6*, *EXOSC9*, *RPAI*, *COPS3*, *G3BP1*, *EIF2S1*, *NOL7*, *SNRPC*, *EEFIEl*, *RDBP*, and *CSEIL*. As for the genes coexpressed with PSMC4, we used the Ma dataset and found that it was positively correlated with expressions of *ADRM1*, *CAPZB*, *ACOT7*, *HSPB1*, *UEVLD*, *PCBD2*, *CCDC64*, *C7orf68*, *SCD*, *CYB561*, *GPRC5A*, *DNAJA4*, *HAGH*, *SNRNP25*, *PSMD2*, *ANXA4*, *GRB2*, *UBE2F*, and *UBEZF*. We analyzed genes coexpressed with PSMC5 in the Julka dataset and found that its expression was positively correlated with *CCDC45*, *CMYA5*, *KCTD3*, *SPOPL*, *TP53INP1*, *TPPP3*, *C20orf54*, *PTGER3*, *EXOC1*, *CAT*, *WDR11*, *SDCBP*, *CCDC46*, *C20orf3*, *PLK1S1*, *MYADM*, *ADAMTSL3*, *ABCC5*, and *CAPS*. For genes coexpressed with PSMC6, we used the Kreike dataset and found that its expression was positively correlated with *CLPX*, *CCDC9OB*, *FAM18B2*, *C60rf62*, *ZBTB33*, *PYROXD1*, *CDC42SE2*, *COMMD6*, *LOC401397*, *CAPZAI*, *TPRKB*, *GABPA*, *MATR3*, *ZDHHC20*, *SCOC*, and *COPS2* (Figure 4A).

Additionally, associations among PSMC1, PSMC2, PSMC3, PSMC4, PSMC5, and PSMC6 were also analyzed using the GEPIA dataset. Specifically, PSMC1 was positively correlated with PSMC2 (*R*=0.41, *p*<0.05), PSMC3 (*R*=0.36, *p*<0.05), PSMC4 (*R*=0.2, *p*<0.05), PSMC5 (*R*=0.26, *p*<0.05), and PSMC6 (*R*=0.42, *p*<0.05). PSMC2 was positively correlated with PSMC3 (*R*=0.27, *p*<0.05), PSMC4 (*R*=0.21, *p*<0.05), PSMC5 (*R*=0.11, *p*<0.05), and PSMC6 (*R*=0.3, *p*<0.05). PSMC3 was positively correlated with PSMC4 (*R*=0.24, *p*<0.05), PSMC5 (*R*=0.22, *p*<0.05), and PSMC6 (*R*=0.15, *p*<0.05). PSMC4 was positively correlated with PSMC5 (*R*=0.021, *p*<0.05) and PSMC6 (*R*=0.068, *p*<0.05). Finally, PSMC5 was positively correlated with PSMC6 (*R*=0.13, *p*<0.05) (Figure 4B). Meanwhile, we obtained similar results from the cBioPortal and the Cytoscaped and METABRIC databases, which revealed that the six PSMC members were correlated with cell cycle-related genes (Supplementary Figure 2). In addition, expressions of PSMC family members were also correlated with immune infiltration profiles in breast cancer, as analyzed with the Tumor Immune Estimation Resource (TIMER; cistrome.shinyapps.io/timer) tool. Expression of each PSMC gene was associated with tumor purity and markers of different types of immune cells (Supplementary Figure 3).

### Protein expressions and prognostic values of the PSMC family in breast cancer specimens

**Figure 5:**
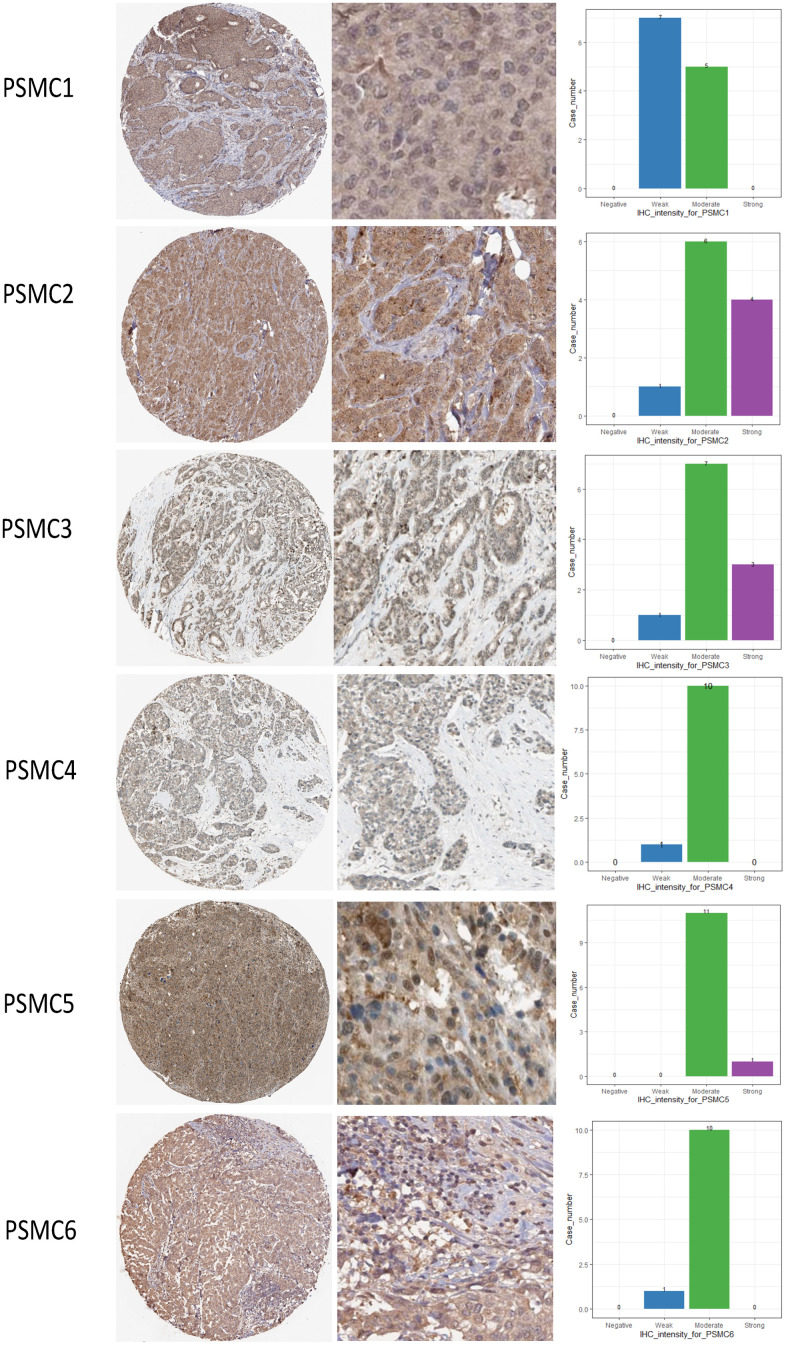
**Protein expression levels of proteasome 26S subunit, ATPase (PSMC) family members across clinical specimens of breast cancer.** PSMC1, PSMC4, and PSMC6 proteins were moderately expressed, and some clinical tissues showed strong PSMC2, PSMC3, and PSMC5 protein expressions in breast cancer.

**Figure 6:**
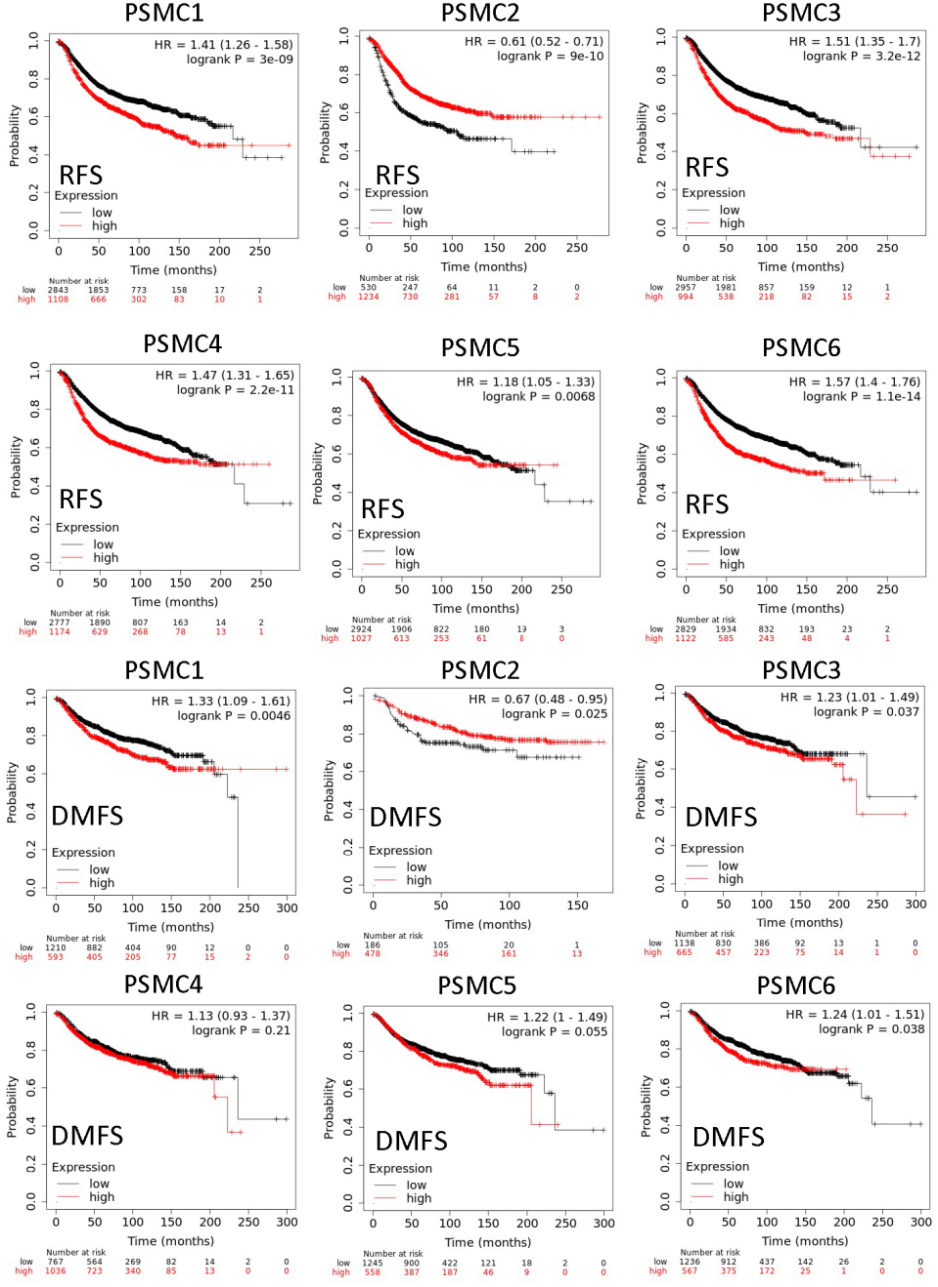
**Relationship between expressions of proteasome 26S subunit, ATPase (PSMC) family members with recurrence-free survival (RFS) and distant metastasis-free survival (DMFS) from clinical breast cancer patients (*n*=2898).** Kaplan–Meier plots show correlations of RFS and DMFS in breast cancer patients with high and low expression levels of PSMC family members using the median of expression as the cutoff. Red and black lines respectively represent higher and lower values than the median. High expression levels of most PSMC members were associated with poor survival, whereas high expression levels of PSMC2 were associated with significantly better survival rates (*p*<0.05).

Since PSMC family genes were differentially expressed in samples from breast cancer patients, we next explored the potential roles of these genes in human breast cancer tissues, correlating their expressions with other potential biomarkers related to molecular subtypes of breast cancer. To determine expressions of PSMC family members and their clinical relevance, we analyzed protein expressions of individual PSMC members in clinical specimens from the Human Protein Atlas. The data demonstrated that PSMC1-6 presented moderate protein expressions, and PSMC2, PSMC3, and PSMC5 were highly expressed in certain clinical tissues from breast cancer specimens (Figure 5). The Kaplan–Meier plotter database also showed that PSMC1, PSMC3, PSMC4, PSMC5, and PSMC6 had high expression levels in breast cancer tissues may have oncogenic roles in breast cancer progression. High transcription levels of PSMC1, PSMC3, PSMC4, PSMC5, and PSMC6 predicted poor survival, whereas PSMC2 did not show the same pattern (Figure 6).

### Pathway and network analyses of PSMC family member genes

**Figure 7:**
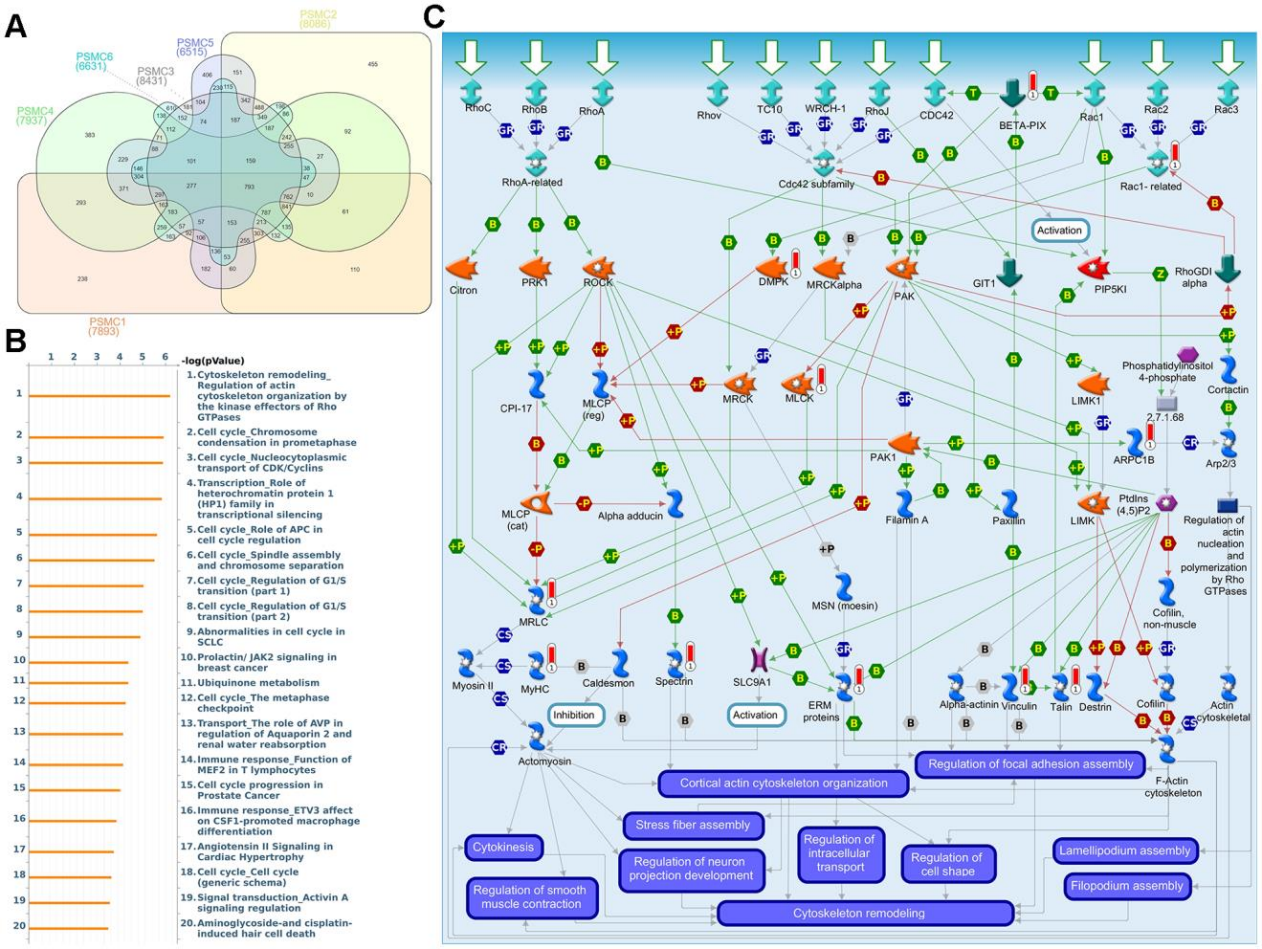
**Coexpression of proteasome 26S subunit, ATPase (PSMC) genes and signal transduction pathways in breast cancer tissues.** (**A**) Venn diagram of PSMC family coexpression networks in METABRIC breast cancer databases. PSMC genes were analyzed using METABRIC databases, and the intersection of coexpressed genes was plotted. (**B**) To explore potential networks regulated by PSMC family genes, we exported coexpressed genes and further uploaded them to the MetaCore platform for a pathway analysis. (**C**) The MetaCore pathway analysis of "biological processes" indicated that “Cytoskeleton remodeling_Regulation of actin cytoskeleton organization by the kinase effectors of Rho GTPases"-related pathways were correlated with breast cancer development.

**Figure 8:**
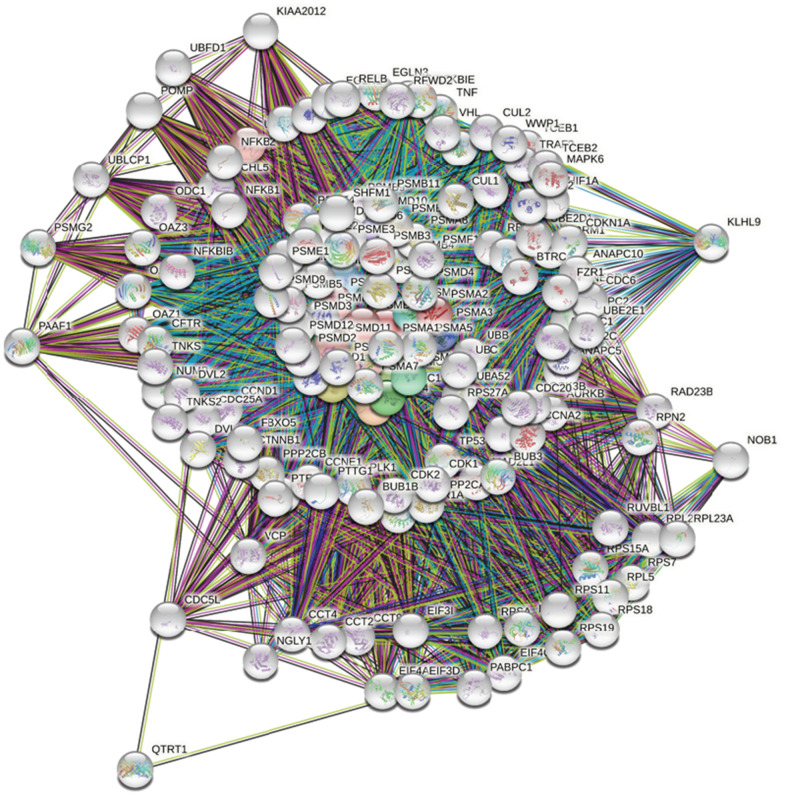
**Network analysis of protein-protein interactions (PPIs) by the STRING platform.** Genes associated with the proteasome 26S subunit, ATPase (PSMC) family were uploaded to the STRING platform to establish the network. Using k-means clustering, the network was further separated into different clusters.

First, to explore the universally regulated pathways of the entire PSMC family, GeneGo Metacore was leveraged to investigate downstream networks according to coexpression patterns of PSMC genes. An analysis on the GeneGo Metacore platform demonstrated that genes coexpressed with the six PSMC genes participated in biological processes related to cancer progression. MetaCore can be used to construct downstream networks associated with biological processes from uploaded genes. By uploading PSMC coexpressed genes from the METABRIC database into the Metacore platform, we found that several cancer progression-related pathways were correlated with genes of the PSMC family (Supplementary Figures 4, 5 and Supplementary Table 2), including "Cytoskeleton remodeling_Regulation of actin cytoskeleton organization by the kinase effectors of Rho GTPases", "Cell cycle_Role of APC in cell cycle regulation", "Cell cycle_Chromosome condensation in prometaphase", "Cell cycle_Nucleocytoplasmic transport of CDK/Cyclins", and "Transcription_Role of heterochromatin protein 1 family in transcriptional silencing" (Figure 7). Next, the STRING platform was used to externally validate and search for potential protein-protein interactions (PPIs). The resulting network with a core cluster contained all of the genes associated with cancer progression and metastasis (Figure 8).

Next, we explored whether individual genes of the PSMC family regulate specific pathways and networks in breast cancer development. We obtained coexpression profiles for PSMC1 from TCGA and METABRIC breast cancer datasets. Afterward, GeneGo Metacore annotations of each biological process suggested that genes coexpressed with PSMC1 were involved inG-protein-coupled receptor (GPCR)- and apoptosis-related pathways and networks such as “Chemotaxis_Lysophosphatidic acid signaling via GPCRs”, “Development_Positive regulation of WNT/Beta-catenin signaling in the cytoplasm”, and “Apoptosis and survival_Regulation of apoptosis by mitochondrial proteins”, and play essential roles in breast cancer (Supplementary Figure 6 and Supplementary Table 3). PSMC2-related genes were involved in Wnt- and hypoxia-related pathways and networks such as “Development_Negative regulation of WNT/Beta-catenin signaling in the cytoplasm” and “Transcription_HIF-1 targets”, which may be involved in breast cancer (Supplementary Figure 7 and Supplementary Table 4). Genes coexpressed with PSMC3 participated in processes of cytoskeleton- and organization-related pathways and networks such as “Cytoskeleton remodeling_Regulation of actin cytoskeleton organization by the kinase effectors of Rho GTPases” (Supplementary Figure 8 and Supplementary Table 5).

PSMC4-related genes were involved in mitogen-activated protein kinase (MAPK)- and inflammation-related pathways and networks such as “Signal transduction_CXCR4 signaling via MAPKs cascades” and “Signal transduction_Angiotensin II/AGTR1 signaling via Notch, Beta-catenin and NF-κB pathways”, which may participate in breast cancer (Supplementary Figure 9 and Supplementary Table 6). Genes found to be coexpressed with PSMC5 were involved in oxidative stress- and cell adhesion-related pathways and networks such as “Oxidative stress_ROS-induced cellular signaling” and “Cell adhesion_Tight junctions” (Supplementary Figure 10 and Supplementary Table 7). PSMC6-coexpressed genes were involved in calcium- and hormone-related pathways and networks such as “Signal transduction_Calcium-mediated signaling” and “Reproduction_Gonadotropin-releasing hormone (GnRH) signaling ”, which could participate in breast cancer (Supplementary Figure 11 and Supplementary Table 8).

## DISCUSSION

Breast cancer has the highest prevalence rate compared to other types of cancer, particularly in females. Despite several years of extraordinary efforts to increase our knowledge of tumor biology and improve surgical treatments and chemotherapies, prognoses of advanced breast cancer patients have not improved [27–32]. Therefore, it is very important to investigate new diagnostic tools and novel biomarkers that can allow us to refine patient prognoses and investigate effective interventions.

Most genes of the PSMC family are upregulated in many types and subtypes of cancer. PSMC members were proven to be involved in tumor progression. For example, overexpression of PFN1 is associated with PSMC1 in the MDA-MB-231 triple-negative breast cancer cell line and may involve multiple mechanisms for cancer progression [33]. PSMC2 is highly expressed in pancreatic cancer, and PSMC2-knockdown significantly decreased cell proliferation. PSMC3 was identified as a crucial node in a PPI network in glioma cells [34]. PSMC4 and PSMC5 contribute to prostate tumorigenesis [35]. Additionally, PSMC4 was identified as one of the best biomarkers for endometrial cancer [36], and risk model construction revealed that it is also a prognostic marker for the same type of cancer [37]. PSMC5 acts as a novel regulator and is involved in the extracellular signal-regulated kinase 1/2 signaling pathway [38], and it was observed to have a relatively higher cytoplasmic expression pattern in most cancer types [39]. Both the PSMC6 and MAPK8 genes were upregulated in melanosis coli patients [40], and CRISPR Genome-Wide Screening demonstrated that the PSMC6 subunit is an important and sensitive target for bortezomib in multiple myeloma cells [41].

Since the roles of PSMC family members in breast cancer are poorly described, the present findings show their importance, by providing preliminary clues for prospective studies in breast cancer research. Findings from the current study are in line with previous reports on the roles of PSMC genes in cancer. Both mRNA and protein levels of PSMC2, PSMC3, PSMC4, PSMC5, and PSMC6 were significant in cancer tissues, and PSMC1, PSMC3, PSMC4, PSMC5, and PSMC6 overexpression was associated with poor prognoses of breast cancer patients. Meanwhile, to further clarify its role of PSMC family genes in pan-cancer, we also used GEPIA2 database to confirm that PSMC family genes had prognostic value in these integrated analyses (Supplementary Figure 12), as well as the PSMC family gene expressions in CCLE database (Supplementary Figure 13), The coexpression analysis revealed the positive correlative roles of PSMC family genes in cytoskeletal remodeling and CDK/cyclins, as well as cell cycle-related pathways and networks, which are consistent with previous studies. To some extent, this is the first report on both mRNA and protein expressions of the PSMC family in cancer cell lines and tissues, together with their associations with breast cancer patient survival.

Collectively, by integrating multiple high-throughput databases, our study uncovered that PSMC genes have prognostic and predictive value in breast cancer. To comprehensively provide a complete picture of the PSMC members not only in breast but also in other types and subtypes of cancer, our results can be used as hints for further examination of this family, and possibly they can serve as novel biomarkers and potential prognostic indicators in breast cancer.

## MATERIALS AND METHODS

### Oncomine analysis

Oncomine (https://www.oncomine.org/), a well-known high-throughput database for mRNA, was used to query expressions of PSMC family members [42]. In brief, each gene symbol of the PSMC family was used to search for expression levels in 20 types of cancer relative to matched normal-type samples. The search thresholds included a multiple of change of 2-fold, a *p* value of <0.01, and a gene ranking in the top 10%. The search output displayed the number of datasets that satisfied the above thresholds among all unique analyses. Upregulated genes in the generated dataset were displayed in a red gradient, decreasing with the top-ranked percentage, while downregulated genes in the generated dataset were presented in a blue gradient that decreased with the gene ranking. A gene could be upregulated in one dataset and downregulated in another depending on the search thresholds and study parameters. It is useful to examine discrepancies in gene expressions among studies, and plots of breast cancer subtypes were conducted with the ggpubr package in R environment as we previously described [43–46].

### Cancer cell line encyclopedia (CCLE) analysis

In addition to investigating mRNA expressions of PSMC family members in cancer tissues from the Oncomine database, we further searched for their expression levels in cell lines via the CCLE database (https://portals.broadinstitute.org/ccle) [47]. The CCLE is a high-throughput web-based tool with large numbers of human cancer cell lines (*n* = 1457) and unique datasets (*n* = 136,488). An RNA sequencing method was selected to search for expressions of PSMC family members in 60 breast cancer cell lines, and results were plotted with default settings as we previously described [48–50].

### Functional enrichment analysis of PSMC family members

To acquire coexpression patterns of PSMC family members in the METABRIC and cBioPortal databases [51], a Venn diagram was created using InteractiVenn (http://www.interactivenn.net/). Then, 1588 coexpressed genes were further uploaded to Gene Ontology for pathway and network analyses using the MetaCore platform (https://portal.genego.com/), a functional annotation platform for exploring the biological significance behind a large list of genes. Statistical significance as the boundary criterion was set to *p*<0.05, as we previously described [52–54].

### Search tool for the retrieval of interacting genes (STRING)

Together with investigating mRNA expression levels, we concomitantly performed searches for PPI networks of PSMC family members based on coexpressed genes using the STRING database. STRING has protein data comprising 24.6 million proteins in more than 5000 organisms, resulting in more than 2 billion interactions [55]. We selected the k-means clustering algorithm to classify target proteins into different clusters.

### Kaplan–Meier plot of survival analysis

To understand how mRNA expression levels of PSMC gene family members affected relapse-free survival (RFS) and distant metastasis-free survival (DMFS) of breast cancer patients, we performed a survival analysis using the Kaplan–Meier plotter database (https://kmplot.com/) [56]. Meanwhile, we also assessed the prognostic value of PSMC gene family members for pan-cancer analysis in GEPIA2, which contains RNA sequencing expression data from different types of tumors as well as the normal samples from the TCGA and GTEx projects, including Cholangio carcinoma, Colon adenocarcinoma, Lymphoid Neoplasm Diffuse Large B-cell Lymphoma, Esophageal carcinoma, Pancreatic adenocarcinoma, Pheochromocytoma and Paraganglioma, Prostate adenocarcinoma, Rectum adenocarcinoma, Sarcoma, Skin Cutaneous Melanoma, Stomach adenocarcinoma, Testicular Germ Cell Tumors, Thyroid carcinoma, Thymoma, Uterine Corpus Endometrial Carcinoma, Uterine Carcinosarcoma and Uveal Melanoma, Glioblastoma multiforme, Head and Neck squamous cell carcinoma, Kidney Chromophobe, Kidney renal clear cell carcinoma, Kidney renal papillary cell carcinoma, Acute Myeloid Leukemia, Brain Lower Grade Glioma, Adrenocortical carcinoma, Bladder Urothelial Carcinoma, Breast invasive carcinoma, Cervical squamous cell carcinoma and endocervical adenocarcinoma, Liver hepatocellular carcinoma, Lung adenocarcinoma, Lung squamous cell carcinoma, Mesothelioma, Ovarian serous cystadenocarcinoma [57, 58]. All default settings inKaplan–Meier were selected for our analysis, namely survival curves, log-rank p values, and hazard ratios with 95% confidence intervals (CIs).

### Tumor immune estimation resource (TIMER)

To further analyze the infiltration level of immune cells, we applied TIMER 2.0 (http://timer.comp-genomics.org) across 31 cancer types comprising of more 10,000 samples [59, 60]. The differences between normal and tumor in mRNA expression of PSMC genes were obtained using DiffExp module. We then selected B cells, T cells clusters including CD4+ and CD8+ together with neutrophils, macrophages, and dendritic cells for our analysis.

## Supplementary Material

Supplementary Figures

Supplementary Tables 1 and 2

Supplementary Table 3

Supplementary Table 4

Supplementary Table 5

Supplementary Table 6

Supplementary Table 7

Supplementary Table 8
