# Supplementary material for: Prognoses and genomic analyses of proteasome 26S subunit, ATPase (PSMC) family genes in clinical breast cancer
Source: Aging (Albany NY). 2021 Jul 30;13(14):17970. doi: 10.18632/aging.203345 (PMC8351721; doi:10.18632/aging.203345)
Supplement: Supplementary Figures [file aging-13-203345-s001.pdf]

SUPPLEMENTARY FIGURES

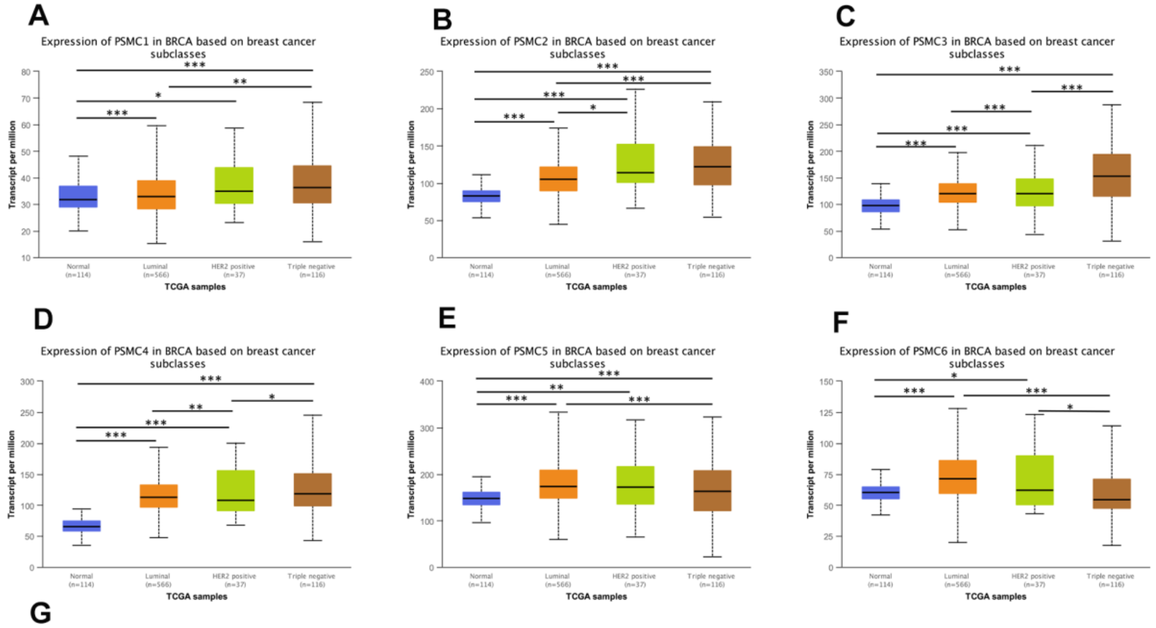

G

Comparison and P value	PSMC1	PSMC2	PSMC3	PSMC4	PSMC5	PSMC6
Normal-vs-Luminal	1.99E-05	<1E-12	<1E-12	<1E-12	2.22E-16	1.62E-12
Normal-vs-HER2 Positive	1.62E-02	9.69E-08	1.24E-04	8.21E-11	4.66E-03	1.46E-02
Normal-vs-TNBC	7.73E-07	<1E-12	1.62E-12	1.11E-16	9.96E-05	2.73E-01
Luminal-vs-HER2 Positive	1.95E-01	1.61E-02	8.59E-01	9.26E-01	6.73E-01	5.60E-01
Luminal-vs-TNBC	3.20E-03	3.14E-07	3.58E-07	1.42E-03	7.57E-07	1.27E-06
HER2 Positive-vs-TNBC	7.04E-01	4.80E-01	3.99E-04	1.05E-02	5.19E-02	4.78E-02

Supplementary Figure 1. Expressions of proteasome 26S subunit, ATPase (PSMC) family members in breast cancer in TCGA database. Comparison of members of PSMC family genes in different subtypes of breast cancer patients. (**A**) The differential expression of PSMC1 in breast cancer subclasses. (**B**) The differential expression of PSMC2 iin breast cancer subclasses. (**C**) The differential expression of PSMC3 in breast cancer subclasses (**D**) The differential expression of PSMC4 in breast cancer subclasses. (**E**) The differential expression of PSMC5 in breast cancer subclasses. (**F**) The differential expression of PSMC6 in breast cancer subclasses. (**G**) Comparison of PSMC genes between normal and breast cancer subtypes as well as within subtypes.

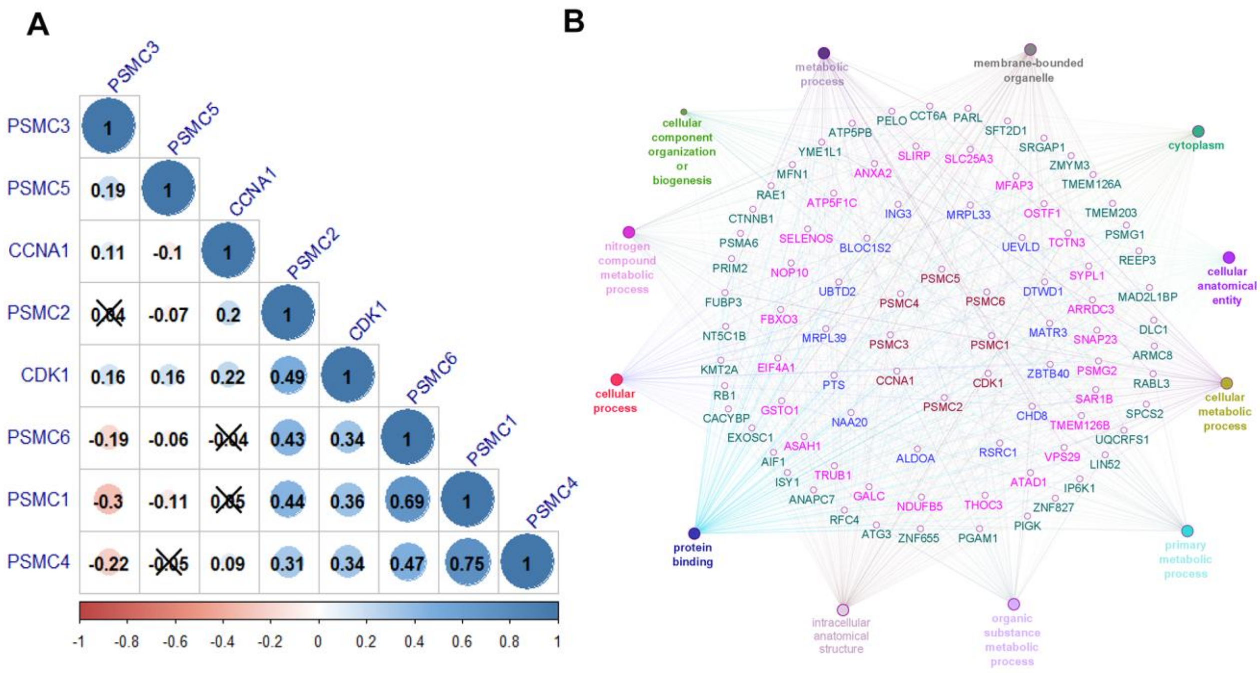

Supplementary Figure 2. Correlations among different proteasome 26S subunit, ATPase (PSMC) family members in breast cancer. (**A**) Correlations between PSMC family members and cell cycle-related genes in breast cancer patients from the METABRIC database, and insignificant correlations are marked by crosses. (**B**) Through a cytoscape analysis, high correlations between PSMC members and cancer development-related pathways were observed.

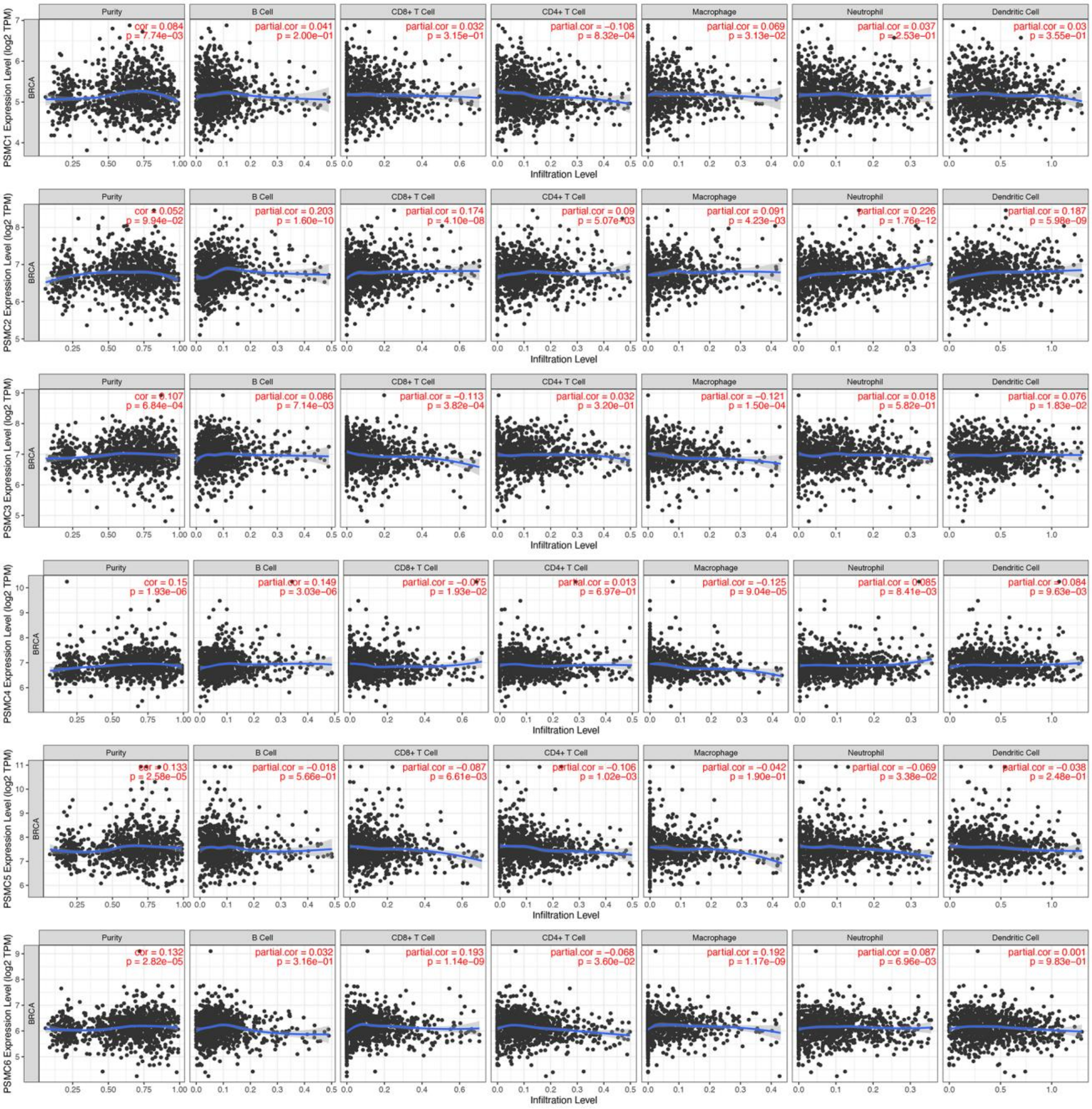

Supplementary Figure 3. Correlations between expressions of proteasome 26S subunit, ATPase (PSMC) family members and immune infiltration profiles in breast cancer. The figure shows the expression of each gene associated with tumor purity and several tumor-infiltrating immune cell markers, such as B cell, CD8+ T cell, CD4 + T cell, macrophage, neutrophil, and dendritic cell markers ($p<0.05$).

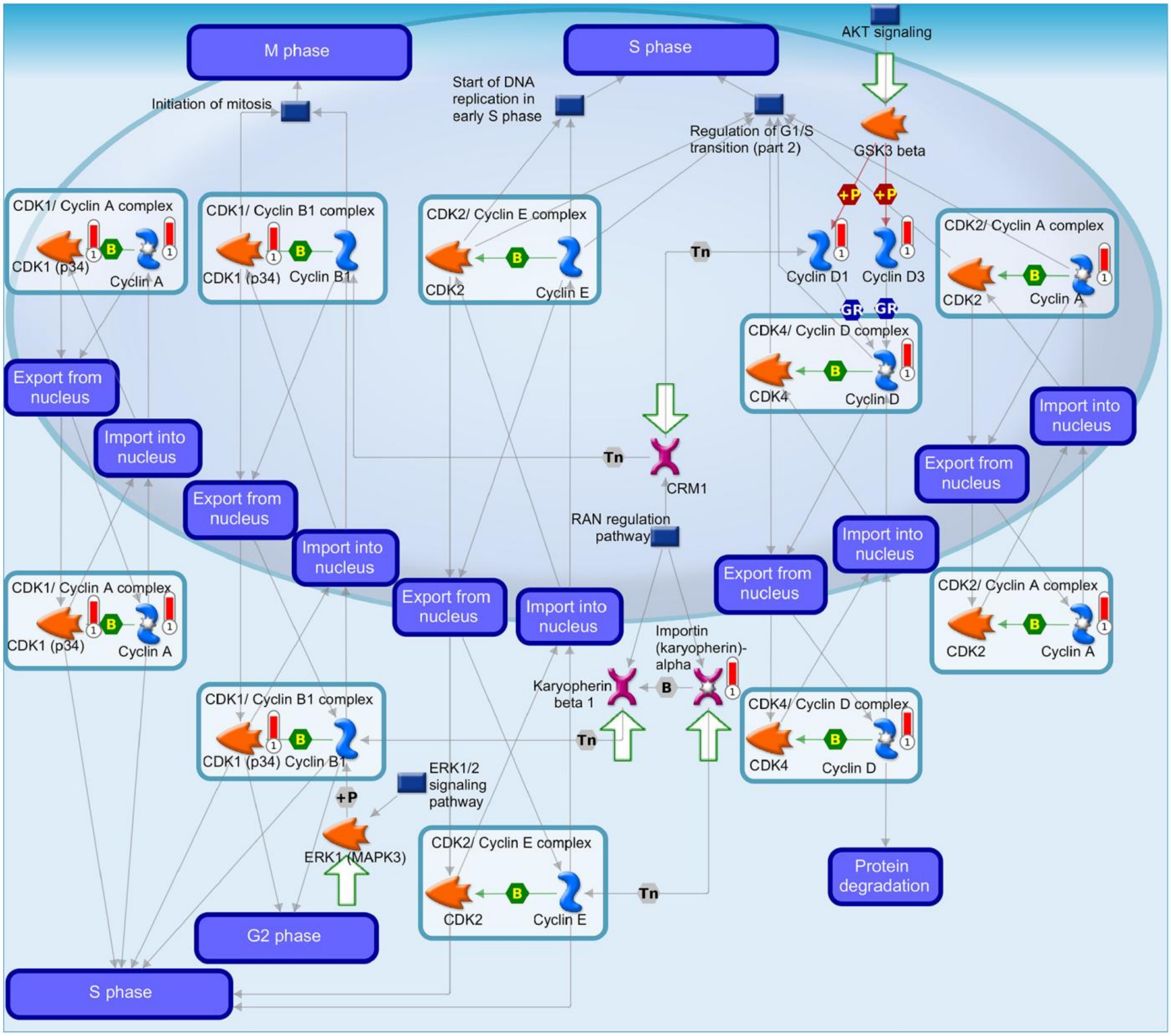

Supplementary Figure 4. Cell cycle-related networks were correlated with proteasome 26S subunit, ATPase (PSMC) family genes in breast cancer. The MetaCore pathway analysis of "biological processes" indicated that "Cell cycle_Nucleocytoplasmic transport of CDK and Cyclins"-related pathways were correlated with PSMC family genes in breast cancer development.

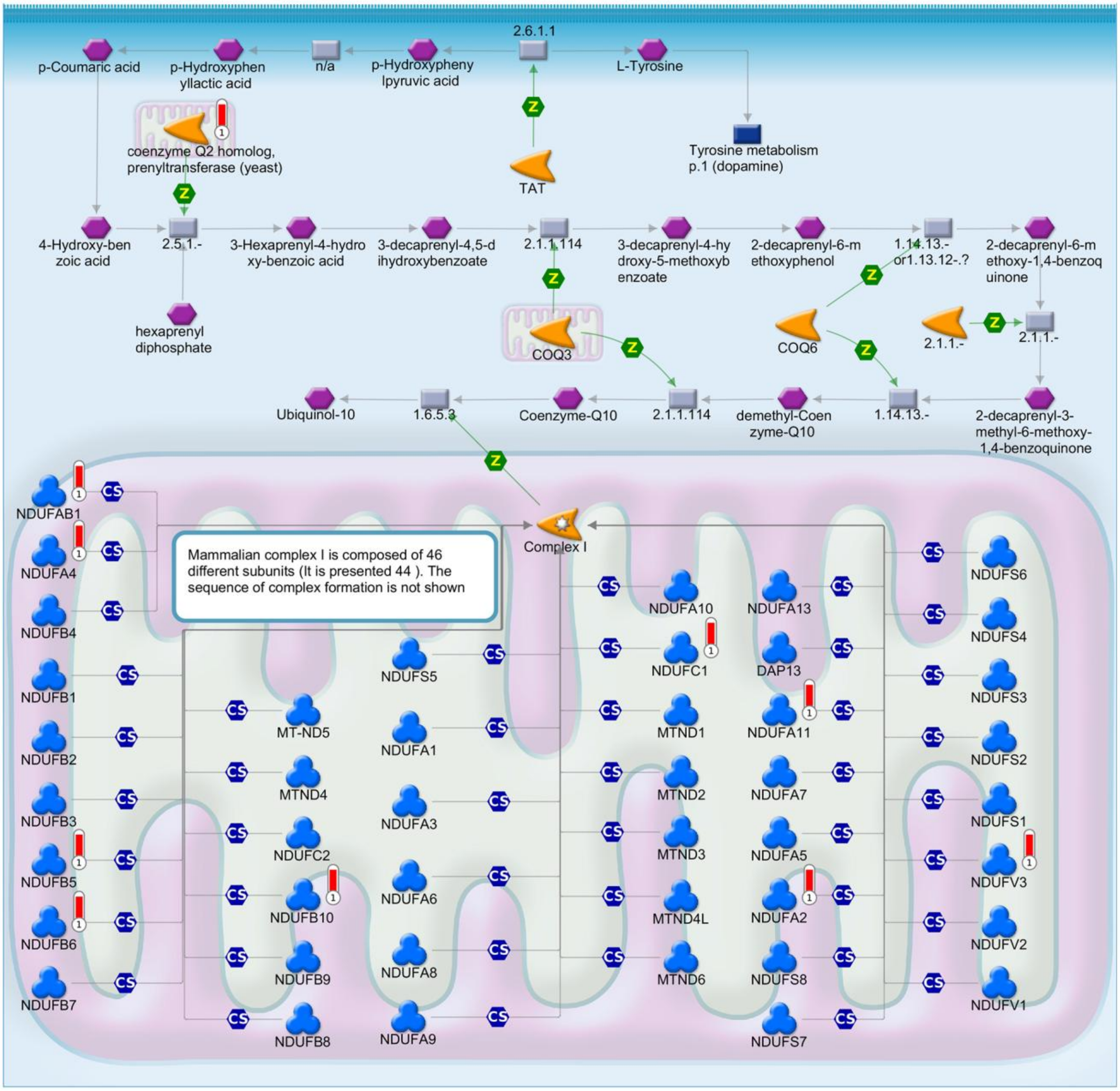

Supplementary Figure 5. Ubiquinone-related networks were correlated with proteasome 26S subunit, ATPase (PSMC) family genes in breast cancer. The MetaCore pathway analysis of "biological processes" indicated that "Ubiquinone metabolism"-related pathways were correlated with PSMC family genes in breast cancer development.

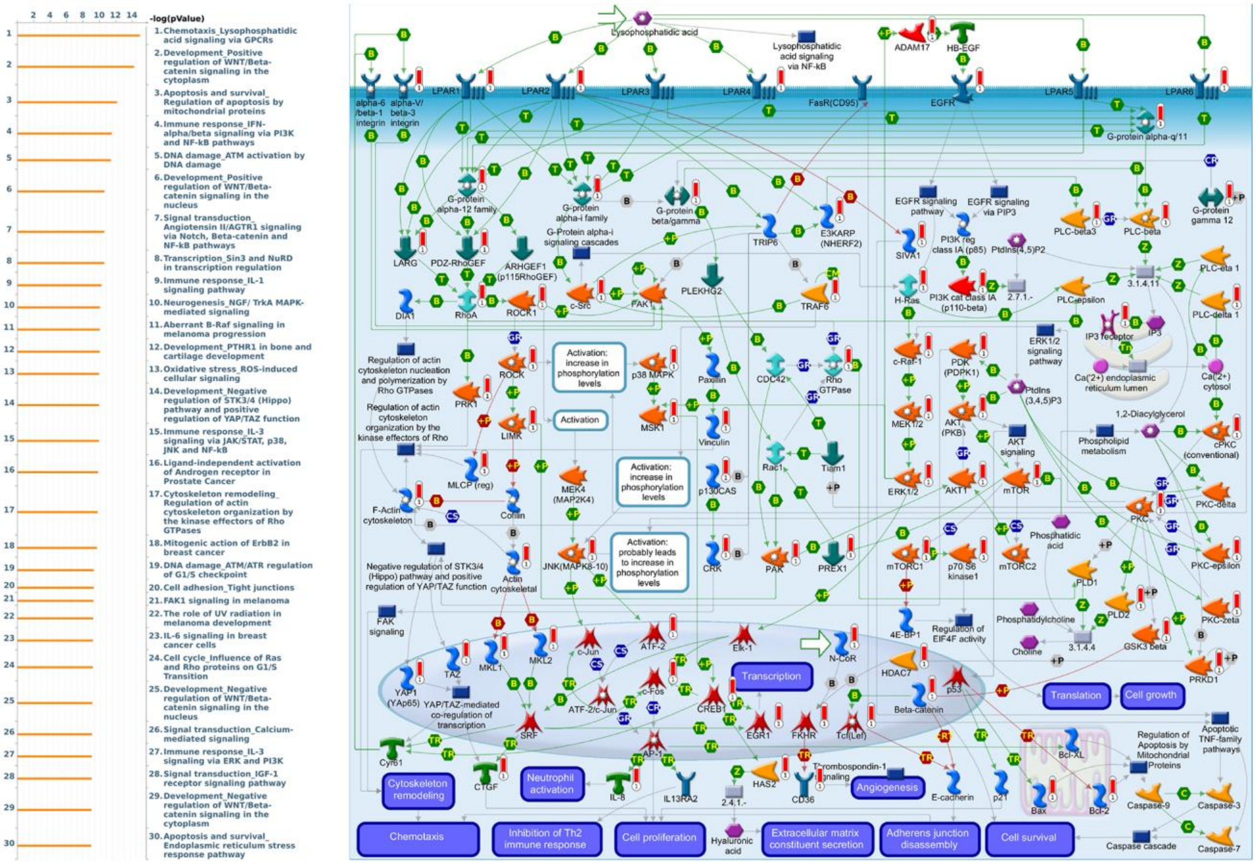

Supplementary Figure 6. MetaCore pathway analysis of the coexpression gene network of proteasome 26S subunit, ATPase 1 (PSMC1) in breast cancer patients. Downstream pathway analyses revealed that "Chemotaxis_Lysophosphatidic acid signaling via GPCRs" might play an important role in breast cancer development.

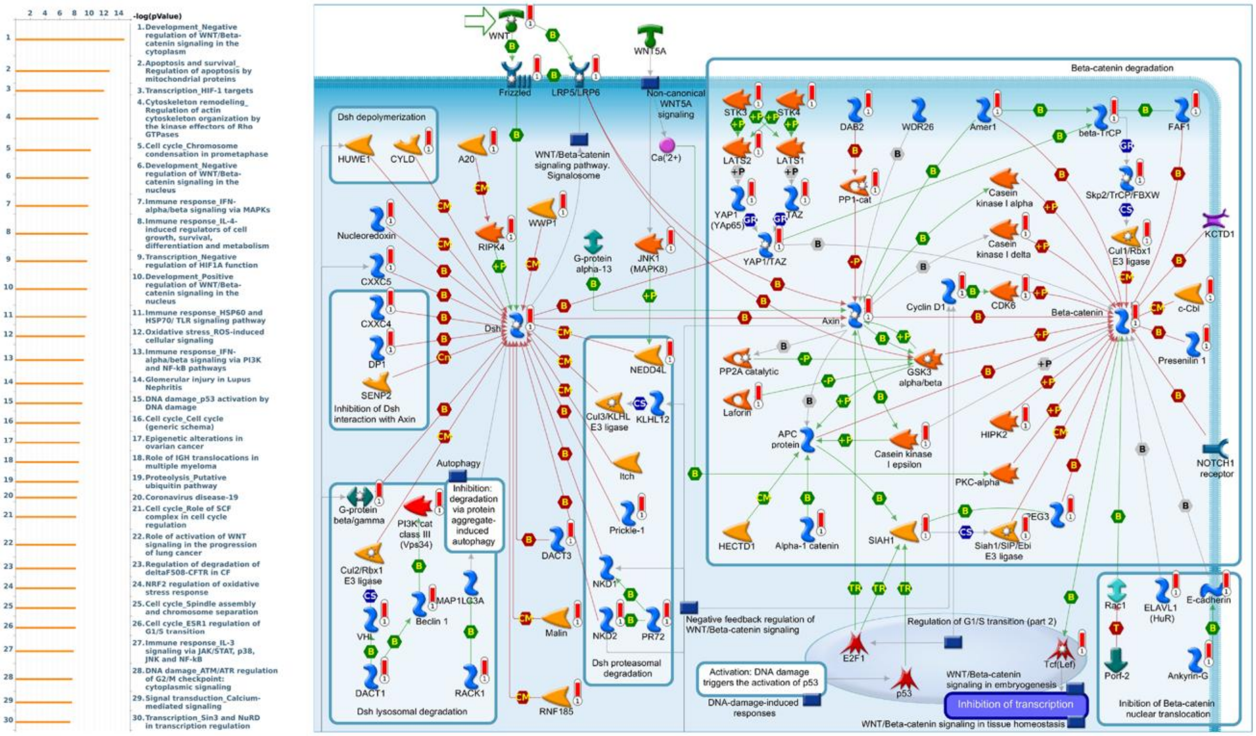

Supplementary Figure 7. MetaCore pathway analysis of the coexpression gene network of proteasome 26S subunit, ATPase 2 (PSMC2) in breast cancer patients. Downstream pathway analyses revealed that "Development_Negative regulation of WNTBeta-catenin signaling in the cytoplasm" might play an important role in breast cancer development.

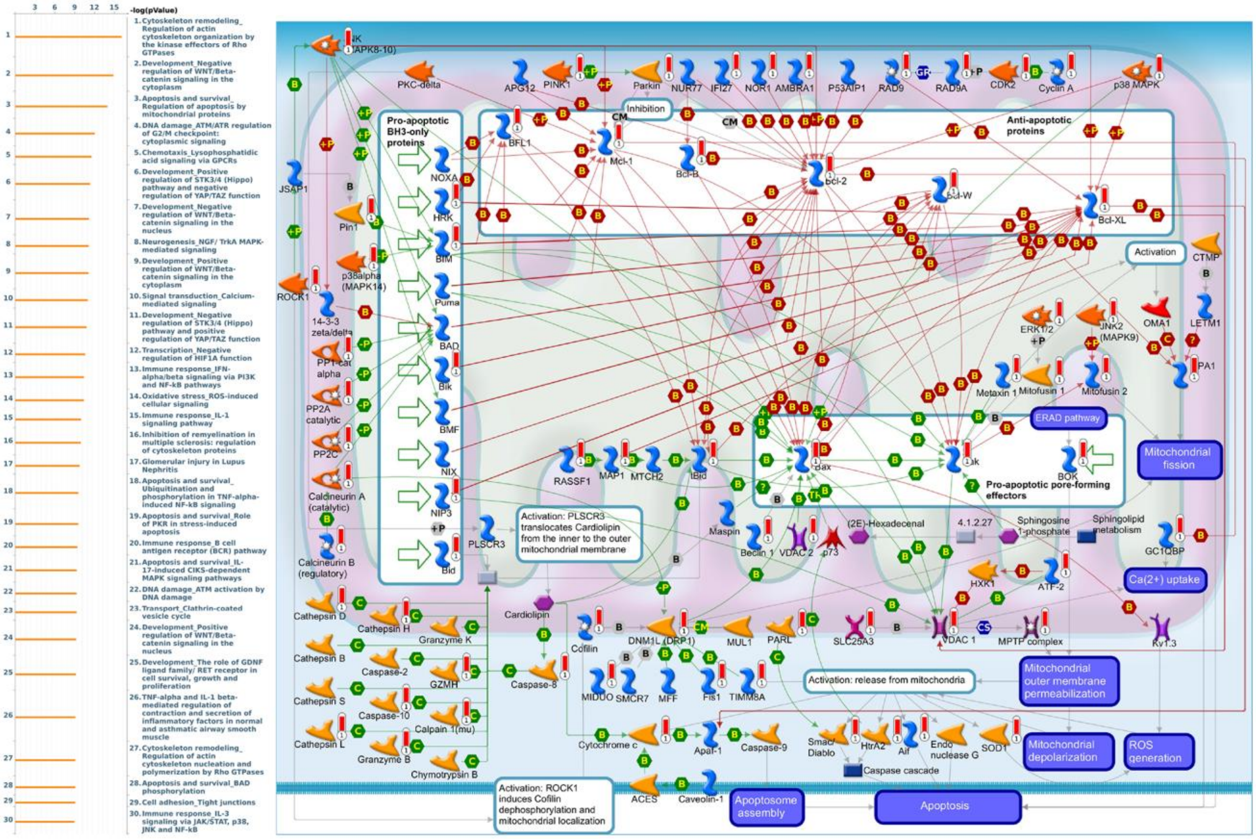

Supplementary Figure 8. MetaCore pathway analysis of the coexpression gene network of proteasome 26S subunit, ATPase 3 (PSMC3) in breast cancer patients. Downstream pathway analyses revealed that "Apoptosis and survival_Regulation of apoptosis by mitochondrial proteins" might play an important role in breast cancer development.

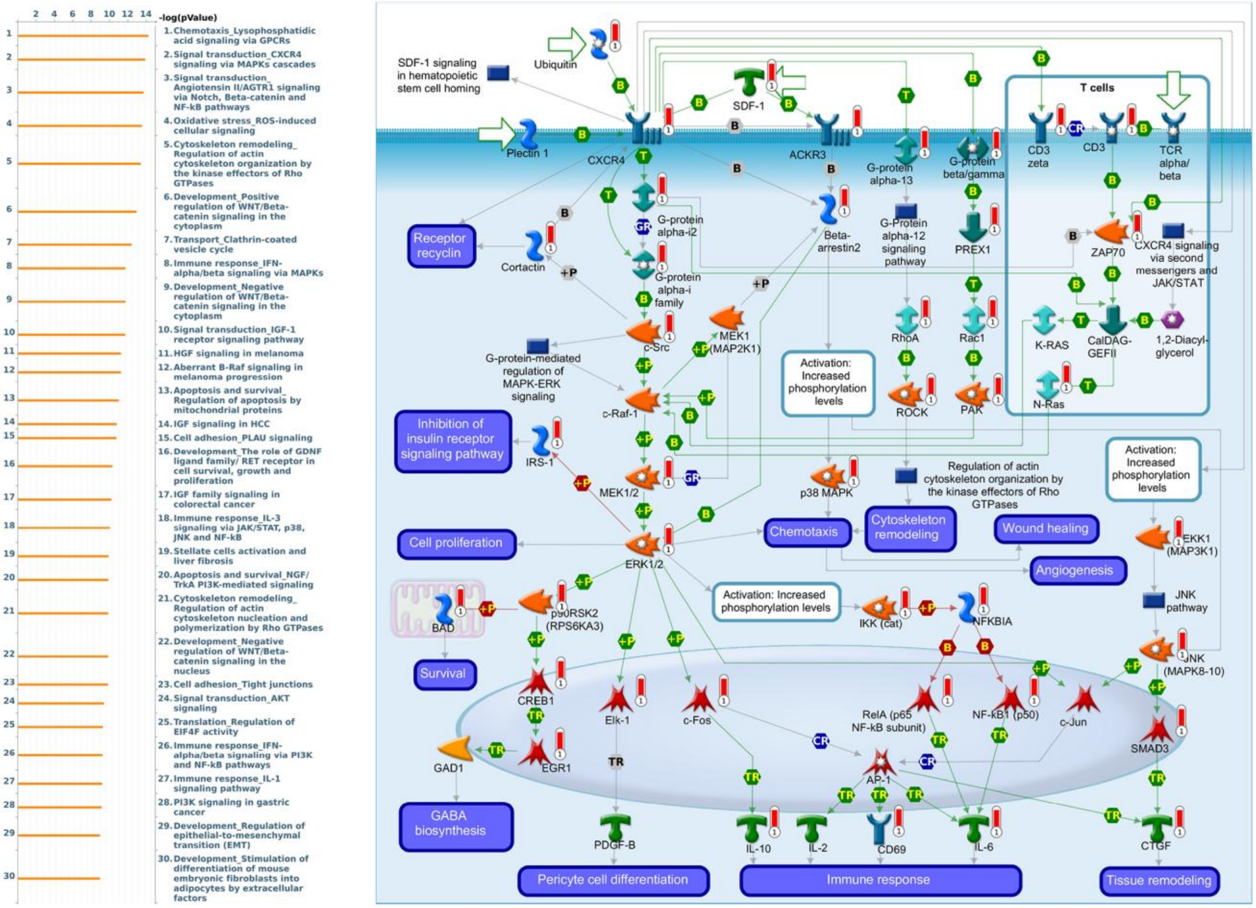

Supplementary Figure 9. MetaCore pathway analysis of the coexpression gene network of proteasome 26S subunit, ATPase 4 (PSMC4) in breast cancer patients. Downstream pathway analyses revealed that "Signal transduction_CXCR4 signaling via MAPK cascades" might play an important role in breast cancer development.

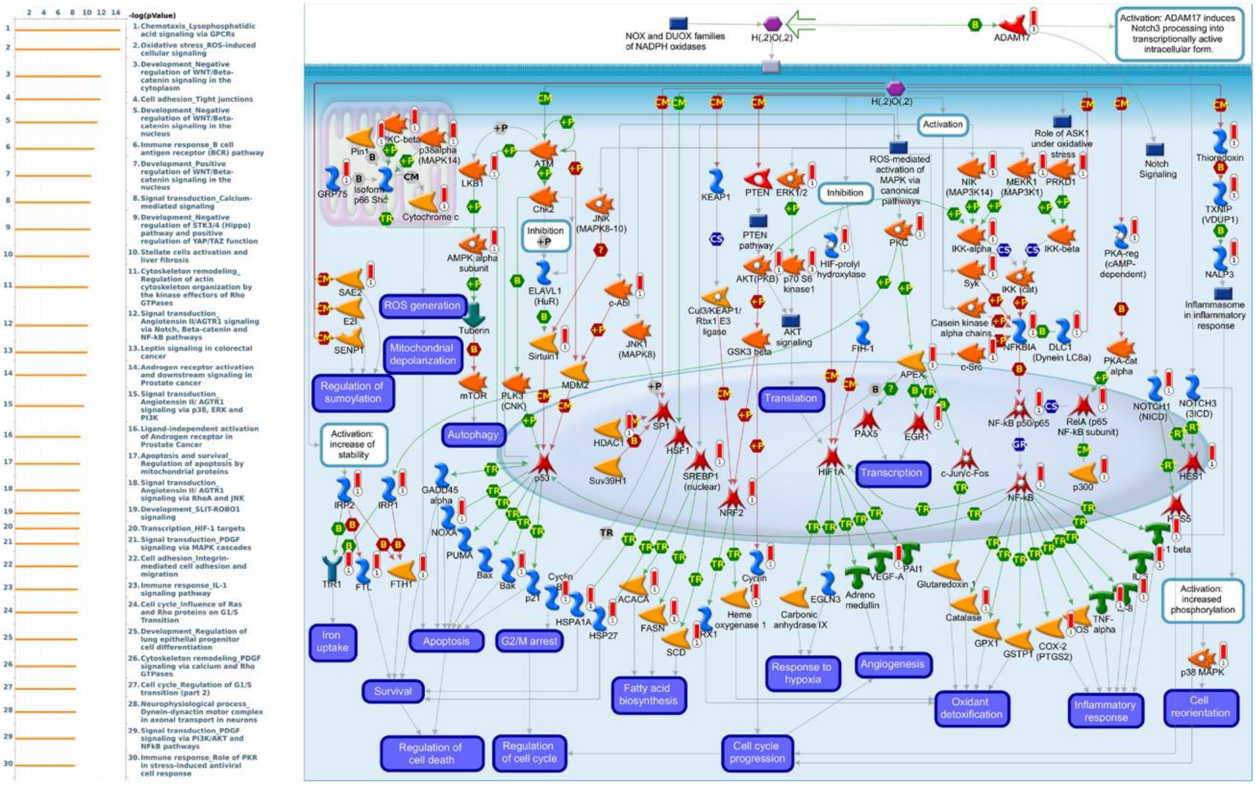

Supplementary Figure 10. MetaCore pathway analysis of the genetic network coexpressed with proteasome 26S subunit, ATPase 5 (PSMC5) in breast cancer patients. Downstream pathway analyses revealed that "Oxidative stress_ROS-induced cellular signaling" might play an important role in breast cancer development.

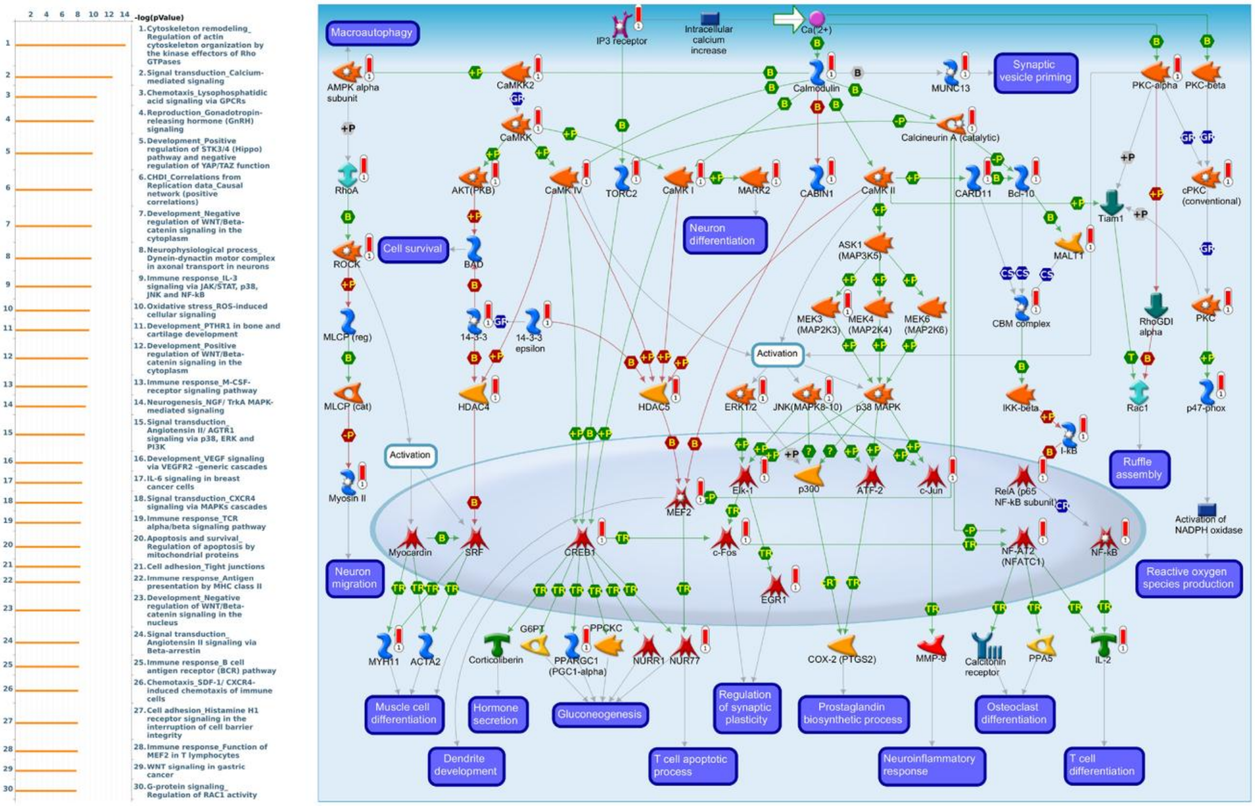

Supplementary Figure 11. MetaCore pathway analysis of the genetic network coexpressed with proteasome 26S subunit, ATPase 6 (PSMC6) in breast cancer patients. Downstream pathway analyses revealed that "Signal transduction_Calcium-mediated signaling" might play an important role in breast cancer development.

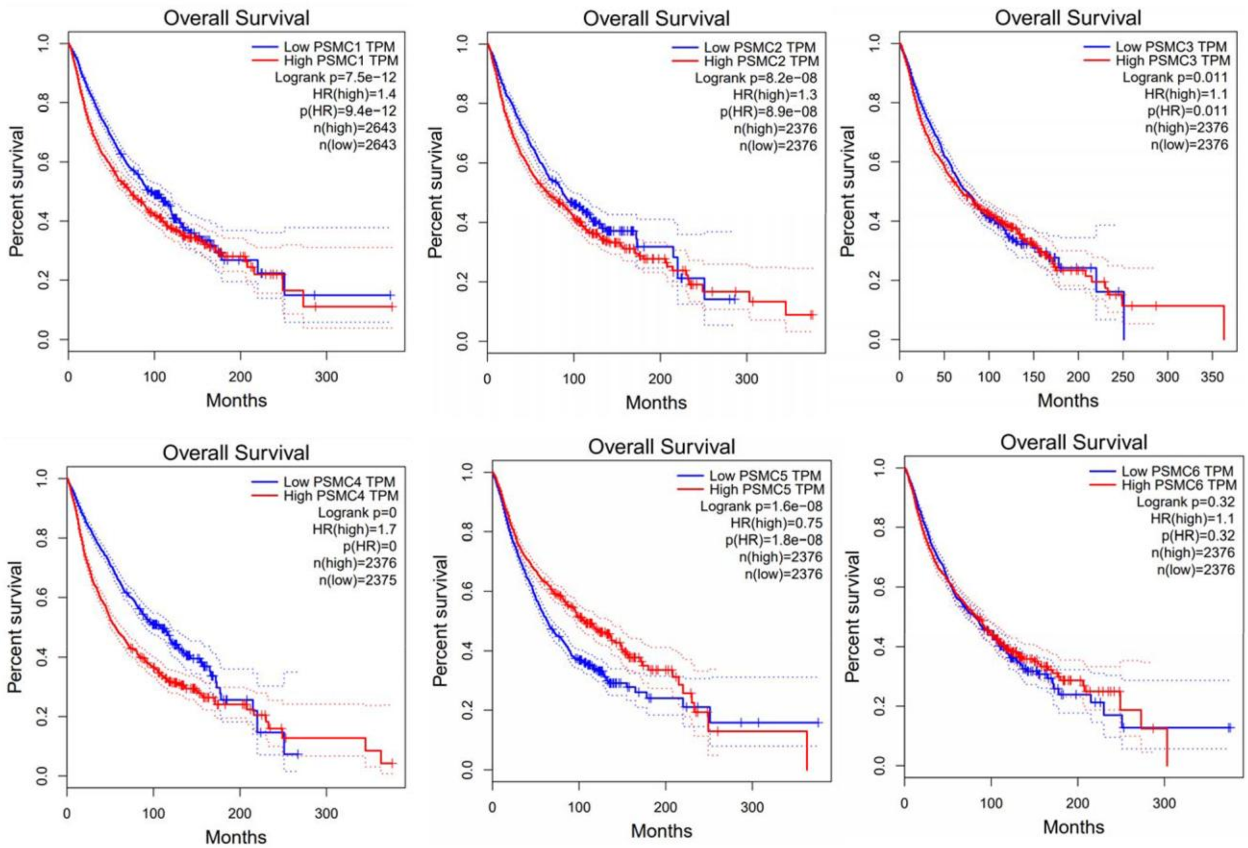

Supplementary Figure 12. Overall survival curves comparing the high and low expression of PSMC family genes across different types of cancer. The survival map for PSMC family genes in pan-cancer analyzed in GEPIA2 platform, and p<0.05 considered significant.

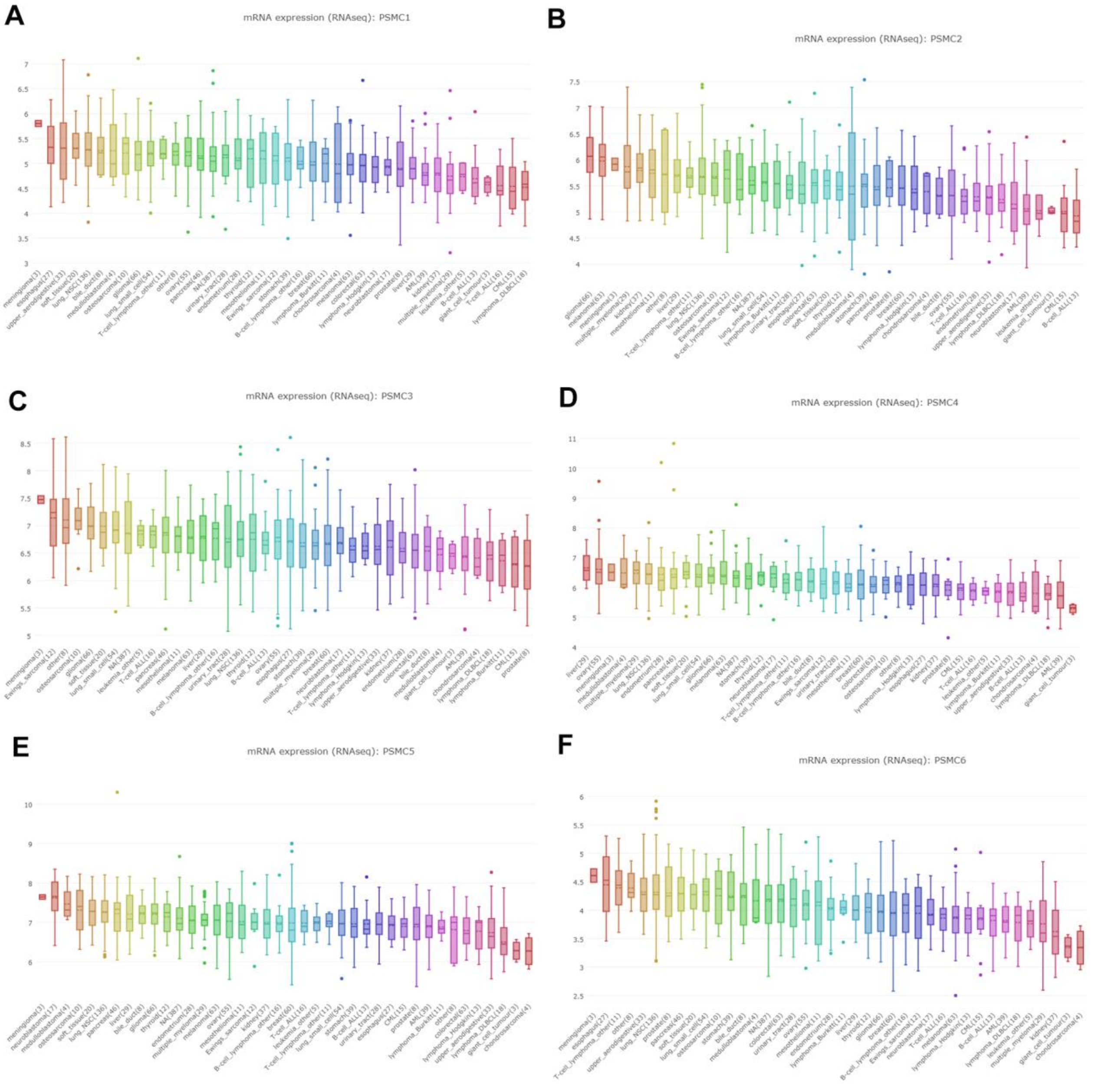

Supplementary Figure 13. The transcript expression of PSMC family genes in a variety of cancer cell lines. (**A**) The differential expression of PSMC1 in Cancer Cell Line Encyclopedia (CCLE). (**B**) The differential expression of PSMC2 in CCLE. (**C**) The differential expression of PSMC3 in in CCLE. (**D**) The differential expression of PSMC4 in CCLE. (**E**) The differential expression of PSMC5 in CCLE. (**F**) The differential expression of PSMC6 in CCLE.
