# Supplementary material for: Prognoses and genomic analyses of proteasome 26S subunit, ATPase (PSMC) family genes in clinical breast cancer
Source: Aging (Albany NY). 2021 Jul 30;13(14):17970. doi: 10.18632/aging.203345 (PMC8351721; doi:10.18632/aging.203345)
Supplement: Supplementary Tables 1 and 2 [file aging-13-203345-s002.pdf]

SUPPLEMENTARY TABLES

Supplementary Table 1. Significant changes in transcription levels of the proteasome 26S subunit, ATPase (PSMC) family genes in different types of breast cancer from the METABRIC database.

Gene	Types of sarcoma vs. normal	*p* value (cancer/normal)	*t*-test (cancer/normal)	Multiple of change (cancer/normal)	% Gene ranking
**PSMC1**	Invasive ductal breast carcinoma	2.16E-45	17.213	1.239	1776 (in top 10%)
	Invasive lobular breast carcinoma	3.73E-15	8.299	1.186	2610 (in top 14%)
	Invasive ductal and invasive lobular breast carcinoma	2.32E-12	7.567	1.186	2619 (in top 14%)
	Tubular breast carcinoma	5.85E-10	6.657	1.156	3135 (in top 17%)
	Medullary breast carcinoma	6.09E-09	7.432	1.43	1189 (in top 7%)
	Invasive breast carcinoma	5.63E-04	3.76	1.249	2613 (in top 14%)
	Ductal breast carcinoma in situ	1.50E-02	2.516	1.13	4252 (in top 23%)
	Benign breast neoplasm	2.30E-02	3.543	1.172	1385 (in top 8%)
**PSMC2**	Invasive ductal breast carcinoma	1.37E-29	13.032	1.212	3117 (in top 17%)
	Tubular breast carcinoma	4.52E-10	6.731	1.206	3098 (in top 17%)
	Medullary breast carcinoma	2.74E-07	6.158	1.479	1898 (in top 10%)
	Invasive ductal and invasive lobular breast carcinoma	2.84E-07	5.278	1.189	4496 (in top 24%)
	Invasive lobular breast carcinoma	2.52E-04	3.521	1.08	6711 (in top 35%)
	Mucinous breast carcinoma	4.81E-04	3.453	1.11	5503 (in top 29%)
	Ductal breast carcinoma in situ	2.00E-03	3.814	1.224	1815 (in top 10%)
	Invasive breast carcinoma	1.10E-02	2.468	1.164	4963 (in top 26%)
	Breast carcinoma	1.60E-02	2.378	1.149	4844 (in top 26%)
**PSMC3**	Invasive ductal breast carcinoma	1.81E-56	21.593	1.391	1165 (in top 7%)
	Invasive lobular breast carcinoma	3.18E-21	10.223	1.268	1647 (in top 9%)
	Invasive ductal and invasive lobular breast carcinoma	1.18E-14	8.518	1.283	2072 (in top 11%)
	Tubular breast carcinoma	1.54E-10	6.928	1.213	2948 (in top 16%)
	Mucinous breast carcinoma	2.35E-09	6.759	1.251	2118 (in top 11%)
	Medullary breast carcinoma	5.08E-09	7.49	1.581	1167 (in top 7%)
	Invasive breast carcinoma	1.04E-05	5.336	1.334	995 (in top 6%)
	Breast carcinoma	7.67E-04	3.846	1.207	2135 (in top 12%)
	Ductal breast carcinoma in situ	1.00E-03	3.932	1.328	1734 (in top 9%)
**PSMC4**	Invasive ductal breast carcinoma	1.28E-53	20.067	1.663	1299 (in top 7%)
	Invasive lobular breast carcinoma	4.00E-21	10.233	1.505	1665 (in top 9%)
	Invasive ductal and invasive lobular breast carcinoma	8.95E-21	10.973	1.704	1064 (in top 6%)
	Mucinous breast carcinoma	3.44E-20	12.783	1.953	138 (in top 1%)
	Tubular breast carcinoma	2.41E-11	7.464	1.468	2693 (in top 14%)
	Breast carcinoma	9.68E-06	6.234	1.766	501 (in top 3%)
	Ductal breast carcinoma in situ	7.54E-05	5.948	1.877	375 (in top 2%)
	Invasive breast carcinoma	2.24E-04	4.167	2.021	2119 (in top 11%)
	Medullary breast carcinoma	1.00E-03	3.259	1.381	4759 (in top 25%)
	Benign breast neoplasm	1.60E-02	5.134	2.085	1096 (in top 6%)
	Breast phyllodes tumor	1.80E-02	3.029	1.637	2003 (in top 11%)
**PSMC5**	Invasive ductal breast carcinoma	7.02E-36	15.397	1.431	2506 (in top 14%)
	Invasive lobular breast carcinoma	6.11E-13	7.435	1.274	3086 (in top 17%)
.	Tubular breast carcinoma	3.05E-12	7.714	1.403	2414 (in top 13%)
	Invasive ductal and invasive lobular breast carcinoma	5.95E-12	7.294	1.327	2733 (in top 15%)
	Mucinous breast carcinoma	1.09E-09	6.884	1.393	1996 (in top 11%)
	Invasive breast carcinoma	7.93E-06	5.41	1.518	916 (in top 5%)
	Medullary breast carcinoma	5.07E-04	3.559	1.273	4293 (in top 23%)
	Ductal breast carcinoma in situ	2.00E-03	3.797	1.493	1893 (in top 10%)
	Breast carcinoma	8.00E-03	2.758	1.317	4011 (in top 21%)
	Benign breast neoplasm	4.10E-02	3.005	1.297	2135 (in top 12%)
**PSMC6**	Invasive ductal breast carcinoma	9.03E-12	6.909	1.065	5885 (in top 31%)
	Tubular breast carcinoma	1.96E-07	5.569	1.173	4195 (in top 22%)
	Invasive ductal and invasive lobular breast carcinoma	6.40E-04	3.303	1.14	6817 (in top 36%)
	Invasive lobular breast carcinoma	3.00E-03	2.815	1.052	7600 (in top 40%)
	Mucinous breast carcinoma	4.00E-03	2.76	1.05	6596 (in top 35%)
	Breast carcinoma	7.00E-03	2.771	1.061	3885 (in top 21%)
	Ductal breast carcinoma in situ	3.90E-02	1.965	1.068	5699 (in top 30%)

Supplementary Table 2. Pathway analysis of genes co-expressed with proteasome 26S subunit, ATPase (PSMC) family genes from public breast cancer databases using the MetaCore database (with p<0.01 set as the cutoff value).

#	Map	*p* Value	Network objects from active data
**1**	Cytoskeleton remodeling_Regulation of actin cytoskeleton organization by the kinase effectors of Rho GTPases	6.29E-07	ARPC1B, BETA-PIX, Talin, Vinculin, ERM proteins, MyHC, DMPK, Spectrin, MLCK, MRLC, Rac1-related
**2**	Cell cycle_Chromosome condensation in prometaphase	1.24E-06	CAP-G, Cyclin A, CAP-G/G2, Aurora-A, Cyclin B, Histone H3, CDK1 (p34)
**3**	Cell cycle_Nucleocytoplasmic transport of CDK/Cyclins	1.32E-06	Importin (karyopherin)-alpha, Cyclin A, Cyclin D1, Cyclin D3, Cyclin D, CDK1 (p34)
**4**	Transcription_Role of heterochromatin protein 1 (HP1) family in transcriptional silencing	1.48E-06	SUMO-1, Cyclin A2, HDAC4, Rb protein, MEF2, Histone H4, HDAC5, Histone H3, CDK1 (p34)
**5**	Cell cycle_Role of APC in cell cycle regulation	2.43E-06	Geminin, Cyclin A, Aurora-A, Kid, Cyclin B, MAD2a, Securin, CDK1 (p34)
**6**	Cell cycle_Spindle assembly and chromosome separation	3.13E-06	Importin (karyopherin)-alpha, Aurora-A, HEC, Kid, Cyclin B, MAD2a, Securin, CDK1 (p34)
**7**	Cell cycle_Regulation of G1/S transition (part 1)	9.74E-06	Cyclin A, Skp2/TrCP/FBXW, Cyclin D1, TGF-beta receptor type II, RING-box protein 1, SMAD4, Cyclin D3, Cyclin D
**8**	Cell cycle_Regulation of G1/S transition (part 2)	1.06E-05	Cyclin A2, Cyclin A, Cyclin D1, Rb protein, Cyclin D3, Cyclin D, c-Fos
**9**	Abnormalities in cell cycle in SCLC	1.36E-05	PCNA, Cyclin A, Cyclin D1, Rb protein, Histone H3, CDK1 (p34), Cyclin E2
**10**	Prolactin/JAK2 signaling in breast cancer	4.63E-05	Bcl-6, STAT5, Cyclin D1, STAT5B, PKM2, Cyclophilin A
**11**	Ubiquinone metabolism	4.65E-05	NDUFAB1, NDUFA4, NDUFB6, NDUFA2, NDUFA11, coenzyme Q2 homolog, prenyltransferase (yeast), NDUFB5, NDUFB10, NDUFC1, NDUFV3
**12**	Cell cycle_The metaphase checkpoint	6.12E-05	SPBC25, Aurora-A, HEC, CDCA1, HZwint-1, MAD2a, CENP-H
**13**	Transport_The role of AVP in regulation of Aquaporin 2 and renal water reabsorption	7.92E-05	MRLC2, MyHC, SNAP-23, Annexin II, VAMP2, MLCK, MRLC, c-Fos
**14**	Immune response_Function of MEF2 in T lymphocytes	7.92E-05	MAP3K3, MEF2D, HDAC4, Calcineurin A (catalytic), MEF2, HDAC5, MEF2C, Calcineurin A (beta)
**15**	Cell cycle progression in Prostate Cancer	1.05E-04	Beta-catenin, Cyclin D1, STAT5B, Rb protein, Cyclin B, CDK1 (p34), c-Fos
**16**	Immune response_ETV3 effect on CSF1-promoted macrophage differentiation	1.57E-04	MSK1/2 (RPS6KA5/4), ETV3, PRIM2A, HDAC5, CDK1 (p34)
**17**	Angiotensin II Signaling in Cardiac Hypertrophy	2.05E-04	Thioredoxin, CBP, HDAC4, Calcineurin A (catalytic), NF-kB, HDAC5, SOD1, TRPC1, c-Fos
**18**	Cell cycle_Cell cycle (generic schema)	2.63E-04	Cyclin A, Rb protein, Cyclin B, Cyclin D, CDK1 (p34)
**19**	Signal transduction_Activin A signaling regulation	3.07E-04	CBP, Histone H2, SMAD4, TGF-beta receptor type III (betaglycan), Histone H4, Histone H3
**20**	Aminoglycoside- and cisplatin-induced hair cell death	3.65E-04	Calpain 1(mu), Cytochrome c, Beta-catenin, Histone H2A, HDAC4, Calcineurin A (catalytic), Histone H2B, Histone H4, NF-kB, Histone H3, c-Fos
**21**	Cell cycle_Sister chromatid cohesion	4.14E-04	PCNA, Cyclin B, Securin, Histone H3, CDK1 (p34)
**22**	Noise-induced hair cell death and spiral ganglion neuron degeneration	4.18E-04	Calpain 1(mu), Cytochrome c, Alpha-fodrin, VAMP1, GDNF, ERM proteins, HDAC4, Rb protein, Calcineurin A (catalytic), Histone H3
**23**	Cell cycle_ESR1 regulation of G1/S transition	4.28E-04	Cyclin A2, Cyclin A, Skp2/TrCP/FBXW, Cyclin D1, Rb protein, c-Fos
**24**	Transcription_N-CoR/SMRT complex-mediated epigenetic gene silencing	4.58E-04	PBX1, HDAC4, Histone H2B, Histone H4, NF-kB, HDAC5, c-Fos
**25**	HBV-dependent transcription regulation leading to HCC	6.24E-04	PCNA, CBP, EGR2 (Krox20), Cyclin D1, Pin1
**26**	Development_Role of HDAC and calcium/calmodulin-dependent kinase (CaMK) in control of skeletal myogenesis	7.45E-04	MAP3K3, MEF2D, HDAC4, Calcineurin A (catalytic), MEF2, HDAC5, MEF2C
**27**	CREB1-dependent transcription deregulation in Huntington's Disease	7.55E-04	Cytochrome c, CBP, NDUFB5, COX VIa-1, SOD1
**28**	Development_NOTCH1-mediated pathway for NF-KB activity modulation	7.55E-04	Jagged1, MAML1, Histone H4, NF-kB, Histone H3
**29**	Apoptosis and survival_Ubiquitination and phosphorylation in TNF-alpha-induced NF-kB signaling	7.81E-04	UEV1A, MAP3K3, E2N(UBC13), TRADD, NF-kB1 (p105), NF-kB1 (p50)
**30**	Mitogenic action of ErbB2 in breast cancer	1.04E-03	Beta-catenin, Cyclin G2, Cyclin D1, MSK1, ErbB4, NF-kB, c-Fos
