# Supplementary material for: Prognoses and genomic analyses of proteasome 26S subunit, ATPase (PSMC) family genes in clinical breast cancer
Source: Aging (Albany NY). 2021 Jul 30;13(14):17970. doi: 10.18632/aging.203345 (PMC8351721; doi:10.18632/aging.203345)
Supplement: Supplementary Table 3 [file aging-13-203345-s003.docx]

**Supplementary Table 3. Pathway analysis of genes coexpressed with proteasome 26S subunit, ATPase 1 (PSMC1) from public breast cancer databases using the MetaCore database (with p<0.01 set as the cutoff value).**

| # | Map | *p* value | Network objects from active data |
| --- | --- | --- | --- |
| 1 | Chemotaxis_Lysophosphatidic acid signaling via GPCRs | 1.12E-15 | AKT1, c-Fos, PI3K cat class IA (p110-beta), H-Ras, LARG, ROCK1, ERK1/2, PRK1, PKC-zeta, Beta-catenin, EGR1, CRK, c-Raf-1, HDAC7, G-protein alpha-q/11, PKC-epsilon, IP3 receptor, YAP1 (YAp65), LPAR2, MLCP (reg), LPAR4, Bax, GSK3 beta, PLC-beta, FKHR, HAS2, LPAR6, alpha-V/beta-3 integrin, CREB1, MEK1/2, G-protein beta/gamma, Actin cytoskeletal, N-CoR, TAZ, MSK1, AKT(PKB), PDK (PDPK1), c-Src, RhoA, cPKC (conventional), G-protein alpha-12 family, LPAR1, Tcf(Lef), IL-8, EGFR, G-protein alpha-i family, TRAF6, F-Actin cytoskeleton, MKL2, mTORC1, LIMK, PLD2, CD36, E3KARP (NHERF2), G-protein gamma 12, p130CAS, ROCK, PRKD1, PAK, p70 S6 kinase1, PDZ-RhoGEF, PKC, Vinculin, ATF-2, PLC-delta 1, Bcl-2, SIVA1, Rho GTPase, JNK(MAPK8-10), MKL1, CTGF, PLC-beta3, ADAM17, p38 MAPK, mTOR, PREX1 |
| 2 | Development_Positive regulation of WNT/Beta-catenin signaling in the cytoplasm | 5.88E-15 | TBL1X, Bcl-9, EGF, IRS-2, IRS-1, PP1-cat, Alpha-1 catenin, 14-3-3, Beta-catenin, TGIF, SMAD4, Dsh, USP47, ZBED3, Beta-arrestin2, YAP1 (YAp65), CDK1 (p34), PPP2R2A, USP25, GSK3 alpha/beta, IGF-1 receptor, ERK2 (MAPK1), AKT(PKB), RNF146, GSKIP, ITGB1, LRP5/LRP6, SIAH2, HIPK2, Tcf(Lef), HSP105, PKA-reg type II (cAMP-dependent), PP2A catalytic, RNF220, ILK, DOCK4, Trabid, Tankyrases, BIG2, Miz-1, USP9X, WNT, Jouberin, PP2C alpha, JNK(MAPK8-10), SMAD3, Parathyroid hormone, MITF, PKA-cat (cAMP-dependent), Frizzled, SET7, DACT1 |
| 3 | Apoptosis and survival_Regulation of apoptosis by mitochondrial proteins | 7.58E-13 | p38alpha (MAPK14), NIX, Bcl-W, PLSCR3, Calcineurin A (catalytic), MPTP complex, Apaf-1, Cathepsin H, Granzyme B, ROCK1, ERK1/2, BFL1, RAD9A, PP2C, VDAC 2, Cytochrome c, MFF, Endonuclease G, NIP3, BAD, Bax, OMA1, SOD1, PP1-cat alpha, AMBRA1, Mitofusin 1, MTCH2, VDAC 1, MIDUO, Calpain 1(mu), Mcl-1, Cathepsin D, Cathepsin L, IFI27, Aif, GC1QBP, Fis1, Pin1, PP2A catalytic, PARL, Metaxin 1, JSAP1, RAD9, Cathepsin B, Bcl-B, ATF-2, SLC25A3, TIMM8A, tBid, Bcl-2, Beclin 1, HtrA2, Cyclin A, JNK(MAPK8-10), JNK2(MAPK9), Mitofusin 2, Caveolin-1, Calcineurin B (regulatory), Smac/Diablo, p38 MAPK, Bim, Bid |
| 4 | Immune response_IFN-alpha/beta signaling via PI3K and NF-kB pathways | 3.43E-12 | Tuberin, AKT1, Cyclin D3, JAK1, IRS-2, IL-10, Tyk2, IRS-1, RPS6, NMI, ERK1/2, c-Raf-1, CDC25A, PKC-epsilon, CDK1 (p34), I-kB, p70 S6 kinases, GSK3 beta, NF-kB2 (p100), Rb protein, CDK4, EMSY, p19, CREB1, p130, MEK1/2, RSAD2, NF-kB, AKT(PKB), PDK (PDPK1), p16INK4, p107, p27KIP1, c-Myc, SLFN5, IFI17, PI3K cat class IA, RelA (p65 NF-kB subunit), NF-kB2 (p52), TRAF2, I-TAC, mTORC1, eIF4B, p90RSK1, eIF4G1/3, NIK(MAP3K14), STAT3, PCNA, GBP1, IFIT1, PKC-alpha, Cyclin E, eIF4A, FOXO3A, IRF7, Cyclin A |
| 5 | DNA damage_ATM activation by DNA damage | 4.4E-12 | CDK5R1 (p35), p14ARF, CDK5, BRAT1, OBFC2B, Aven, PP2A regulatory, p90RSK2(RPS6KA3), E2N(UBC13), Mob2 (HCCA2), Tip60, HMG14, HDAC2, SOSSC, MYST1, Itch, MDM2, RNF8, RecQL4, CHFR, HSP90, Calpain 1(mu), Histone H1.2, NDR1 (STK38), RCAD, Brca1, Histone H3, EGFR, RAD17, INTS3, Histone H2B, CDK5R1 (p25), Histone H2AX, PP2A catalytic, HSP90 beta, p18, MRE11, Pellino 1, DMAP1, MRG15, NK31, FOXO3A, c-Abl, TELO2, eIF3S6, Histone H4 |
| 6 | Development_Positive regulation of WNT/Beta-catenin signaling in the nucleus | 2.85E-11 | SMYD2, TBL1X, CBP, EGF, Alpha-1 catenin, TCF7L2 (TCF4), FOXP1, USP5, Jade-1, Beta-catenin, BCL9/B9L, FOXK1, TLE, ZIP-kinase, Dsh, Casein kinase I alpha, UBR5, YAP1 (YAp65), GSK3 beta, NRARP, HDAC2, WIP1, PCAF, RUNX, ICAT, ERK2 (MAPK1), SOX9, JRK, LRP5/LRP6, TWA1, Tcf(Lef), beta-TrCP, FOXM1, Pin1, NCOA2 (GRIP1/TIF2), FAM53B, NLK, Kindlin-2, CARF, WNT, FOXO3A, HMGB2, APPL, Frizzled |
| 7 | Signal transduction_Angiotensin II/AGTR1 signaling via Notch, Beta-catenin and NF-kB pathways | 2.95E-11 | CBP, NOTCH1 receptor, CCL2, TCF7L2 (TCF4), ROCK1, ERK1/2, Beta-catenin, TRPC6, YAP1 (YAp65), I-kB, GSK3 beta, NF-kB2 (p100), IGF-1 receptor, NF-kB, ERK2 (MAPK1), AKT(PKB), NF-kB p50/p65, RBP-J kappa (CBF1), PDK (PDPK1), c-Myc, TAK1(MAP3K7), NOTCH1 (NICD), RhoA, RelA (p65 NF-kB subunit), NF-kB2 (p52), IL-8, TRAF6, p90RSK1, PAK1, NIK(MAP3K14), NOTCH3 (3ICD), PRKD1, gamma-Secretase complex, PKC, WISP1, IKK-beta, Connexin 43, Cyclin D1, SNAIL1, NCOA1 (SRC1), CTGF, NOTCH1 (NEXT), ADAM17, p38 MAPK, PKA-cat (cAMP-dependent), mTOR, NOTCH3 |
| 8 | Transcription_Sin3 and NuRD in transcription regulation | 2.99E-11 | TR-alpha, RARbeta, SMRT, SAP130, RBBP4 (RbAp48), ARID4A, MBD2, HDAC2, Mi-2 beta, NRSF, RARalpha, N-CoR, ARID4B, p66beta, RAR-alpha/TR-alpha, MTA1, Mi-2, Histone H3, Sin3A, RXRA, RAR-beta/RXR-alpha, RAR-alpha/RXR-alpha, MTA2, RBBP7 (RbAp46), Sin3B, SDS3, SAP30, MBD3, NRB54, p66alpha, Histone H4 |
| 9 | Immune response_IL-1 signaling pathway | 6.6E-11 | SPHK1, p38alpha (MAPK14), JAM2, IL-1 alpha, IP10, ICAM1, CD44, CCL2, ERK1/2, PKC-zeta, EGR1, MEK4/7, CCL5, I-kB, NF-kB2 (p100), MAPKAPK2, GRO-1, NF-kB p52/RelB, MEK1/2, TPL2(MAP3K8), NF-kB, AKT(PKB), NF-kB p50/p65, PDK (PDPK1), MYLK1, TAK1(MAP3K7), Sequestosome 1(p62), c-IAP2, PI3K cat class IA, RelA (p65 NF-kB subunit), MMP-13, NF-kB1 (p105), NF-kB1 (p50), NF-kB2 (p52), IL-8, ZFP36(Tristetraprolin), TRAF6, MEK3(MAP2K3), IL1RAP, PGES, NIK(MAP3K14), PLAU (UPA), CCL7, RUNX2, MEKK1(MAP3K1), MAP3K3, HSP27, IRAK1, JNK(MAPK8-10) |
| 10 | Neurogenesis_NGF/ TrkA MAPK-mediated signaling | 1.02E-10 | SPHK1, CDK5R1 (p35), CDK5, c-Fos, APS, Fra-1, H-Ras, ERK1/2, MEF2C, PKC-zeta, CrkL, EGR1, PP2A regulatory, PKA-reg (cAMP-dependent), CRK, c-Raf-1, FRS2, PKC-epsilon, Ephrin-A receptor 2, IP3 receptor, MAPKAPK2, N-Ras, MAP2K5 (MEK5), KIDINS220, CREB1, MEK1/2, PLAUR (uPAR), Flotillin-1, MSK1, RUSC1 (NESCA), p107, c-Src, Sequestosome 1(p62), MATK, Efs/Sin, RIN, B-Raf, NF-kB1 (p50), MEK3(MAP2K3), SOS, PP2A catalytic, C3G, SH2B, p90Rsk, p130CAS, Calmodulin, SHP-2, SUR-8, Cyclin D1, RASGRF1, SP1, PVR, JMJD3, SHB, RIT, p38 MAPK, PKA-cat (cAMP-dependent), FosB |
| 11 | Aberrant B-Raf signaling in melanoma progression | 1.07E-10 | Tuberin, CBP, AKT1, Raptor, RHEB2, ROCK1, ERK1/2, SRp55, CDK1 (p34), BAD, MEK1/2, ERK2 (MAPK1), AKT(PKB), Mcl-1, NOTCH1 (NICD), ITGB1, PDE5A, B-Raf, ITGB3, FOXM1, p90RSK1, Nicastrin, p90Rsk, ROCK, gamma-Secretase complex, FOXO4, Bcl-2, FOXO3A, Kinase MYT1, RhoE, JNK(MAPK8-10), RKIP, SPRY2, RalA, Bim, MITF, mTOR |
| 12 | Development_PTHR1 in bone and cartilage development | 1.08E-10 | p38alpha (MAPK14), c-Fos, PP1-cat, ERK1/2, MEF2C, DKK1, PTHR1, ATF-4, Beta-catenin, PP2A regulatory, PKA-reg (cAMP-dependent), c-Raf-1, HDAC4, G-protein alpha-q/11, QSK, BAD, GSK3 beta, PLC-beta, Bone sialoprotein, TORC2, Adenylate cyclase, CREB1, MEK1/2, Osteoprotegerin, SOX9, RhoA, G-protein alpha-12 family, MMP-13, PTCH1, MSG1, G-protein alpha-12, MEF2A, PP2A catalytic, PLD2, MEF2D, G-protein alpha-s, PKC, RUNX2, PKC-alpha, Bcl-2, MKP-1, LRP6, Cyclin D1, NURR1, Parathyroid hormone, IGF-1, PKA-cat (cAMP-dependent) |
| 13 | Oxidative stress_ROS-induced cellular signaling | 1.28E-10 | p38alpha (MAPK14), Tuberin, SREBP1 (nuclear), ERK1/2, EGR1, PKA-reg (cAMP-dependent), TXNIP (VDUP1), Cytochrome c, Bax, GSK3 beta, FTL, FTH1, IRP2, MDM2, NF-kB, AKT(PKB), Catalase, NF-kB p50/p65, c-Src, NOTCH1 (NICD), Thioredoxin, Chk2, ACACA, RelA (p65 NF-kB subunit), KEAP1, Cul3/KEAP1/Rbx1 E3 ligase, Adrenomedullin, IRP1, PKA-cat alpha, IL-8, SRX1, Pin1, NIK(MAP3K14), Glutaredoxin 1, NFKBIA, GSTP1, ELAVL1 (HuR), HSF1, NOTCH3 (3ICD), PRKD1, p70 S6 kinase1, GPX1, PKC, PTEN, MEKK1(MAP3K1), HSP27, IKK-beta, DLC1 (Dynein LC8a), c-Abl, Cyclin D1, JNK(MAPK8-10), SAE2, SP1, NRF2, ADAM17, p38 MAPK, APEX, mTOR, NALP3 |
| 14 | Development_Negative regulation of STK3/4 (Hippo) pathway and positive regulation of YAP/TAZ function | 1.34E-10 | EGF, CD44, ASPP1, LARG, ERK1/2, G-protein alpha-q/11, YAP1 (YAp65), LPAR2, MLCP (reg), ARHGEF2, Nephrocystin-4, FRMD4A, STK4, PP1-cat alpha, Itch, Actin cytoskeletal, TAZ, PJA2, PDK (PDPK1), ERK1 (MAPK3), RhoA, G-protein alpha-12 family, PARD3, SIAH2, LPAR1, HIPK2, LIMD1, EGFR, ASPP2, RhoGAP5, WBP-2, MASK, NEDD4, ILK, MOBKL1A, PDZ-RhoGEF, Schwannomin (NF2), Mol1b, JNK(MAPK8-10), Angiomotin (AMOT) |
| 15 | Immune response_IL-3 signaling via JAK/STAT, p38, JNK and NF-kB | 1.35E-10 | DHA2, Cyclin D3, c-Fos, JAK1, ICAM1, Tyk2, XBP1, Granzyme B, H-Ras, SOCS1, ID1, Pim-1, BAD, Cyclin D2, I-kB, Survivin, Cyclin A2, RARalpha, IL3RA, NF-kB, IRE1, AKT(PKB), NF-kB p50/p65, c-Src, c-Myc, Mcl-1, IL-2R alpha chain, C3aR, PKM2, E-selectin, Ephrin-B1, ITGB1, JAK2, PI3K cat class IA, CSF2RB, TRAF6, MEK3(MAP2K3), RXRA, RAR-alpha/RXR-alpha, Bcl-6, MKK7 (MAP2K7), STAT3, IKK-beta, Bcl-2, MKP-1, NOTCH1 precursor, Oncostatin M, STAT5, Cyclin D1, CD40(TNFRSF5), STAT6, p38 MAPK, IL-3 receptor |
| 16 | Ligand-independent activation of Androgen receptor in Prostate Cancer | 1.79E-10 | EGF, FGFR2, GAB1, IRS-1, H-Ras, FGF1, ErbB3, Beta-catenin, PP2A regulatory, c-Raf-1, FRS2, STAT5B, FGFR1, Kallikrein 3 (PSA), Androgen receptor, Tip60, GSK3 beta, N-Ras, MEK2(MAP2K2), MDM2, IGF-1 receptor, ERK2 (MAPK1), AKT(PKB), PDK (PDPK1), c-Myc, ERK1 (MAPK3), JAK2, PI3K cat class IA, B-Raf, NCOA3 (pCIP/SRC3), Tcf(Lef), EGFR, SOS, PP2A catalytic, NCOA2 (GRIP1/TIF2), DDX5, STAT3, c-Abl, Cyclin D1, NCOA1 (SRC1), IGF-1, Frizzled |
| 17 | Cytoskeleton remodeling_Regulation of actin cytoskeleton organization by the kinase effectors of Rho GTPases | 1.98E-10 | WRCH-1, Spectrin, PRK1, Alpha-actinin, Rac2, RhoC, LIMK1, Talin, MLCP (reg), Cdc42 subfamily, ERM proteins, ARPC1B, RhoA-related, Actin cytoskeletal, MLCK, BETA-PIX, RhoA, RhoJ, F-Actin cytoskeleton, Myosin II, Alpha adducin, PAK1, RhoB, PIP5KI, MyHC, LIMK, MRCK, RhoGDI alpha, ROCK, DMPK, Actomyosin, MRCKalpha, PAK, Rac1-related, Vinculin, Rhov, MRLC, TC10 |
| 18 | Mitogenic action of ErbB2 in breast cancer | 2.38E-10 | Tuberin, EGF, RHEB2, c-Fos, H-Ras, TCF7L2 (TCF4), ERK1/2, ErbB3, Beta-catenin, Cyclin G2, c-Raf-1, I-kB, GSK3 beta, FKHR, MEK1/2, MSK1, NF-kB, AKT(PKB), Cullin 3, PDK (PDPK1), c-Src, p27KIP1, c-Myc, PI3K cat class IA, SMAD7, EGFR, JAB1, ErbB4, SOS, PAK1, p70 S6 kinase1, Cyclin E, FOXO3A, HUNK, Cyclin D1, p70 S6 kinase2, mTOR |
| 19 | DNA damage_ATM/ATR regulation of G1/S checkpoint | 5.93E-10 | p38alpha (MAPK14), FBXW7, ERK1/2, PP2A regulatory, CDC25A, PER3, p70 S6 kinases, Chk1, CDK4, PP2A structural, MEK2(MAP2K2), MDM2, B56G, p27KIP1, FBXW11, Chk2, Brca1, SMG1, beta-TrCP, Histone H2AX, PP2A catalytic, BTG2, ATR, ELAVL1 (HuR), PCNA, Cyclin E, FOXO3A, c-Abl, Cyclin A, Cyclin D1, Brca1/Bard1 |
| 20 | Cell adhesion_Tight junctions | 5.93E-10 | Rich1, JAM2, MUPP1, EPB41, MPP5, F-Actin, ARP3, INADL, AF-6, ACTR3, PKC-zeta, Actin, APXL, p114-RhoGEF, Actin cytoskeletal, Tubulin (in microtubules), RhoA, Cingulin, PARD3, Myosin II, DNMBP(TUBA), ROCK, Actomyosin, PDZ-RhoGEF, Tubulin alpha, N-WASP, MRLC, CGNL1, Occludin, ZO-3, Angiomotin (AMOT) |
| 21 | FAK1 signaling in melanoma | 6.59E-10 | ERK1/2, CRK, RhoC, Talin, alpha-V/beta-3 integrin, N-Ras, MEK1/2, Actin cytoskeletal, NF-kB, CAS-L, NF-kB p50/p65, c-Src, RhoA, ITGB1, ARCGAP22, RelA (p65 NF-kB subunit), B-Raf, Syntenin 1, NF-kB1 (p50), ROCK2, ITGB3, alpha-5/beta-1 integrin, SOS, NFKBIA, PLAU (UPA), p130CAS, PKC-alpha, Caveolin-1, ITGA5, WASF2 |
| 22 | The role of UV radiation in melanoma development | 7.19E-10 | ERK1/2, XPF, XRCC3, USF1, Amphiregulin, ADAM9, N-Ras, XPG, NF-kB, ERK2 (MAPK1), AKT(PKB), NF-kB p50/p65, alpha-MSH, MC1R, p16INK4, POMC, B-Raf, TRAF2, EGFR, ASK1 (MAP3K5), MKK7 (MAP2K7), NFKBIA, MEKK1(MAP3K1), ATF-2, JNK(MAPK8-10), ADAM17, p38 MAPK, MITF, XPD |
| 23 | IL-6 signaling in breast cancer cells | 7.46E-10 | IP10, c-Fos, GAB1, ESR1 (nuclear), JAK1, H-Ras, ERK1/2, c-Raf-1, Vimentin, MDR1, Survivin, CYP19, MEK1/2, TWIST1, AKT(PKB), c-Myc, Mcl-1, JAK2, PI3K cat class IA, RelA (p65 NF-kB subunit), NF-kB1 (p50), JAB1, SOS, IL6RA, STAT3, SHP-2, Bcl-2, Fascin, AKT2, Cyclin D1, SNAIL1, Jagged1, Tensin 4, NOTCH3, C/EBPdelta |
| 24 | Cell cycle_Influence of Ras and Rho proteins on G1/S Transition | 7.46E-10 | H-Ras, ERK1/2, c-Raf-1, MLCP (reg), GSK3 beta, Rb protein, GGTase-I, CDK4, Cyclin A2, Tob1, MEK2(MAP2K2), MDM2, AKT(PKB), NF-kB p50/p65, PDK (PDPK1), p27KIP1, c-Myc, MLCK, RhoA, MLK3(MAP3K11), PI3K cat class IA, RelA (p65 NF-kB subunit), ROCK2, alpha-5/beta-1 integrin, PAK1, NFKBIA, CDK6, STAT3, p70 S6 kinase1, ATF-2, Cyclin E, MRLC, RGL2, Cyclin D1, RalA |
| 25 | Development_Negative regulation of WNT/Beta-catenin signaling in the nucleus | 8.92E-10 | TBL1X, AKT1, Calcineurin A (catalytic), BACH1, HBP1, Oct-3/4, VHL, Alpha-1 catenin, TCF7L2 (TCF4), 14-3-3, Jade-1, Beta-catenin, BCL9/B9L, PC1-CTT, TLE, Nitrilase 1, Dsh, KLF4, Menin, NF-AT5, GSK3 beta, Nephrocystin-4, HDAC2, ICAT, P15RS, CtBP, TAB2, PJA2, HIC5, TAK1(MAP3K7), SOX9, LRP5/LRP6, TRRAP, NARF, c-Cbl, Tcf(Lef), TRIM33, GLI-3R, CHD8, SENP2, NLK, RNF43, WNT5A, WNT, FOXO3A, GPX4, CHIBBY, Frizzled, Histone H1, DACT1 |
| 26 | Signal transduction_Calcium-mediated signaling | 9.95E-10 | Calcineurin A (catalytic), c-Fos, ERK1/2, 14-3-3, CaMK I, EGR1, HDAC4, CaMK IV, MUNC13, p47-phox, IP3 receptor, BAD, MLCP (reg), I-kB, PPA5, TORC2, CREB1, NF-kB, Calcitonin receptor, AKT(PKB), RhoA, 14-3-3 epsilon, cPKC (conventional), MARK2, RelA (p65 NF-kB subunit), ASK1 (MAP3K5), MEK3(MAP2K3), CABIN1, Myosin II, MEF2, HDAC5, RhoGDI alpha, ROCK, Calmodulin, PKC, PKC-alpha, ATF-2, CaMKK, IKK-beta, JNK(MAPK8-10), NURR1, p38 MAPK, CaMKK2 |
| 27 | Immune response_IL-3 signaling via ERK and PI3K | 1.04E-09 | Calcineurin A (catalytic), c-Fos, alpha-4/beta-1 integrin, Fc epsilon RI gamma, RPS6, H-Ras, ERK1/2, CrkL, EGR1, c-Raf-1, A-Raf-1, PKC-epsilon, IP3 receptor, BAD, Cyclin D2, Talin, p70 S6 kinases, GSK3 beta, MDM2, GSK3 alpha/beta, CREB1, MEK1/2, IL3RA, E4BP4, AKT(PKB), PDK (PDPK1), p27KIP1, Mcl-1, ITGB1, JAK2, cPKC (conventional), PI3K cat class IA, B-Raf, c-Cbl, CSF2RB, mTORC1, alpha-5/beta-1 integrin, SOS, PAK1, C3G, PDE4, p90Rsk, PI3K cat class IA (p110-alpha), Calmodulin, FLII, SHP-2, Bcl-2, FOXO3A, STAT5, Calcineurin B (regulatory), Bim, PKA-cat (cAMP-dependent), LPCAT2, mTOR, IL-3 receptor |
| 28 | Signal transduction_IGF-1 receptor signaling pathway | 1.06E-09 | SREBP1 (nuclear), GAB1, JAK1, IRS-2, IRS-1, RPS6, H-Ras, ERK1/2, PKC-zeta, c-Raf-1, BAD, Androgen receptor, I-kB, GSK3 beta, FKHR, IGF-2, IGF-1 receptor, MEK1/2, AKT(PKB), PDK (PDPK1), RACK1, JAK2, 14-3-3 epsilon, PI3K cat class IA, RelA (p65 NF-kB subunit), ASK1 (MAP3K5), Cyclin D, SOS, MKK7 (MAP2K7), STAT3, p70 S6 kinase1, SHP-2, Bcl-2, FOXO3A, IGF-1, Bim, mTOR |
| 29 | Development_Negative regulation of WNT/Beta-catenin signaling in the cytoplasm | 1.15E-09 | NOTCH1 receptor, CXXC4, VHL, PP1-cat, Presenilin 1, Alpha-1 catenin, FAF1, Beta-catenin, CYLD, PI3K cat class III (Vps34), CXXC5, DAB2, Dsh, Casein kinase I alpha, YAP1 (YAp65), WWP1, STK4, Itch, GSK3 alpha/beta, G-protein beta/gamma, WDR26, Skp2/TrCP/FBXW, TAZ, RACK1, LRP5/LRP6, Prickle-1, c-Cbl, HIPK2, Tcf(Lef), HUWE1, beta-TrCP, PP2A catalytic, SENP2, RNF185, A20, YAP1/TAZ, CDK6, ELAVL1 (HuR), PEG3, WNT5A, PKC-alpha, WNT, Beclin 1, NEDD4L, Cyclin D1, Frizzled, DACT1 |
| 30 | Apoptosis and survival_Endoplasmic reticulum stress response pathway | 1.31E-09 | p38alpha (MAPK14), GRP78, C/EBP zeta, SOD2, Apaf-1, XBP1, PP1-cat, ATF-4, S2P, GADD34, ATF-6 alpha (90kDa), Cytochrome c, Derlin-3, I-kB, Bax, PP1-cat alpha, IRE1, NF-Y, Calpain 1(mu), NF-kB p50/p65, ATF-6 alpha (50kDa), Derlin-2, EDEM, TRAF2, ASK1 (MAP3K5), MEK3(MAP2K3), S1P, tBid, Bcl-2, IP3R1, JNK(MAPK8-10), DNAJC3, Derlin1, Bim, Bid, ERP5 |
