# Supplementary material for: Prognoses and genomic analyses of proteasome 26S subunit, ATPase (PSMC) family genes in clinical breast cancer
Source: Aging (Albany NY). 2021 Jul 30;13(14):17970. doi: 10.18632/aging.203345 (PMC8351721; doi:10.18632/aging.203345)
Supplement: Supplementary Table 4 [file aging-13-203345-s004.docx]

**Supplementary Table 4. Pathway analysis of genes coexpressed with proteasome 26S subunit, ATPase 2 (PSMC2) from public breast cancer databases using the MetaCore database (with p<0.01 set as the cutoff value).**

| # | Map | *p* Value | Network objects from active data |
| --- | --- | --- | --- |
| 1 | Development_Negative regulation of WNT/Beta-catenin signaling in the cytoplasm | 1.78E-15 | Casein kinase I delta, CXXC4, VHL, DP1, PP1-cat, RIPK4, Presenilin 1, Alpha-1 catenin, Laforin, FAF1, Casein kinase I epsilon, Beta-catenin, CYLD, PI3K cat class III (Vps34), CXXC5, DAB2, Nucleoredoxin, Dsh, YAP1 (YAp65), WWP1, STK4, SIAH1, Ankyrin-G, LATS2, G-protein beta/gamma, Skp2/TrCP/FBXW, TAZ, Axin, RACK1, E-cadherin, LRP5/LRP6, Prickle-1, c-Cbl, HIPK2, STK3, Tcf(Lef), Amer1, beta-TrCP, PR72, JNK1(MAPK8), RNF185, A20, Cul1/Rbx1 E3 ligase, YAP1/TAZ, CDK6, ELAVL1 (HuR), PEG3, Malin, WNT, Beclin 1, NEDD4L, Cyclin D1, NKD2, DACT3, Frizzled, Siah1/SIP/Ebi E3 ligase, DACT1 |
| 2 | Apoptosis and survival_Regulation of apoptosis by mitochondrial proteins | 1.97E-13 | P53AIP1, p38alpha (MAPK14), Bcl-W, PLSCR3, Calcineurin A (catalytic), MPTP complex, Cathepsin H, Granzyme B, ROCK1, ERK1/2, Parkin, MUL1, BOK, BFL1, HRK, PP2C, VDAC 2, Bak, Cytochrome c, OPA1, NIP3, GZMH, Bax, SOD1, PINK1, PP1-cat alpha, AMBRA1, Mitofusin 1, Caspase-2, Bcl-XL, VDAC 1, Calpain 1(mu), Cathepsin D, Cathepsin L, IFI27, Aif, SMCR7, GC1QBP, PKC-delta, Fis1, HXK1, 14-3-3 zeta/delta, MAP1, Pin1, PARL, Metaxin 1, JSAP1, CDK2, NUR77, ATF-2, SLC25A3, TIMM8A, tBid, Bcl-2, Beclin 1, HtrA2, Cyclin A, JNK(MAPK8-10), Mitofusin 2, DNM1L (DRP1), Calcineurin B (regulatory), Smac/Diablo, p38 MAPK, Bim, Bid |
| 3 | Transcription_HIF-1 targets | 1.16E-12 | PDK1, PDGF-B, EG-VEGF, PLGF, Oct-3/4, P4HA2, PFKL, REDD1, SLC9A1, Cyclin G2, ENO1, MSH6, Carbonic anhydrase IX, PGK1, Stanniocalcin 2, NIP3, MDR1, AK3, Adipophilin, PLAUR (uPAR), Alpha-1B adrenergic receptor, DEC1 (Stra13), c-Myc, Epo, CXCR4, LRP1, TERT, PKM2, G3P2, Heme oxygenase 1, TGF-beta 2, DEC2, Ceruloplasmin, ROR-alpha, Adrenomedullin, HIF1A, HXK1, GPI, P4HA1, Angiopoietin 2, Thrombospondin 1, TfR1, Mxi1, GLUT1, Carbonic anhydrase XII, MSH2, ALDOA, NUR77, Transferrin, TGF-beta 3, CX3CR1, Galectin-1, MCT4, LDHA, ABCG2, HGF receptor (Met), Leptin, MMP-9, PAI1 |
| 4 | Cytoskeleton remodeling_Regulation of actin cytoskeleton organization by the kinase effectors of Rho GTPases | 6.94E-12 | WRCH-1, Spectrin, SLC9A1, PRK1, Alpha-actinin, RhoC, LIMK1, Talin, MLCP (reg), Cdc42 subfamily, MSN (moesin), ERM proteins, ARPC1B, RhoA-related, Actin cytoskeletal, MLCK, MLCP (cat), BETA-PIX, RhoA, Citron, RhoJ, F-Actin cytoskeleton, Arp2/3, Myosin II, CPI-17, Alpha adducin, RhoB, PIP5KI, MyHC, LIMK, Rac3, MRCK, RhoGDI alpha, ROCK, DMPK, Actomyosin, Rac1-related, Vinculin, Rhov, MRLC, TC10 |
| 5 | Cell cycle_Chromosome condensation in prometaphase | 7.91E-11 | TOP1, Cyclin B, CAP-G/G2, CAP-D2/D3, AKAP8, CDK1 (p34), Condensin, TOP2, BRRN1, Histone H3, CAP-G, INCENP, CAP-C, Aurora-A, CAP-E, CAP-H/H2, Cyclin A, CNAP1, Aurora-B, Histone H1 |
| 6 | Development_Negative regulation of WNT/Beta-catenin signaling in the nucleus | 1.59E-10 | ZNF703, TBL1X, Casein kinase I delta, Calcineurin A (catalytic), RUNX3, HBP1, Oct-3/4, VHL, PGAM5, Alpha-1 catenin, TCF7L2 (TCF4), 14-3-3, Jade-1, Casein kinase I epsilon, Beta-catenin, PAX7, BCL9/B9L, PC1-CTT, TLE, CBP/P300, Dsh, NF-AT5, Nephrocystin-4, HDAC2, HIC1, LATS2, RANBP3, CtBP, PJA2, HIC5, Axin, SOX9, LRP5/LRP6, TRRAP, NARF, c-Cbl, PKC-delta, Tcf(Lef), CDX2, SOX17, GLI-3R, CHD8, Cul1/Rbx1 E3 ligase, NLK, RNF43, WNT, FOXO3A, CHIBBY, HDAC1, Plakoglobin, Frizzled, Histone H1, DACT1 |
| 7 | Immune response_IFN-alpha/beta signaling via MAPKs | 1.75E-10 | PML, IP10, ISG15, JAK1, Tyk2, TCF7L2 (TCF4), RIG-G, ERK1/2, GCH1, Beta-catenin, Matrilysin (MMP-7), PRMT1, ZNF145, SMAD4, PIAS1, IFN-beta, STAT1, PL scramblase 1, ULK1, IKK-epsilon, Ubiquitin, TAP1 (PSF1), IFN-alpha, ISG54, MEK6(MAP2K6), Axin2, p130, MSK1, RSAD2, FZD7, Lck, PKR, PKC-delta, IRF9, JNK1(MAPK8), MEK4(MAP2K4), MEKK1(MAP3K1), SP5, Filamin B (TABP), FOXO3A, FasR(CD95), Cyclin D1, JNK(MAPK8-10), HDAC1, SMAD3, p38 MAPK |
| 8 | Immune response_IL-4-induced regulators of cell growth, survival, differentiation and metabolism | 1.9E-10 | ATP6V1B2, DHA2, MCM5, IL-4R type I, SOCS1, CDC25A, Cytochrome c, ATP6V0A1, STAT1, CPT-1A, Filaggrin, EGR2 (Krox20), Bax, CISH, CDK4, Bcl-XL, ACADM, FasL(TNFSF6), c-Myc, GATA-3, A-FABP, MMP-13, PLEKHF1, MCM4, IL-4R type II, Cyclin D, CYP2E1, IL4RA, Bcl-6, SK4/IK1, Cathepsin V, CDK2, CDK6, Cyclin E, HSD3B1, Bcl-2, FOXO3A, MCM6, STAT5, Cyclin A, STAT6 |
| 9 | Transcription_Negative regulation of HIF1A function | 2.47E-10 | p14ARF, Casein kinase I delta, MCM5, RUNX3, VHL, COMMD1 (MURR1), FBXW7, SART1, KLF2, VCP, SKP1, UBXD7, Ubiquitin, ING4, EGLN2, MCM7, LAMP2, EAF2, HSP40, MCM2, HSP90, Calpain 1(mu), HSP70, HSPA4, EGLN1, RACK1, MCM3, DEC2, HSC70, HIF1A, ARD1, PSMA7, Sirtuin2, HSP90 beta, Cul1/Rbx1 E3 ligase, AML1 (RUNX1), FHL1 (SLIM1), PTEN, Sirtuin7, SSAT, Elongin C, HIF-prolyl hydroxylase, Proteasome (20S core), PRDX4 |
| 10 | Development_Positive regulation of WNT/Beta-catenin signaling in the nucleus | 2.47E-10 | Casein kinase II, alpha chains, SMYD2, TBL1X, CBP, DP1, Alpha-1 catenin, TCF7L2 (TCF4), FOXP1, Jade-1, Beta-catenin, BCL9/B9L, VCP, TLE, CBP/P300, Dsh, YAP1 (YAp65), HDAC2, UCHL5, PCAF, RUNX, SOX9, TERT, JRK, SOX11, LRP5/LRP6, TWA1, Tcf(Lef), PIAS4, beta-TrCP, FOXM1, Pin1, p300, NCOA2 (GRIP1/TIF2), TDG, Cul1/Rbx1 E3 ligase, NLK, CARF, WNT, FOXO3A, HMGB2, HDAC1, APPL, LRRFIP2, Frizzled |
| 11 | Immune response_HSP60 and HSP70/ TLR signaling pathway | 3.12E-10 | MHC class II, ERK1/2, MD-2, E2N(UBC13), IKK (cat), IL-12 alpha, Ubiquitin, IL-1 beta, I-kB, CD14, MyD88, IKK-alpha, MEK6(MAP2K6), MHC class I, MEK1/2, TPL2(MAP3K8), CD83, NF-kB, HSP60, UEV1A, HSP70, TNF-alpha, CD80, NF-kB1 (p105), IL-8, TRAF6, TIRAP (Mal), TAB3, MEK4(MAP2K4), UBE1, IKK-beta, TAB1, IRAK1/2, JNK(MAPK8-10), CD40(TNFRSF5), p38 MAPK, CD86 |
| 12 | Oxidative stress_ROS-induced cellular signaling | 5.02E-10 | Casein kinase II, alpha chains, p38alpha (MAPK14), Tuberin, SREBP1 (nuclear), ERK1/2, EGR1, IKK (cat), Bak, Cytochrome c, FASN, Carbonic anhydrase IX, IL-1 beta, Bax, FTL, IKK-alpha, FTH1, IRP2, GRP75, NF-kB, Cyclin B1, TNF-alpha, Thioredoxin, Heme oxygenase 1, Chk2, Adrenomedullin, HIF1A, IL-8, SRX1, Pin1, NIK(MAP3K14), Glutaredoxin 1, p300, JNK1(MAPK8), HSPA1A, GSTP1, TfR1, ELAVL1 (HuR), HSF1, NOTCH3 (3ICD), PRKD1, GPX1, PKC, LKB1, PTEN, MEKK1(MAP3K1), HES1, HSP27, IKK-beta, DLC1 (Dynein LC8a), c-Abl, Cyclin D1, JNK(MAPK8-10), HIF-prolyl hydroxylase, HDAC1, SAE2, SP1, NRF2, p38 MAPK, APEX, PAI1 |
| 13 | Immune response_IFN-alpha/beta signaling via PI3K and NF-kB pathways | 6.96E-10 | Tuberin, Cyclin D3, ISG15, JAK1, IRS-2, Tyk2, IRS-1, DHFR, NMI, ERK1/2, IKK (cat), CDC25A, PKC-epsilon, IFN-beta, CDK1 (p34), I-kB, p70 S6 kinases, PI3K reg class IA (p85), Rb protein, CDK4, IKK-alpha, IFN-alpha, p19, ISG54, CREB1, p130, MEK1/2, RSAD2, NF-kB, PDK (PDPK1), p16INK4, p107, c-Myc, PU.1, pRB/E2F4, PKC-delta, I-TAC, PDCD4, p130/E2F4, p107/E2F4, eIF4B, eIF4G1/3, NIK(MAP3K14), CDK2, PCNA, 4E-BP1, GBP1, IFIT1, E2F4, Cyclin E, eIF4A, FOXO3A, MNK2(GPRK7), Cyclin A |
| 14 | Glomerular injury in Lupus Nephritis | 8.69E-10 | GRO-2, PDGF-B, IP10, HMGB1, CCL2, NGAL, H-Ras, C5a, IFN-gamma, ERK1/2, ATF-4, IRF1, PKC-epsilon, IFN-beta, FN14(TNFRSF12A), STAT1, CCL5, IL-1 beta, Bax, GRO-1, ErbB2, MDA-5, IFN-alpha, GM-CSF, MEK1/2, FasL(TNFSF6), NF-kB, RIG-I, PDK (PDPK1), IFI56, TNF-alpha, C5aR, Otubain1, VCAM1, p22-phox, HIF1A, MMP-1, IL-8, TRAF6, TGF-beta, SLC22A17, PDGF-AB, A20, Annexin II, Bcl-2, FasR(CD95), Cyclin D1, JNK(MAPK8-10), MIP-1-alpha, TLR3, p38 MAPK, MMP-9, PDGF-R-beta |
| 15 | DNA damage_p53 activation by DNA damage | 1.14E-09 | P53AIP1, p38alpha (MAPK14), CBP, PML, 14-3-3, PP2A regulatory, P53DINP1a, 14-3-3 theta, Tip60, Chk1, Bax, TTC5 (Strap), PCAF, PP2A structural, MEK6(MAP2K6), Bcl-XL, FBXO31, DBC1, RFWD3, Chk2, Brca1, SMG1, HIPK2, PKC-delta, CABIN1, DDB1, AATF (Che-1), MARKK, p300, DYRK2, MEK4(MAP2K4), ELAVL1 (HuR), MEKK1(MAP3K1), Bcl-2, c-Abl, COP1, JNK(MAPK8-10), p38 MAPK, PP2C gamma |
| 16 | Cell cycle_Cell cycle (generic schema) | 2.16E-09 | Cyclin B, E2F5, DP1, CDC25A, E2F3, CDK1 (p34), Rb protein, E2F2, CDK4, p130, p107, CDC25C, CDC25B, Cyclin D, CDK2, CDK6, E2F4, Cyclin E, Cyclin A |
| 17 | Epigenetic alterations in ovarian cancer | 2.59E-09 | DNMT3B, TIMP3, ESR1 (nuclear), ZIC4, CDC20, HRK, DAB2, WIF1, FGFR1, GLI-2, Bax, ErbB2, HDAC2, CDK4, Vasohibin-1, CARD5, IGF-2, AL1A1, EZH2, SKP2, TWIST1, HSD3B2, p16INK4, E-cadherin, FGFR3, Brca1, Histone H3, DAPK1, SF1, ErbB4, DNMT3A, Thrombospondin 1, Aurora-A, DOK2, CYP11A1, CDK6, GSTP1, Gamma-synuclein, Dlec1, Claudin-4, SSTR1, Aurora-B, HDAC1, BLU, GLI-1, DACT3, MMP-9, Thrombospondin 2 |
| 18 | Role of IGH translocations in multiple myeloma | 3.35E-09 | alpha-E/beta-7 integrin, NCAM1, Cyclin D3, CCR1, Cyclin D2, Rb protein, HDAC2, MafB, CDK4, ITGB7, p130, ITGAE, p107, c-Myc, E-cadherin, FGFR3, Histone H3, Sin3A, IRF4, NOTCH2, CDK6, c-Maf, E2F4, HES1, RhoE, Cyclin D1, HDAC1, MRF-1, Histone H4 |
| 19 | Proteolysis_Putative ubiquitin pathway | 3.67E-09 | FBXW7, UBCH7, Parkin, E2N(UBC13), UBCH8, SKP1, Ubiquitin, UBCH6, UBE2D1, SKP2, UEV1A, HSP70, GPR37, TRAF6, Cul1/Rbx1 E3 ligase, Cullin 1, UBE1, MJD (ataxin-3), RING-box protein 1, Synphilin 1 |
| 20 | Coronavirus disease-19 | 6.21E-09 | GRO-2, MIG, MHC class II, IP10, ISG15, ECSIT, CCL2, Tyk2, NKG2A, IFN-gamma, ERK1/2, TBK1, MSK1/2 (RPS6KA5/4), IKK (cat), VISA, TBKBP1(SINTBAD), Angiotensin II, IFN-beta, STAT1, CCL5, IL-1 beta, I-kB, HDAC2, MyD88, MDA-5, IFN-alpha, CCL8, cPLA2, MEK6(MAP2K6), SRP19, Casein kinase II, CREB1, MHC class I, NF-kB, Eotaxin, RIG-I, ACE2, IL29, TNF-alpha, Cathepsin L, Casein kinase II, beta chain (Phosvitin), JAK2, sIL2RA, IL28B, TRAF3, IL-8, PIP5KIII, AGTR1, eIF4H, SRP54, G3BP1 (hdhVIII), SRP72, CXCL16, TRIM59, Casein kinase II, alpha' chain (CSNK2A2), CCL7, TMPRSS4, NRDP1, TOM70, CD8, CD3, SP1, MIP-1-alpha, TLR3, 4EHP, RAE1, p38 MAPK, PAI1 |
| 21 | Cell cycle_Role of SCF complex in cell cycle regulation | 7.96E-09 | NEDD8, CDC25A, SKP1, CDK1 (p34), Ubiquitin, Chk1, Wee1, CKS1, CDK4, p130, Skp2/TrCP/FBXW, SKP2, Emi1, PLK1, beta-TrCP, Cul1/Rbx1 E3 ligase, CDK2, Cullin 1, UBE1, Cyclin E, Cyclin D1, RING-box protein 1, SMAD3 |
| 22 | Role of activation of WNT signaling in the progression of lung cancer | 8.71E-09 | RUNX3, Oct-3/4, Krm1, TCF7L2 (TCF4), DKK1, FZD8, Beta-catenin, Matrilysin (MMP-7), Dsh, WIF1, ING4, FZD2, Survivin, DKK3, Axin2, EZH2, SKP2, FZD9, c-Myc, FZD7, DVL-2, Axin, CD147, RUVBL1, WNT4, E-cadherin, ROR2, Livin, Tcf(Lef), beta-TrCP, WNT3, ARD1, MKK7 (MAP2K7), JNK1(MAPK8), SFRP5, LKB1, MEKK1(MAP3K1), WNT, FZD5, LRP6, Cyclin D1, HOXB9, p38 MAPK, Frizzled, NOTCH3 |
| 23 | Regulation of degradation of deltaF508-CFTR in CF | 8.99E-09 | NPL4, VCP, HDAC6, Sti1, Ubiquitin, Csp, UBE2D1, HSP90, HSP70, RNF5, SEC61 complex, UFD1, HSC70, Dynein 1, cytoplasmic, light chains, HSP105, Hdj-2, SAE1, SUMO-3, USP19, UBE1, Aha1, SUMO-2, HSP27, UCHL1, AMFR, MJD (ataxin-3), Proteasome (20S core), Derlin1 |
| 24 | NRF2 regulation of oxidative stress response | 9.08E-09 | Casein kinase II, alpha chains, CBP, NRF1, GSHB, CRM1, SMRT, SLC7A11, GSTM3, Ubiquitin, UGT1A1, SOD1, PI3K reg class IA, RARalpha, Actin cytoskeletal, Fyn, GSTA3, MafF, PDK (PDPK1), ERK1 (MAPK3), MEK1(MAP2K1), Casein kinase II, beta chain (Phosvitin), Thioredoxin, Heme oxygenase 1, GCL reg, DJ-1, GSTA2, PRDX1, JNK1(MAPK8), MEK4(MAP2K4), GSTP1, PKC, GSTA1, TXNRD1, NRF2, NQO1 |
| 25 | Cell cycle_Spindle assembly and chromosome separation | 9.33E-09 | DCTN2, Cyclin B, Separase, Nek2A, CDC20, MAD2a, Importin (karyopherin)-alpha, RCC1, Kid, CSE1L, CDK1 (p34), Ubiquitin, TPX2, Ran, Tubulin (in microtubules), Importin (karyopherin)-beta, NUMA1, ZW10, Dynein 1, cytoplasmic, light chains, Aurora-A, Securin, Tubulin alpha, KNSL1, Aurora-B, HEC |
| 26 | Cell cycle_ESR1 regulation of G1/S transition | 9.49E-09 | CRM1, c-Fos, ESR1 (nuclear), ERK1/2, CDC25A, Ubiquitin, Rb protein, CKS1, CDK4, Cyclin A2, p130, Skp2/TrCP/FBXW, SKP2, c-Myc, NCOA3 (pCIP/SRC3), Cul1/Rbx1 E3 ligase, CDK2, CDK6, Cullin 1, E2F4/DP1 complex, CARM1, E2F4, Cyclin E, Cyclin A, Cyclin D1, SP1 |
| 27 | Immune response_IL-3 signaling via JAK/STAT, p38, JNK and NF-kB | 1.78E-08 | MHC class II, DHA2, Cyclin D3, c-Fos, JAK1, Tyk2, XBP1, Granzyme B, H-Ras, SOCS1, IKK (cat), Pim-1, STAT1, Cyclin D2, I-kB, CISH, SRP9, Survivin, Cyclin A2, IKK-alpha, RARalpha, Bcl-XL, NF-kB, IRE1, Cyclin B1, c-Myc, IL-2R alpha chain, PKM2, PU.1, JAK2, C/EBPbeta, Lyn, TRAF6, RXRA, 14-3-3 gamma, RAR-alpha/RXR-alpha, Bcl-6, MKK7 (MAP2K7), JNK1(MAPK8), JAK3, MEK4(MAP2K4), IKK-beta, Bcl-2, KSR1, Oncostatin M, STAT5, Cyclin D1, HDAC1, CD40(TNFRSF5), STAT6, p38 MAPK |
| 28 | DNA damage_ATM/ATR regulation of G2/M checkpoint: cytoplasmic signaling | 2.57E-08 | p38alpha (MAPK14), BORA, PP1-cat, 14-3-3, PP2A regulatory, CDC25A, Nek11, CDK1 (p34), MLCP (reg), TAO2, Chk1, MEK6(MAP2K6), Cyclin B1, PLK1, MLCP (cat), Chk2, Brca1, Histone H3, CDC25C, beta-TrCP, CDC25B, JAB1, 14-3-3 gamma, MARKK, Aurora-A, Cul1/Rbx1 E3 ligase, MEK4(MAP2K4), UBE2C, MEKK1(MAP3K1), FOXO3A, c-Abl, Aurora-B, p38 MAPK |
| 29 | Signal transduction_Calcium-mediated signaling | 2.93E-08 | Calcineurin A (catalytic), c-Fos, ERK1/2, 14-3-3, CaMK I, EGR1, HDAC4, MUNC13, p47-phox, IP3 receptor, MLCP (reg), I-kB, PPA5, MEK6(MAP2K6), CREB1, NF-kB, Calcitonin receptor, MLCP (cat), RhoA, Tiam1, Myocardin, CABIN1, Myosin II, MEF2, HDAC5, p300, RhoGDI alpha, MEK4(MAP2K4), PPCKC, ROCK, Calmodulin, PKC, NUR77, MYH11, ATF-2, CaMKK, IKK-beta, JNK(MAPK8-10), NURR1, p38 MAPK, MMP-9, CaMKK2 |
| 30 | Transcription_Sin3 and NuRD in transcription regulation | 5.17E-08 | TR-alpha, SMRT, SAP130, ARID4A, MBD2, HDAC2, Mi-2 beta, NRSF, RARalpha, Mi-2 alpha, N-CoR, ARID4B, p66beta, PSF, RAR-alpha/TR-alpha, Mi-2, Histone H3, Sin3A, RXRA, RAR-alpha/RXR-alpha, MTA2, RBBP7 (RbAp46), Sin3B, HDAC1, SAP30, NRB54, p66alpha, Histone H4 |
