# Supplementary material for: Prognoses and genomic analyses of proteasome 26S subunit, ATPase (PSMC) family genes in clinical breast cancer
Source: Aging (Albany NY). 2021 Jul 30;13(14):17970. doi: 10.18632/aging.203345 (PMC8351721; doi:10.18632/aging.203345)
Supplement: Supplementary Table 5 [file aging-13-203345-s005.docx]

**Supplementary Table 5. Pathway analysis of genes coexpressed with proteasome 26S subunit, ATPase 3 (PSMC3) from public breast cancer databases using the MetaCore database (with p<0.01 set as the cutoff value).**

| # | Maps | *p* Value | Network Objects from Active Data |
| --- | --- | --- | --- |
| 1 | Cytoskeleton remodeling_Regulation of actin cytoskeleton organization by the kinase effectors of Rho GTPases | 8.41E-17 | WRCH-1, Spectrin, SLC9A1, PRK1, Caldesmon, Alpha-actinin, Rac2, RhoC, LIMK1, Talin, MLCP (reg), GIT1, Cdc42 subfamily, ERM proteins, ARPC1B, RhoA-related, Cortactin, CDC42, Actin cytoskeletal, MLCK, MLCP (cat), BETA-PIX, RhoA, Citron, RhoJ, F-Actin cytoskeleton, Arp2/3, Myosin II, CPI-17, Alpha adducin, RhoB, PIP5KI, MyHC, LIMK, Rac3, MRCK, RhoGDI alpha, ROCK, DMPK, Actomyosin, PAK, Rac1-related, Vinculin, Rhov, MRLC, TC10, Cofilin |
| 2 | Development_Negative regulation of WNT/Beta-catenin signaling in the cytoplasm | 1.5E-15 | Casein kinase I delta, CXXC4, VHL, DP1, PP1-cat, RIPK4, Presenilin 1, Alpha-1 catenin, Laforin, FAF1, Casein kinase I epsilon, Beta-catenin, CYLD, PI3K cat class III (Vps34), DAB2, Nucleoredoxin, Dsh, YAP1 (YAp65), G-protein alpha-13, WWP1, STK4, Itch, Ankyrin-G, LATS2, G-protein beta/gamma, Skp2/TrCP/FBXW, TAZ, Axin, RACK1, Prickle-1, c-Cbl, HIPK2, STK3, Tcf(Lef), HUWE1, Amer1, beta-TrCP, PP2A catalytic, PR72, SENP2, JNK1(MAPK8), RNF185, A20, YAP1/TAZ, MAP1LC3A, CDK6, ELAVL1 (HuR), PEG3, Malin, PKC-alpha, Porf-2, WNT, Beclin 1, NEDD4L, Cyclin D1, NKD2, Frizzled, DACT1 |
| 3 | Apoptosis and survival_Regulation of apoptosis by mitochondrial proteins | 1.29E-14 | p38alpha (MAPK14), Bcl-W, Calcineurin A (catalytic), MPTP complex, Cathepsin H, Granzyme B, ROCK1, ERK1/2, Parkin, BOK, BFL1, HRK, RAD9A, PP2C, VDAC 2, Bik, Bak, Cytochrome c, Caspase-8, OPA1, NIP3, GZMH, Bax, SOD1, RASSF1, PINK1, PP1-cat alpha, AMBRA1, Mitofusin 1, Bcl-XL, VDAC 1, MIDUO, Calpain 1(mu), Mcl-1, Cathepsin D, Cathepsin L, NOR1, IFI27, Aif, GC1QBP, Fis1, MAP1, Pin1, PP2A catalytic, PARL, Metaxin 1, RAD9, CDK2, Bcl-B, Caspase-10, ATF-2, SLC25A3, TIMM8A, tBid, Bcl-2, Beclin 1, HtrA2, Cyclin A, JNK(MAPK8-10), JNK2(MAPK9), Mitofusin 2, DNM1L (DRP1), Calcineurin B (regulatory), Smac/Diablo, p38 MAPK, Bim, Cofilin, Bid |
| 4 | DNA damage_ATM/ATR regulation of G2/M checkpoint: cytoplasmic signaling | 1.14E-12 | p38alpha (MAPK14), BORA, PP1-cat, 14-3-3, PP2A regulatory, CDC25A, Nek11, CDK1 (p34), MLCP (reg), Chk1, MAPKAPK2, B56G, ERK2 (MAPK1), Cyclin B1, PLK1, MLCP (cat), Chk2, Brca1, Histone H3, IPP-2, CDC25C, beta-TrCP, CDC25B, Nucleolysin TIAR, JAB1, MARKK, PP2A catalytic, ATR, Aurora-A, MEK4(MAP2K4), UBE2C, MEKK1(MAP3K1), FOXO3A, c-Abl, Aurora-B, JNK2(MAPK9), p38gamma (MAPK12), p38 MAPK, DCK |
| 5 | Chemotaxis_Lysophosphatidic acid signaling via GPCRs | 3.44E-12 | AKT1, c-Fos, alpha-6/beta-1 integrin, PI3K cat class IA (p110-beta), H-Ras, LARG, ROCK1, ERK1/2, PRK1, ARHGEF1 (p115RhoGEF), PKC-zeta, Beta-catenin, EGR1, CRK, c-Raf-1, G-protein alpha-q/11, PKC-epsilon, TRIP6, IP3 receptor, YAP1 (YAp65), LPAR2, MLCP (reg), LPAR4, PI3K reg class IA (p85), Bax, PLC-beta, HAS2, CREB1, Bcl-XL, MEK1/2, G-protein beta/gamma, CDC42, Actin cytoskeletal, N-CoR, TAZ, MSK1, AKT(PKB), RhoA, Tiam1, cPKC (conventional), G-protein alpha-12 family, PLC-eta 1, LPAR1, Tcf(Lef), EGFR, G-protein alpha-i family, TRAF6, F-Actin cytoskeleton, MKL2, LIMK, E3KARP (NHERF2), MEK4(MAP2K4), 4E-BP1, p130CAS, ROCK, PRKD1, Caspase-7, PAK, p70 S6 kinase1, PDZ-RhoGEF, PKC, Vinculin, ATF-2, Bcl-2, FasR(CD95), SIVA1, Rho GTPase, JNK(MAPK8-10), MKL1, PLC-beta3, ADAM17, p38 MAPK, Cofilin, PREX1 |
| 6 | Development_Positive regulation of STK3/4 (Hippo) pathway and negative regulation of YAP/TAZ function | 5.91E-12 | Willin, Casein kinase I delta, SCRIB, MPP5, Cullin 2, INADL, AMPK beta subunit, Alpha-1 catenin, 14-3-3, Casein kinase I epsilon, AMPK gamma subunit, Beta-catenin, PKA-reg (cAMP-dependent), c-Raf-1, YAP1 (YAp65), ZO-2, RASSF2, STK4, RASSF1, Adenylate cyclase, Itch, CCDC85C, RASSF5, LATS2, Actin cytoskeletal, Skp2/TrCP/FBXW, TAZ, LRR-1, AMOTL1 (Jeap), Axin, RhoA, PKA-cat alpha, STK3, beta-TrCP, MARKK, Alpha-catenin, RhoGDI alpha, MOBKL1A, WW45, MALS-3, Schwannomin (NF2), G-protein alpha-s, LKB1, FasR(CD95), Mol1b, PEZ, Angiomotin (AMOT), PKA-cat (cAMP-dependent) |
| 7 | Development_Negative regulation of WNT/Beta-catenin signaling in the nucleus | 8.91E-12 | ZNF703, TBL1X, Casein kinase I delta, AKT1, Calcineurin A (catalytic), RUNX3, BACH1, HBP1, Oct-3/4, VHL, PGAM5, Alpha-1 catenin, TCF7L2 (TCF4), 14-3-3, Jade-1, Casein kinase I epsilon, Beta-catenin, BCL9/B9L, PC1-CTT, TLE, CBP/P300, Dsh, NF-AT5, Nephrocystin-4, HDAC2, LATS2, P15RS, RANBP3, CtBP, TBLR1, TAB2, PJA2, HIC5, Axin, TAK1(MAP3K7), SOX9, TRRAP, NARF, c-Cbl, Tcf(Lef), CDX2, GLI-3R, CHD8, SENP2, Kaiso, NLK, RNF43, WNT, FOXO3A, GPX4, CHIBBY, HDAC1, Plakoglobin, Frizzled, Histone H1, DACT1 |
| 8 | Neurogenesis_NGF/ TrkA MAPK-mediated signaling | 9.42E-12 | SPHK1, ERK5 (MAPK7), CDK5, c-Fos, APS, Fra-1, H-Ras, ERK1/2, MEF2C, PKC-zeta, EGR1, PP2A regulatory, SGK1, PKA-reg (cAMP-dependent), CRK, c-Raf-1, FRS2, VGF, PKC-epsilon, Ephrin-A receptor 2, IP3 receptor, TY3H, MAPKAPK2, CalDAG-GEFII, N-Ras, SNX26 (TCGAP), MAP2K5 (MEK5), KIDINS220, CREB1, MEK1/2, PLAUR (uPAR), Flotillin-1, MSK1, RGS2, RUSC1 (NESCA), p107, KCTD11, GAB2, MATK, RIN, B-Raf, NF-kB1 (p50), K-RAS, SOS, PP2A catalytic, C3G, SH2B, p90Rsk, p130CAS, NGF, Calmodulin, PKC-lambda/iota, SUR-8, Cyclin D1, RASGRF1, SORBS1, SP1, PVR, JMJD3, RIT, p38 MAPK, PKA-cat (cAMP-dependent), FosB |
| 9 | Development_Positive regulation of WNT/Beta-catenin signaling in the cytoplasm | 1.02E-11 | Casein kinase II, alpha chains, TBL1X, Bcl-9, IRS-2, IRS-1, PP1-cat, RIPK4, Alpha-1 catenin, 14-3-3, Beta-catenin, SMAD4, Dsh, USP47, ZBED3, Beta-arrestin2, YAP1 (YAp65), CDK1 (p34), PPP2R2A, USP25, PR130, IGF-1 receptor, TBLR1, ERK2 (MAPK1), AKT(PKB), RNF146, Axin, GSKIP, ITGB1, HIPK2, Tcf(Lef), TGT, PP2A catalytic, RNF220, DOCK4, COX-2 (PTGS2), Trabid, Tankyrases, BIG2, Miz-1, USP9X, WNT, USP7, Jouberin, JNK(MAPK8-10), SMAD3, NKD2, MITF, PKA-cat (cAMP-dependent), Frizzled, DACT1 |
| 10 | Signal transduction_Calcium-mediated signaling | 1.29E-11 | Calcineurin A (catalytic), c-Fos, ERK1/2, 14-3-3, CaMK I, EGR1, HDAC4, MUNC13, p47-phox, IP3 receptor, MLCP (reg), I-kB, PPA5, CREB1, NF-kB, Calcitonin receptor, AKT(PKB), MLCP (cat), RhoA, Tiam1, cPKC (conventional), Myocardin, MARK2, RelA (p65 NF-kB subunit), Myosin II, MEF2, HDAC5, p300, RhoGDI alpha, MEK4(MAP2K4), COX-2 (PTGS2), PPCKC, PKC-beta, ROCK, Calmodulin, PKC, MYH11, PKC-alpha, ATF-2, MALT1, CaMKK, IKK-beta, JNK(MAPK8-10), Bcl-10, NURR1, p38 MAPK, MMP-9, CaMKK2 |
| 11 | Development_Negative regulation of STK3/4 (Hippo) pathway and positive regulation of YAP/TAZ function | 2.02E-11 | CD44, ASPP1, LARG, ERK1/2, ARHGEF1 (p115RhoGEF), G-protein alpha-q/11, YAP1 (YAp65), ZO-2, LPAR2, MLCP (reg), Nephrocystin-4, FRMD4A, STK4, PP1-cat alpha, Itch, LATS2, Actin cytoskeletal, TAZ, PJA2, ERK1 (MAPK3), MLCP (cat), RhoA, G-protein alpha-12 family, PARD3, Citron, LPAR1, HIPK2, STK3, EGFR, ASPP2, AGTR1, WBP-2, MASK, NEDD4, MOBKL1A, WW45, PDZ-RhoGEF, Schwannomin (NF2), WTIP, Mol1b, JNK(MAPK8-10), PAR1, Angiomotin (AMOT) |
| 12 | Transcription_Negative regulation of HIF1A function | 3.45E-11 | FHL3, p14ARF, Casein kinase I delta, MCM5, RUNX3, VHL, COMMD1 (MURR1), FBXW7, SART1, VCP, SKP1, UBXD7, Ubiquitin, ING4, EGLN2, MCM7, LAMP2, OS-9, EAF2, HSP40, MCM2, HSP90, Calpain 1(mu), HSP70, HSPA4, SAT2, EGLN1, RACK1, MCM3, DEC2, HIF1A, ARD1, PSMA7, Sirtuin2, HSP90 beta, AML1 (RUNX1), FHL1 (SLIM1), PTEN, Sirtuin7, SSAT, Elongin C, HIF-prolyl hydroxylase, Proteasome (20S core), PRDX4, CHIP, CITED4 |
| 13 | Immune response_IFN-alpha/beta signaling via PI3K and NF-kB pathways | 5.03E-11 | AKT1, Cyclin D3, ISG15, JAK1, IRS-2, Tyk2, IRS-1, RPS6, NMI, eIF4E, ERK1/2, c-Raf-1, IKK (cat), CDC25A, IFNAR1, PKC-epsilon, IFN-beta, CDK1 (p34), I-kB, p70 S6 kinases, PI3K reg class IA (p85), Rb protein, CDK4, IKK-alpha, IFN-alpha, p19, CREB1, p130, MEK1/2, NF-kB, AKT(PKB), p16INK4, p107, PU.1, SLFN5, pRB/E2F4, PI3K cat class IA, RelA (p65 NF-kB subunit), TRAF2, I-TAC, PDCD4, p130/E2F4, p107/E2F4, eIF4B, p90RSK1, eIF4G1/3, CDK2, PCNA, 4E-BP1, GBP1, E2F4, PKC-alpha, Cyclin E, eIF4A, FOXO3A, p15, Cyclin A |
| 14 | Oxidative stress_ROS-induced cellular signaling | 5.19E-11 | Casein kinase II, alpha chains, p38alpha (MAPK14), SREBP1 (nuclear), ERK1/2, EGR1, PKA-reg (cAMP-dependent), IKK (cat), Bak, Cytochrome c, FASN, Carbonic anhydrase IX, Bax, FTL, IKK-alpha, FTH1, IRP2, GRP75, NF-kB, AKT(PKB), NF-kB p50/p65, Cyclin B1, TNF-alpha, Thioredoxin, Heme oxygenase 1, Chk2, ACACA, RelA (p65 NF-kB subunit), Adrenomedullin, PKA-cat alpha, HIF1A, Pin1, p300, JNK1(MAPK8), NFKBIA, HSPA1A, GSTP1, COX-2 (PTGS2), ELAVL1 (HuR), HSF1, PKC-beta, PRKD1, p70 S6 kinase1, GPX1, PKC, LKB1, PTEN, MEKK1(MAP3K1), HES1, HSP27, IKK-beta, DLC1 (Dynein LC8a), c-Abl, Cyclin D1, JNK(MAPK8-10), HIF-prolyl hydroxylase, HDAC1, SAE2, SP1, NRF2, ADAM17, p38 MAPK, APEX, PAI1 |
| 15 | Immune response_IL-1 signaling pathway | 1.4E-10 | SPHK1, p38alpha (MAPK14), IP10, ECSIT, ICAM1, CD44, RANKL(TNFSF11), ERK1/2, PKC-zeta, EGR1, IKK (cat), IRF1, IFN-beta, MEK4/7, CCL5, I-kB, PI3K reg class IA (p85), MAPKAPK2, GRO-1, GM-CSF, IL-1RI, MEK1/2, TPL2(MAP3K8), Collagen II, NF-kB, AKT(PKB), NF-kB p50/p65, MYLK1, TAK1(MAP3K7), TNF-alpha, c-IAP2, PI3K cat class IA, RelA (p65 NF-kB subunit), MMP-13, MCPIP, NF-kB1 (p105), NF-kB1 (p50), MMP-1, TRAF6, IL1RAP, MEK4(MAP2K4), CCL7, COX-2 (PTGS2), KHSRP, RUNX2, MEKK1(MAP3K1), MAP3K3, HSP27, IRAK1, JNK(MAPK8-10), MMP-9 |
| 16 | Inhibition of remyelination in multiple sclerosis: regulation of cytoskeleton proteins | 1.59E-10 | CDK5, alpha-V/beta-1 integrin, chTOG, HDAC6, KLHL2, LIMK1, Fibronectin, MLCP (reg), PCBP-1, CDC42, WASP, Actin cytoskeletal, Fyn, Tau (MAPT), TNF-alpha, Tubulin (in microtubules), MLCP (cat), Tubulin beta, RhoA, Stathmin, Arp2/3, Myosin II, TGF-beta, Sirtuin2, hnRNP A2, Myelin basic protein, Tubulin alpha, PKC-alpha, MRLC, Tubulin beta 4, MELC, WASF2, Cofilin |
| 17 | Glomerular injury in Lupus Nephritis | 2.37E-10 | OX40L(TNFSF4), GRO-2, IP10, ICAM1, HMGB1, NGAL, H-Ras, C5a, IFN-gamma, ERK1/2, ATF-4, c-Raf-1, IRF1, Fibronectin, PKC-epsilon, IFN-beta, FN14(TNFRSF12A), CCL5, Bax, GRO-1, ErbB2, IFN-alpha, HAS2, GM-CSF, RIPK1, MEK1/2, NF-kB, AKT(PKB), NF-kB p50/p65, RIG-I, TNF-alpha, C5aR, Otubain1, RelA (p65 NF-kB subunit), p22-phox, HIF1A, MMP-1, TRAF6, TGF-beta, SLC22A17, A20, PKC-beta2, CX3CL1, PKC-beta1, PKC-alpha, Annexin II, Bcl-2, FasR(CD95), Cyclin D1, JNK(MAPK8-10), MIP-1-alpha, TLR3, p38 MAPK, MMP-9, PDGF-R-beta |
| 18 | Apoptosis and survival_Ubiquitination and phosphorylation in TNF-alpha-induced NF-kB signaling | 3.78E-10 | SPHK1, E2N(UBC13), TRADD, PKC-epsilon, Ubiquitin, c-IAP1, UBE2D3, RIPK1, Sharpin, UBE2D1, TAB2, NF-kB p50/p65, UEV1A, TNF-R1, TAK1(MAP3K7), TNF-alpha, c-IAP2, RelA (p65 NF-kB subunit), NF-kB1 (p105), NF-kB1 (p50), TRAF2, NFKBIA, Zibra, MAP3K3, RBCK1, IKK-beta, SODD, TAB1, LUBAC E3 ligase, ADAM17 |
| 19 | Apoptosis and survival_Role of PKR in stress-induced apoptosis | 3.84E-10 | C/EBP zeta, JAK1, Tyk2, IFN-gamma, eIF4E, ERK1/2, ATF-4, PP2A regulatory, IKK (cat), IRF1, Caspase-8, IFN-beta, I-kB, IKK-alpha, IFN-alpha, NFAT-90, PPP2R5A, NFKBIB, eIF2S1, NF-kB, MSK2, TAB2, NF-kB p50/p65, TNF-R1, TAK1(MAP3K7), TNF-alpha, TRAF2, TRAF6, PP2A catalytic, NFKBIA, 4E-BP1, Caspase-7, TARBP2, IKK-beta, FasR(CD95), IRF3, TLR3 |
| 20 | Immune response_B cell antigen receptor (BCR) pathway | 5.2E-10 | Calcineurin A (catalytic), NCK1, c-Fos, alpha-4/beta-1 integrin, ICAM1, H-Ras, ERK1/2, EGR1, c-Raf-1, Fibronectin, CD19, IP3 receptor, Cyclin D2, PI3K reg class IA (p85), ORAI1, Rb protein, CD79A, CDK4, IKK-alpha, CalDAG-GEFII, N-Ras, VAV-2, MEK2(MAP2K2), Bcl-XL, MEK1/2, CDC42, WASP, Actin cytoskeletal, NF-kB, AKT(PKB), NF-kB p50/p65, TAK1(MAP3K7), MEKK4(MAP3K4), RelA (p65 NF-kB subunit), LRRK1, B-Raf, NF-kB1 (p50), K-RAS, ETS1, PIP5KIII, Arp2/3, CD79 complex, PP2A catalytic, SOS1, PIP5KI, PKC-beta2, NFKBIA, CDK6, HPK1(MAP4K1), PKC-beta, p70 S6 kinase1, Calmodulin, CalDAG-GEFIII, CKLFSF7, MEKK1(MAP3K1), ATF-2, MALT1, IKK-beta, CD79B, Bcl-10, Calcineurin B (regulatory), p38 MAPK |
| 21 | Apoptosis and survival_IL-17-induced CIKS-dependent MAPK signaling pathways | 6.77E-10 | ERK1/2, E2N(UBC13), IL-17 receptor, IKK-epsilon, Ubiquitin, IL-17RC, IKK-alpha, MEK1/2, TPL2(MAP3K8), TAB2, NF-kB p50/p65, UEV1A, TAK1(MAP3K7), NF-kB1 (p105), TRAF2, C/EBPbeta, TRAF6, ABIN-2, TRAF5, MEK4(MAP2K4), UBE1, MAP3K3, IKK-beta, TAB1, JNK(MAPK8-10), p38 MAPK |
| 22 | DNA damage_ATM activation by DNA damage | 6.97E-10 | Casein kinase II, alpha chains, p14ARF, CDK5, BRAT1, OBFC2B, Aven, PP2A regulatory, p90RSK2(RPS6KA3), E2N(UBC13), Mob2 (HCCA2), PPP2R3A, Tip60, HMG14, HDAC2, SOSSC, MYST1, Itch, Casein kinase II, alpha chain (CSNK2A1), Casein kinase II, RNF8, ASCIZ, RecQL4, CHFR, HSP90, Calpain 1(mu), NDR1 (STK38), RCAD, Brca1, Histone H3, EGFR, RAD17, INTS3, Histone H2B, Histone H2AX, PP2A catalytic, HSP90 beta, p18, Pellino 1, DMAP1, NK31, FOXO3A, c-Abl, TELO2, HDAC1, Histone H4 |
| 23 | Transport_Clathrin-coated vesicle cycle | 6.97E-10 | NSF, Myosin I, Rabaptin-5, Rab11-FIP1, VTI1B, PICALM, PI3K cat class III (Vps34), DAB2, Dynamin-2, Actin, RAB9P40, RABGEF1, Epsin 1, Syntaxin 5, VPS45A, TIP47, Actin cytoskeletal, HIP12, Optineurin, EEA1, Syntaxin 16, RABGDIA, Clathrin heavy chain, VAMP2, Syntaxin 6, PI3K reg class III (p150), AP complex 1, VAMP7, PIP5KIII, GOS-28, VAMP4, ARF1, Syntaxin 8, Syntaxin 7, GS15, Rab-5A, Rabenosyn-5, Myosin VI, SAR1, Rab-11A, Endophilin B1, RILP (Rab interacting lysosomal protein), Clathrin, Myosin Vb, Rip11 |
| 24 | Development_Positive regulation of WNT/Beta-catenin signaling in the nucleus | 8.09E-10 | Casein kinase II, alpha chains, SMYD2, TBL1X, CBP, DP1, Alpha-1 catenin, TCF7L2 (TCF4), FOXP1, Jade-1, Beta-catenin, BCL9/B9L, VCP, TLE, ZIP-kinase, CBP/P300, Dsh, YAP1 (YAp65), HDAC2, UCHL5, WIP1, PCAF, RUNX, TBLR1, ERK2 (MAPK1), SOX9, TERT, JRK, SOX11, TWA1, Tcf(Lef), beta-TrCP, FOXM1, Pin1, p300, NCOA2 (GRIP1/TIF2), TDG, NLK, CARF, WNT, FOXO3A, HMGB2, HDAC1, APPL, Frizzled |
| 25 | Development_The role of GDNF ligand family/ RET receptor in cell survival, growth and proliferation | 8.95E-10 | p38alpha (MAPK14), NCK1, c-Fos, GAB1, IRS-1, H-Ras, ATF-1, ERK1/2, EGR1, p90RSK2(RPS6KA3), CRK, c-Raf-1, IKK (cat), FRS2, LIMK1, IP3 receptor, PI3K reg class IA (p85), CREM (activators), Cyclin A2, IKK-alpha, N-Ras, GFRalpha1, VAV-2, CREB1, Bcl-XL, MEK1/2, CDC42, NF-kB, AKT(PKB), RET, GAB2, RhoA, ITGB1, PI3K cat class IA, B-Raf, HIF1A, PSPN, F-Actin cytoskeleton, SOS, C3G, JNK1(MAPK8), NFKBIA, MEK4(MAP2K4), CDK2, GDNF, ROCK, Calmodulin, MEKK1(MAP3K1), IKK-beta, Bcl-2, Cyclin D1, JNK2(MAPK9), ARTN, Cofilin |
| 26 | TNF-alpha and IL-1 beta-mediated regulation of contraction and secretion of inflammatory factors in normal and asthmatic airway smooth muscle | 1.08E-09 | GRO-2, p38alpha (MAPK14), c-Fos, gp91-phox, ERK1/2, HDAC4, IKK (cat), p47-phox, CCL5, PI3K reg class IA (p85), GRO-1, PLC-beta, PCAF, GM-CSF, Ca-ATPase2, IL-1RI, PA24A, JNK3(MAPK10), NF-kB, Eotaxin, AKT(PKB), NF-kB p50/p65, TNF-R1, TNF-alpha, RhoA, RelA (p65 NF-kB subunit), Histone H3, Cytochrome b-558, EGFR, p300, PKC-beta2, NFKBIA, CCL7, COX-2 (PTGS2), Calmodulin, PKC-alpha, JNK(MAPK8-10), PLA2, p38 MAPK, MMP-9, Histone H4, PDGF-R-beta |
| 27 | Cytoskeleton remodeling_Regulation of actin cytoskeleton nucleation and polymerization by Rho GTPases | 1.12E-09 | RhoF (Rif), BAIAP2, FMNL3, FMNL1, Rac2, mDIA2(DIAPH3), RhoC, CYFIP1, RhoD, DRF, MENA, Profilin, CYFIP2, Cdc42 subfamily, RhoA-related, CDC42, WASP, Actin cytoskeletal, FHOD1, RhoA, F-Actin cytoskeleton, Arp2/3, IQGAP1, WASF subunit, RhoB, PIP5KI, Rac3, Rac1-related, N-WASP, FMNL2, TC10, WASF2, FNBP1L |
| 28 | Apoptosis and survival_BAD phosphorylation | 1.13E-09 | Calcineurin A (catalytic), IRS-1, H-Ras, ERK1/2, 14-3-3, PKA-reg (cAMP-dependent), c-Raf-1, PP2C, Cytochrome c, CDK1 (p34), Bax, PI3K reg class IA, PP1-cat alpha, MEK2(MAP2K2), Bcl-XL, IGF-1 receptor, G-protein beta/gamma, AKT(PKB), PI3K cat class IA, EGFR, Adenylate cyclase type I, SOS, PP2A catalytic, JNK1(MAPK8), p90Rsk, p70 S6 kinase1, G-protein alpha-s, Bcl-2, Beclin 1, p70 S6 kinase2, PKA-cat (cAMP-dependent) |
| 29 | Cell adhesion_Tight junctions | 1.14E-09 | Rich1, MUPP1, EPB41, MPP5, F-Actin, ARP3, INADL, AF-6, PKC-zeta, Actin, ZO-2, APXL, JAM3, Myosin VIIA, Cortactin, CDC42, Actin cytoskeletal, Tubulin (in microtubules), RhoA, Cingulin, PARD3, Arp2/3, Myosin II, ROCK, Actomyosin, PDZ-RhoGEF, Tubulin alpha, PKC-lambda/iota, N-WASP, MRLC, CGNL1, Angiomotin (AMOT) |
| 30 | Immune response_IL-3 signaling via JAK/STAT, p38, JNK and NF-kB | 1.55E-09 | MHC class II, DHA2, Cyclin D3, c-Fos, JAK1, ICAM1, Tyk2, XBP1, Granzyme B, H-Ras, SOCS1, IKK (cat), Fibronectin, Pim-1, Cyclin D2, I-kB, CISH, Survivin, Cyclin A2, IKK-alpha, Bcl-XL, NF-kB, IRE1, AKT(PKB), NF-kB p50/p65, Cyclin B1, Mcl-1, IL-2R alpha chain, PKM2, E-selectin, Ephrin-B1, PU.1, ITGB1, JAK2, PI3K cat class IA, C/EBPbeta, CSF2RB, TRAF6, Bcl-6, MKK7 (MAP2K7), JNK1(MAPK8), JAK3, MEK4(MAP2K4), IKK-beta, Bcl-2, KSR1, Oncostatin M, TACI(TNFRSF13B), STAT5, Cyclin D1, HDAC1, CD40(TNFRSF5), STAT6, p38 MAPK |
