# Supplementary material for: Prognoses and genomic analyses of proteasome 26S subunit, ATPase (PSMC) family genes in clinical breast cancer
Source: Aging (Albany NY). 2021 Jul 30;13(14):17970. doi: 10.18632/aging.203345 (PMC8351721; doi:10.18632/aging.203345)
Supplement: Supplementary Table 6 [file aging-13-203345-s006.docx]

**Supplementary Table 6. Pathway analysis of genes coexpressed with proteasome 26S subunit, ATPase 4 (PSMC4) from public breast cancer databases using the MetaCore database (with p<0.01 set as the cutoff value).**

| # | Map | *p* Value | Network objects from active data |
| --- | --- | --- | --- |
| 1 | Chemotaxis_Lysophosphatidic acid signaling via GPCRs | 5.63E-15 | AKT1, c-Fos, alpha-6/beta-1 integrin, PI3K cat class IA (p110-beta), H-Ras, LARG, ROCK1, ERK1/2, PRK1, ARHGEF1 (p115RhoGEF), PKC-zeta, Beta-catenin, EGR1, CRK, G-protein alpha-q/11, Rac1, IP3 receptor, LPAR2, LPAR4, PI3K reg class IA (p85), GSK3 beta, PLC-beta, FKHR, HAS2, LPAR6, alpha-V/beta-3 integrin, Cyr61, CREB1, Bcl-XL, MEK1/2, G-protein beta/gamma, Actin cytoskeletal, N-CoR, TAZ, MSK1, AKT(PKB), PDK (PDPK1), c-Src, PLC-epsilon, RhoA, E-cadherin, cPKC (conventional), G-protein alpha-12 family, LPAR1, Tcf(Lef), G-protein alpha-i family, TRAF6, F-Actin cytoskeleton, MKL2, LIMK, PLD2, CD36, G-protein gamma 12, 4E-BP1, p130CAS, ROCK, PRKD1, Caspase-7, PAK, PDZ-RhoGEF, PKC, Vinculin, ATF-2, PLC-delta 1, Bcl-2, FasR(CD95), SIVA1, Rho GTPase, JNK(MAPK8-10), CTGF, ADAM17, p38 MAPK, Elk-1, Cofilin, mTOR, PREX1 |
| 2 | Signal transduction_CXCR4 signaling via MAPKs cascades | 1.27E-14 | c-Fos, IL-10, IRS-1, ERK1/2, EGR1, p90RSK2(RPS6KA3), IKK (cat), G-protein alpha-i2, Rac1, Beta-arrestin2, G-protein alpha-13, BAD, Ubiquitin, ZAP70, N-Ras, CREB1, IL-6, Cortactin, MEK1/2, G-protein beta/gamma, c-Src, SDF-1, CXCR4, RhoA, RelA (p65 NF-kB subunit), NF-kB1 (p50), G-protein alpha-i family, NFKBIA, CD69, ROCK, PAK, MEKK1(MAP3K1), CD3, CD3 zeta, ACKR3, JNK(MAPK8-10), SMAD3, CTGF, p38 MAPK, Elk-1, PREX1 |
| 3 | Signal transduction_Angiotensin II/AGTR1 signaling via Notch, Beta-catenin and NF-kB pathways | 1.98E-14 | COL1A1, CBP, NOTCH1 receptor, CCL2, TCF7L2 (TCF4), ROCK1, ERK1/2, Beta-catenin, IKK (cat), TRPC6, Fibronectin, Rac1, I-kB, GSK3 beta, NF-kB2 (p100), IKK-alpha, MMP-2, Axin2, IL-6, IGF-1 receptor, NF-kB, AKT(PKB), NF-kB p50/p65, RBP-J kappa (CBF1), PDK (PDPK1), TAK1(MAP3K7), NOTCH1 (NICD), RhoA, HEY1, E-cadherin, RelA (p65 NF-kB subunit), NF-kB2 (p52), TRAF6, p300, NOTCH3 (3ICD), PRKD1, gamma-Secretase complex, PKC, HES1, WISP1, IKK-beta, Connexin 43, HEY2, Cyclin D1, NCOA1 (SRC1), CTGF, NOTCH1 (NEXT), ADAM17, p38 MAPK, PKA-cat (cAMP-dependent), mTOR, NOTCH3 |
| 4 | Oxidative stress_ROS-induced cellular signaling | 2.92E-14 | p38alpha (MAPK14), Tuberin, SREBP1 (nuclear), ERK1/2, EGR1, PKA-reg (cAMP-dependent), IKK (cat), Bak, Cytochrome c, FASN, E2I, GSK3 beta, FTL, IKK-alpha, FTH1, IRP2, GRP75, MDM2, IL-6, NF-kB, AKT(PKB), Catalase, NF-kB p50/p65, c-Src, NOTCH1 (NICD), Thioredoxin, Heme oxygenase 1, Chk2, ACACA, RelA (p65 NF-kB subunit), KEAP1, Cul3/KEAP1/Rbx1 E3 ligase, Adrenomedullin, HIF1A, SRX1, AMPK alpha subunit, Pin1, Glutaredoxin 1, p300, NFKBIA, HSPA1A, ELAVL1 (HuR), HSF1, NOTCH3 (3ICD), PRKD1, Sirtuin1, GPX1, PKC, LKB1, PTEN, MEKK1(MAP3K1), HES1, IKK-beta, DLC1 (Dynein LC8a), c-Abl, Cyclin D1, JNK(MAPK8-10), SAE2, SP1, NRF2, ADAM17, p38 MAPK, APEX, PAI1, mTOR, NALP3 |
| 5 | Cytoskeleton remodeling_Regulation of actin cytoskeleton organization by the kinase effectors of Rho GTPases | 3.98E-14 | WRCH-1, Spectrin, SLC9A1, PRK1, Caldesmon, Alpha-actinin, Rac2, RhoC, LIMK1, Rac1, Talin, GIT1, Cdc42 subfamily, ERM proteins, ARPC1B, RhoA-related, Cortactin, Actin cytoskeletal, MLCK, BETA-PIX, RhoA, RhoJ, Destrin, F-Actin cytoskeleton, Myosin II, CPI-17, Alpha adducin, RhoB, PIP5KI, MyHC, LIMK, MRCK, ROCK, DMPK, Actomyosin, MRCKalpha, PAK, Rac1-related, Vinculin, Rhov, MRLC, TC10, Cofilin |
| 6 | Development_Positive regulation of WNT/Beta-catenin signaling in the cytoplasm | 1.18E-13 | TBL1X, Bcl-9, EGF, BIG1, GRB2, IRS-2, IRS-1, PP1-cat, RIPK4, Alpha-1 catenin, 14-3-3, Beta-catenin, SMAD4, Dsh, USP47, ZBED3, Rac1, Beta-arrestin2, CDK1 (p34), PPP2R2A, GSK3 alpha/beta, IGF-1 receptor, TBLR1, AKT(PKB), RNF146, Axin, GSKIP, HECTD1, ITGB1, HIPK2, Tcf(Lef), HSP105, PKA-reg type II (cAMP-dependent), PP2A catalytic, RNF220, DOCK4, Trabid, Tankyrases, BIG2, Miz-1, USP9X, WNT, USP7, Jouberin, PP2C alpha, JNK(MAPK8-10), SMAD3, MITF, PKA-cat (cAMP-dependent), Frizzled, DACT1 |
| 7 | Transport_Clathrin-coated vesicle cycle | 4.06E-13 | NSF, AP180, Myosin I, Rabaptin-5, Rab-8, GCC2, Eps15, AP complex 2, PI3K cat class III (Vps34), DAB2, Dynamin-2, Actin, YKT6, RABGEF1, Rab-7, Syntaxin 5, Syntaxin 12, VPS45A, BIN1 (Amphiphysin II), TIP47, GDI2, Actin cytoskeletal, HIP12, Optineurin, EEA1, RABGDIA, VAMP2, PI3K reg class III (p150), VAMP8, HIP1, PIP5KIII, GOS-28, VAMP4, ARF1, Syntaxin 8, Syntaxin 7, Rab-5A, Rabenosyn-5, Myosin VI, SAR1, Rab-11A, Endophilin B1, RILP (Rab interacting lysosomal protein), Rab-9, Rab11-FIP2, PREB, Myosin Vb, Rip11 |
| 8 | Immune response_IFN-alpha/beta signaling via MAPKs | 1.97E-12 | PML, IP10, ISG15, JAK1, Tyk2, TCF7L2 (TCF4), RIG-G, ERK1/2, GCH1, Beta-catenin, PRMT1, ZNF145, SMAD4, PIAS1, IFNAR1, STAT1, Rac1, PL scramblase 1, ULK1, IKK-epsilon, Ubiquitin, TAP1 (PSF1), MAPKAPK2, ZAP70, Axin2, p130, MSK1, RSAD2, AKT(PKB), p27KIP1, HIP-2, Lck, CD45, PKR, SMAD7, MEK3(MAP2K3), IRF9, Ku80, MAPKAPK3, MEKK1(MAP3K1), Filamin B (TABP), FOXO3A, FasR(CD95), Cyclin D1, JNK(MAPK8-10), SMAD3, p38 MAPK, mTOR |
| 9 | Development_Negative regulation of WNT/Beta-catenin signaling in the cytoplasm | 1.99E-12 | Casein kinase I delta, NOTCH1 receptor, VHL, PP1-cat, RIPK4, Presenilin 1, Alpha-1 catenin, FAF1, Beta-catenin, CYLD, PI3K cat class III (Vps34), DAB2, Nucleoredoxin, Dsh, Casein kinase I alpha, Rac1, G-protein alpha-13, STK4, Itch, GSK3 alpha/beta, Ankyrin-G, G-protein beta/gamma, Skp2/TrCP/FBXW, TAZ, Axin, KCTD1, RACK1, KLHL12, E-cadherin, HECTD1, Prickle-1, HIPK2, Tcf(Lef), HUWE1, beta-TrCP, PP2A catalytic, SENP2, RNF185, YAP1/TAZ, MAP1LC3A, CDK6, ELAVL1 (HuR), PEG3, PKC-alpha, Porf-2, WNT, Beclin 1, NEDD4L, Cyclin D1, DACT3, Frizzled, DACT1 |
| 10 | Signal transduction_IGF-1 receptor signaling pathway | 2.15E-12 | SREBP1 (nuclear), GRB2, GAB1, JAK1, IRS-2, IRS-1, H-Ras, eIF4E, ERK1/2, PKC-zeta, IKK (cat), FASN, ACSA, BAD, Androgen receptor, I-kB, PI3K reg class IA (p85), GSK3 beta, FKHR, IGF-2, Bcl-XL, IGF-1 receptor, MEK1/2, AKT(PKB), PDK (PDPK1), RACK1, 14-3-3 epsilon, PI3K cat class IA, RelA (p65 NF-kB subunit), ASK1 (MAP3K5), Cyclin D, SOS, MKK7 (MAP2K7), STAT3, 4E-BP1, SHP-2, Bcl-2, FOXO3A, MNK2(GPRK7), Bim, mTOR |
| 11 | HGF signaling in melanoma | 6.33E-12 | GAB1, CD44, ERK1/2, Beta-catenin, EGR1, Fibronectin, PI3K reg class IA (p85), GSK3 beta, N-Ras, Bcl-XL, AKT(PKB), alpha-MSH, MC1R, HGF, E-cadherin, PI3K cat class IA, Desmoglein 1, NF-kB2 (p52), HIF1A, C/EBPbeta, RXRA, PKC-alpha, ATF-2, SLUG, Cyclin D1, JNK(MAPK8-10), Plakoglobin, HGF receptor (Met), p38 MAPK, Elk-1, MITF |
| 12 | Aberrant B-Raf signaling in melanoma progression | 6.38E-12 | Rictor, Tuberin, CBP, AKT1, RHEB2, ROCK1, ERK1/2, SRp55, CDK1 (p34), BAD, MAP2, MEK1/2, AKT(PKB), Mcl-1, NOTCH1 (NICD), ITGB1, PDE5A, ITGB3, AMPK alpha subunit, ITGA6, Nicastrin, BMF, p90Rsk, ROCK, gamma-Secretase complex, FOXO4, LKB1, HES1, Bcl-2, FOXO3A, Kinase MYT1, RhoE, Aurora-B, JNK(MAPK8-10), RKIP, SPRY2, Bim, MITF, mTOR |
| 13 | Apoptosis and survival_Regulation of apoptosis by mitochondrial proteins | 1.1E-11 | P53AIP1, p38alpha (MAPK14), NIX, Bcl-W, PLSCR3, Calcineurin A (catalytic), MPTP complex, Apaf-1, ROCK1, ERK1/2, BFL1, RAD9A, PP2C, VDAC 2, Bak, Cytochrome c, Caspase-8, OPA1, Endonuclease G, BAD, GZMH, OMA1, SOD1, PP1-cat alpha, AMBRA1, Mitofusin 1, Bcl-XL, MTCH2, VDAC 1, MIDUO, Calpain 1(mu), Mcl-1, Cathepsin D, Cathepsin L, NOR1, IFI27, GC1QBP, Fis1, LETM1, MAP1, Pin1, PP2A catalytic, PARL, Metaxin 1, BMF, RAD9, Cathepsin B, CDK2, ATF-2, SLC25A3, Bcl-2, Beclin 1, HtrA2, Cyclin A, JNK(MAPK8-10), JNK2(MAPK9), Mitofusin 2, Smac/Diablo, p38 MAPK, Bim, Cofilin |
| 14 | IGF signaling in HCC | 1.89E-11 | NOTCH1 receptor, GRB2, c-Fos, IRS-2, IRS-1, H-Ras, ERK1/2, EGR1, PI3K reg class IA (p85), Amphiregulin, GSK3 beta, IGF-2, MEK2(MAP2K2), MMP-2, IGF-1 receptor, TGF-alpha, AKT(PKB), PDK (PDPK1), HGF, Junctin, IRS-4, PI3K cat class IA, HIF1A, C/EBPbeta, SOS, GSTA2, IGF-2 receptor, TIMP1, PKC, HES1, FOXO3A, RKIP, SP1, HGF receptor (Met), Jagged1, ADAM17, Elk-1 |
| 15 | Cell adhesion_PLAU signaling | 2.07E-11 | LAMC2, GRB2, JAK1, Tyk2, alpha-V/beta-1 integrin, H-Ras, ERK1/2, VEGFR-2, STAT5B, STAT1, Rac1, IKK-alpha, c-IAP1, alpha-V/beta-3 integrin, PLAUR (uPAR), NF-kB, AKT(PKB), MYLK1, c-Src, MLCK, C5aR, Casein kinase II, beta chain (Phosvitin), FPRL1, RhoA, alpha-3/beta-1 integrin, c-IAP2, PI3K cat class IA, PI3K reg class IA (p85-alpha), NF-kB2 (p52), G-protein alpha-i family, F-Actin cytoskeleton, G-protein alpha-i3, SOS, sUPAR, alpha-V/beta-5 integrin, PLAU (UPA), STAT3, p130CAS, ROCK, Nucleolin, SHP-2, MRLC, PDGF-R-beta |
| 16 | Development_The role of GDNF ligand family/ RET receptor in cell survival, growth and proliferation | 5.9E-11 | p38alpha (MAPK14), NCK1, GRB2, c-Fos, GAB1, IRS-1, H-Ras, ATF-1, ERK1/2, EGR1, p90RSK2(RPS6KA3), CRK, IKK (cat), FRS2, SHANK3, LIMK1, IL-22, Rac1, IP3 receptor, PI3K reg class IA (p85), Cyclin A2, IKK-alpha, alpha-V/beta-3 integrin, N-Ras, GFRalpha1, CREB1, Bcl-XL, MEK1/2, NF-kB, AKT(PKB), PDK (PDPK1), c-Src, RhoA, ITGB1, PI3K cat class IA, HIF1A, GFRalpha2, F-Actin cytoskeleton, SOS, C3G, NFKBIA, CDK2, STAT3, GDNF, ROCK, Calmodulin, MEKK1(MAP3K1), SHP-2, IKK-beta, Bcl-2, Cyclin D1, JNK2(MAPK9), Elk-1, Cofilin |
| 17 | IGF family signaling in colorectal cancer | 7.25E-11 | GRB2, c-Fos, IRS-2, IRS-1, H-Ras, eIF4E, ERK1/2, Clusterin, Beta-catenin, IKK (cat), Rad51, GIPC, I-kB, GSK3 beta, MAFG, IKK-alpha, IGF-2, c-Myb, GSK3 alpha/beta, Bcl-XL, IGF-1 receptor, MEK1/2, NF-kB, AKT(PKB), ERK1 (MAPK3), IBP, RelA (p65 NF-kB subunit), PI3K reg class IA (p85-alpha), HIF1A, SOS, IGF-2 receptor, 4E-BP1, PI3K cat class IA (p110-alpha), ZNF143, PTEN, MNK2(GPRK7), Cyclin D1, MAT2A, mTOR, FosB |
| 18 | Immune response_IL-3 signaling via JAK/STAT, p38, JNK and NF-kB | 1.05E-10 | STAT5A, MHC class II, Cyclin D3, c-Fos, JAK1, Tyk2, XBP1, H-Ras, IKK (cat), Fibronectin, STAT1, Rac1, BAD, Cyclin D2, I-kB, CISH, Cyclin A2, IKK-alpha, IL-6, Bcl-XL, NF-kB, IRE1, AKT(PKB), NF-kB p50/p65, c-Src, Mcl-1, P-selectin, IL-2R alpha chain, C3aR, PKM2, ARNT, ITGB1, PI3K cat class IA, C/EBPbeta, CSF2RB, TRAF6, MEK3(MAP2K3), RXRA, 14-3-3 gamma, Bcl-6, MKK7 (MAP2K7), CD69, STAT3, IKK-beta, Bcl-2, MKP-1, NOTCH1 precursor, Oncostatin M, STAT5, Cyclin D1, STAT6, Ephrin-B2, p38 MAPK, BMP2 |
| 19 | Stellate cells activation and liver fibrosis | 1.53E-10 | COL1A1, GRB2, c-Fos, CCL2, H-Ras, Beta-catenin, IKK (cat), TRADD, SMAD4, DAB2, SMAD2, Dsh, I-kB, PI3K reg class IA (p85), GSK3 beta, MyD88, RIPK1, MEK2(MAP2K2), MMP-2, IL-1RI, COL1A2, AKT(PKB), PDGF receptor, NF-kB p50/p65, TNF-R1, ERK1 (MAPK3), PI3K cat class IA, TRAF2, Tcf(Lef), TGF-beta receptor type II, TRAF6, IL1RAP, KLF6, SOS, TIMP1, Cyclin D1, IRAK1/2, GLI-1, SMAD3, SP1, Elk-1, Frizzled, TGF-beta receptor type I, PDGF-R-beta |
| 20 | Apoptosis and survival_NGF/ TrkA PI3K-mediated signaling | 1.58E-10 | TRIO, AKT1, Calcineurin A (catalytic), GRB2, GAB1, H-Ras, PAK2, ERK1/2, PKC-zeta, SSH2L, LIMK1, Rac1, BAD, RhoG, PI3K reg class IA (p85), GSK3 beta, FKHR, KIDINS220, CREB1, Actin cytoskeletal, AKT(PKB), PDK (PDPK1), c-Src, Tubulin (in microtubules), BETA-PIX, RhoA, PI3K cat class IA, PARD3, Destrin, p190RhoGAP, Myosin II, SOS, 4E-BP1, ROCK, MRCKalpha, FOXO4, Calmodulin, N-WASP, MRLC, Bcl-2, FOXO3A, PARD6A, ARAP3, SSH1L, Cofilin, mTOR, PREX1 |
| 21 | Cytoskeleton remodeling_Regulation of actin cytoskeleton nucleation and polymerization by Rho GTPases | 1.66E-10 | RhoF (Rif), HDIA2, BAIAP2, FMNL3, Rac2, mDIA2(DIAPH3), RhoC, Gelsolin, CYFIP1, Rac1, RhoD, DRF, MENA, Profilin, CYFIP2, Cdc42 subfamily, VASP, RhoA-related, Actin cytoskeletal, FHOD1, RhoA, DAAM1, F-Actin cytoskeleton, WASF subunit, RhoB, PIP5KI, FNBP1, Rac1-related, N-WASP, WRC, TC10, WASF2, FNBP1L |
| 22 | Development_Negative regulation of WNT/Beta-catenin signaling in the nucleus | 1.69E-10 | TBL1X, Casein kinase I delta, AKT1, Calcineurin A (catalytic), BACH1, HBP1, Oct-3/4, VHL, PGAM5, Alpha-1 catenin, TCF7L2 (TCF4), 14-3-3, Jade-1, Beta-catenin, BCL9/B9L, PC1-CTT, TLE, CBP/P300, Nitrilase 1, Dsh, KLF4, Menin, NF-AT5, GSK3 beta, ICAT, P15RS, RANBP3, CtBP, TBLR1, PJA2, HIC5, Axin, TAK1(MAP3K7), TCF7 (TCF1), TRRAP, NARF, Tcf(Lef), TRIM33, GLI-3R, CHD8, SENP2, Kaiso, NLK, RNF43, WNT, FOXO3A, CHIBBY, Plakoglobin, RUVBL2, Frizzled, Histone H1, DACT1 |
| 23 | Cell adhesion_Tight junctions | 1.76E-10 | Rich1, JAM2, EPB41, MPP5, F-Actin, ARP3, INADL, AF-6, ACTR3, PKC-zeta, Actin, ZO-2, CRB3, JAM3, Myosin VIIA, Cortactin, Actin cytoskeletal, PARD6, Tubulin (in microtubules), RhoA, Cingulin, PARD3, ZO-1, Myosin II, ROCK, Actomyosin, PDZ-RhoGEF, Tubulin alpha, N-WASP, MRLC, CGNL1, Occludin |
| 24 | Signal transduction_AKT signaling | 4.9E-10 | Tuberin, RHEB2, Cyclin D3, GAB1, IRS-1, IKK (cat), BAD, I-kB, PI3K reg class IA, IKK-alpha, MDM2, GSK3 alpha/beta, Bcl-XL, IGF-1 receptor, NF-kB, AKT(PKB), HSP90, PDK (PDPK1), p27KIP1, PI3K cat class IA, Cyclin D, PP2A catalytic, Hamartin, GYS1, PCNA, 4E-BP1, PTEN, FOXO3A, HGF receptor (Met), Bim, mTOR |
| 25 | Translation_Regulation of EIF4F activity | 6.5E-10 | Tuberin, EGF, RHEB2, GRB2, IRS-1, H-Ras, eIF4E, ERK1/2, PKC-zeta, Rac1, PI3K reg class IA, IGBP1, eIF4G2, MEK2(MAP2K2), MSK1, AKT(PKB), PDK (PDPK1), TAK1(MAP3K7), MLK3(MAP3K11), PI3K cat class IA, TGF-beta receptor type II, MEK3(MAP2K3), eIF4B, SOS, PP2A catalytic, eIF4G1/3, eIF4G1, Hamartin, 4E-BP1, MEKK1(MAP3K1), eIF4A, TAB1, p70 S6 kinase2, p38 MAPK, mTOR, TGF-beta receptor type I |
| 26 | Immune response_IFN-alpha/beta signaling via PI3K and NF-kB pathways | 6.91E-10 | Tuberin, AKT1, Cyclin D3, ISG15, JAK1, IRS-2, IL-10, Tyk2, IRS-1, NMI, eIF4E, ERK1/2, IKK (cat), IFNAR1, CDK1 (p34), I-kB, p70 S6 kinases, PI3K reg class IA (p85), GSK3 beta, NF-kB2 (p100), Rb protein, CDK4, EMSY, IKK-alpha, CAK complex, CREB1, p130, MEK1/2, RSAD2, NF-kB, AKT(PKB), PDK (PDPK1), p16INK4, p107, p27KIP1, SLFN5, PI3K cat class IA, RelA (p65 NF-kB subunit), NF-kB2 (p52), TRAF2, eIF4B, eIF4G1/3, CDK2, STAT3, PCNA, 4E-BP1, GBP1, IFIT1, PKC-alpha, eIF4A, FOXO3A, MNK2(GPRK7), Cyclin A |
| 27 | Immune response_IL-1 signaling pathway | 7.77E-10 | SPHK1, p38alpha (MAPK14), JAM2, IP10, ECSIT, CD44, CCL2, ERK1/2, PKC-zeta, EGR1, IKK (cat), MEK4/7, STAT1, I-kB, PI3K reg class IA (p85), NF-kB2 (p100), MAPKAPK2, NF-kB p52/RelB, IL-1RI, IL-6, MEK1/2, NF-kB, AKT(PKB), NF-kB p50/p65, PDK (PDPK1), MYLK1, TAK1(MAP3K7), c-IAP2, PI3K cat class IA, RelA (p65 NF-kB subunit), NF-kB1 (p105), NF-kB1 (p50), NF-kB2 (p52), MMP-1, ZFP36(Tristetraprolin), TRAF6, MEK3(MAP2K3), IL1RAP, FGF2, PGES, PLAU (UPA), CCL7, KHSRP, MEKK1(MAP3K1), MAP3K3, IRAK1, JNK(MAPK8-10), Elk-1 |
| 28 | PI3K signaling in gastric cancer | 8.71E-10 | EGF, IRS-1, Beta-catenin, IKK (cat), CCXCR1, G-protein alpha-q/11, MDR1, BAD, I-kB, PI3K reg class IA (p85), GSK3 beta, PI3K reg class IA, IKK-alpha, alpha-V/beta-3 integrin, Cyr61, AKT(PKB), NF-kB p50/p65, Gastrin 17, PDK (PDPK1), Progastrin, c-Src, HGF, PI3K cat class IA, RelA (p65 NF-kB subunit), PI3K reg class IA (p85-alpha), HIF1A, CBL-B, ELAVL1 (HuR), PI3K cat class IA (p110-alpha), PTEN, G-protein alpha-q, Cyclin D1, HGF receptor (Met), BMP2 |
| 29 | Development_Regulation of epithelial-to-mesenchymal transition (EMT) | 1.34E-09 | Arkadia, EGF, NOTCH1 receptor, E2A, Sno-N, Caldesmon, SMAD2, Fibronectin, FGFR1, SIP1 (ZFHX1B), MMP-2, IL-1RI, CREB1, TNF-R1, PDGF-D, ACTB, HGF, HEY1, E-cadherin, TGF-beta 2, RelA (p65 NF-kB subunit), TGF-beta receptor type II, ZO-1, FGF2, TCF8, TGF-beta 3, ATF-2, WNT, SLUG, Bcl-2, Oncostatin M, Occludin, SP1, HGF receptor (Met), Jagged1, PAI1, Frizzled, EDNRA, TGF-beta receptor type I, PDGF-R-beta |
| 30 | Development_Stimulation of differentiation of mouse embryonic fibroblasts into adipocytes by extracellular factors | 1.35E-09 | Tuberin, CBP, AKT1, BMP receptor 2, GRB2, IRS-2, IRS-1, NGAL, H-Ras, ATF-1, ERK1/2, p90RSK2(RPS6KA3), PKA-reg (cAMP-dependent), SMAD4, SREBP1 precursor, EGR2 (Krox20), PI3K reg class IA (p85), Prostacyclin receptor, FKHR, KLF15, Adenylate cyclase, MEK2(MAP2K2), CREB1, IGF-1 receptor, PDK (PDPK1), ERK1 (MAPK3), A-FABP, Lysyl oxidase, DRIP130, C/EBPbeta, SOS, PI3K cat class IA (p110-alpha), ATF-2, SHP-2, TAB1, BMPR1A, AKT2, SMAD1, p38 MAPK, Elk-1, PKA-cat (cAMP-dependent), BMP2, mTOR |
