# Supplementary material for: Prognoses and genomic analyses of proteasome 26S subunit, ATPase (PSMC) family genes in clinical breast cancer
Source: Aging (Albany NY). 2021 Jul 30;13(14):17970. doi: 10.18632/aging.203345 (PMC8351721; doi:10.18632/aging.203345)
Supplement: Supplementary Table 7 [file aging-13-203345-s007.docx]

**Supplementary Table 7. Pathway analysis of genes coexpressed with proteasome 26S subunit, ATPase 5 (PSMC5) from public breast cancer databases using the MetaCore database (with p<0.01 set as the cutoff value).**

| # | Map | *p* Value | Network objects from active data |
| --- | --- | --- | --- |
| 1 | Chemotaxis_Lysophosphatidic acid signaling via GPCRs | 2.41E-15 | Tiam1, E-cadherin, cPKC (conventional), G-protein alpha-12 family, AKT1, LPAR3, c-Fos, alpha-6/beta-1 integrin, LPAR1, PI3K cat class IA (p110-beta), PKC-delta, Tcf(Lef), IL-8, EGFR, G-protein alpha-i family, H-Ras, TRAF6, F-Actin cytoskeleton, LARG, MKL2, ERK1/2, PRK1, ARHGEF1 (p115RhoGEF), HB-EGF, PKC-zeta, Beta-catenin, EGR1, CRK, G-protein alpha-q/11, PKC-epsilon, TRIP6, Rac1, IP3 receptor, E3KARP (NHERF2), G-protein gamma 12, MEK4(MAP2K4), LPAR2, MLCP (reg), ROCK, PRKD1, PAK, p70 S6 kinase1, PDZ-RhoGEF, PKC, PLC-beta, FKHR, Vinculin, PLEKHG2, ATF-2, HAS2, LPAR6, FasR(CD95), Cyr61, SIVA1, Bcl-XL, G-protein beta/gamma, Rho GTPase, Actin cytoskeletal, N-CoR, TAZ, MSK1, CTGF, AKT(PKB), PDK (PDPK1), c-Src, PLC-beta3, ADAM17, PLC-epsilon, p38 MAPK, Elk-1 |
| 2 | Oxidative stress_ROS-induced cellular signaling | 2.72E-15 | NOTCH1 (NICD), p38alpha (MAPK14), Thioredoxin, Heme oxygenase 1, ACACA, RelA (p65 NF-kB subunit), SREBP1 (nuclear), IRP1, SCD, IL-8, ERK1/2, AMPK alpha subunit, EGR1, VEGF-A, PKA-reg (cAMP-dependent), TXNIP (VDUP1), Pin1, NIK(MAP3K14), Bak, Cytochrome c, p300, FASN, NFKBIA, HSPA1A, TfR1, Syk, COX-2 (PTGS2), IL-1 beta, PKC-beta, PRKD1, Sirtuin1, p70 S6 kinase1, PKC, LKB1, MEKK1(MAP3K1), HES1, FTL, IKK-alpha, HSP27, FTH1, DLC1 (Dynein LC8a), IRP2, GRP75, c-Abl, IL-6, NOXA, Cyclin D1, HIF-prolyl hydroxylase, HDAC1, NF-kB, SAE2, AKT(PKB), Catalase, NF-kB p50/p65, Cyclin B1, NRF2, c-Src, ADAM17, p38 MAPK, APEX, NALP3, TNF-alpha |
| 3 | Development_Negative regulation of WNT/Beta-catenin signaling in the cytoplasm | 1.4E-12 | RACK1, E-cadherin, HECTD1, Prickle-1, Casein kinase I delta, NOTCH1 receptor, c-Cbl, CXXC4, VHL, HIPK2, DP1, Tcf(Lef), E2F1, PP1-cat, HUWE1, Amer1, Alpha-1 catenin, Laforin, Beta-catenin, PP2A catalytic, PI3K cat class III (Vps34), CXXC5, SENP2, Dsh, RNF185, A20, YAP1/TAZ, Rac1, CDK6, PEG3, WNT5A, Cul2/Rbx1 E3 ligase, Malin, STK4, Porf-2, WNT, Beclin 1, NEDD4L, GSK3 alpha/beta, Ankyrin-G, LATS2, G-protein beta/gamma, Skp2/TrCP/FBXW, TAZ, Cyclin D1, Axin, Frizzled, DACT1 |
| 4 | Cell adhesion_Tight junctions | 1.5E-12 | Rich1, JAM2, PARD3, MUPP1, EPB41, MPP5, F-Actin, ARP3, INADL, AF-6, ZO-1, ACTR3, PKC-zeta, Myosin II, Claudin-1, Actin, DNMBP(TUBA), ZO-2, CRB3, ROCK, Actomyosin, JAM3, PDZ-RhoGEF, Tubulin alpha, PKC-lambda/iota, N-WASP, MRLC, CGNL1, Actin cytoskeletal, Angiomotin (AMOT), PARD6, Tubulin (in microtubules) |
| 5 | Development_Negative regulation of WNT/Beta-catenin signaling in the nucleus | 4.11E-12 | Casein kinase I delta, AKT1, TRRAP, Calcineurin A (catalytic), NARF, RUNX3, c-Cbl, HBP1, VHL, PKC-delta, Tcf(Lef), E2F1, Alpha-1 catenin, TCF7L2 (TCF4), 14-3-3, Jade-1, Beta-catenin, VEGF-A, TRIM33, BCL9/B9L, GLI-3R, CHD8, TLE, SENP2, CBP/P300, Nitrilase 1, Dsh, Menin, NF-AT5, RNF43, WNT5A, Nephrocystin-4, SOX2, WNT, FOXO3A, GPX4, LATS2, CHIBBY, RANBP3, CtBP, HDAC1, TAB2, PJA2, RUVBL2, Axin, TAK1(MAP3K7), SOX9, Frizzled, Histone H1, DACT1 |
| 6 | Immune response_B cell antigen receptor (BCR) pathway | 1.17E-11 | RelA (p65 NF-kB subunit), LRRK1, Calcineurin A (catalytic), NCK1, c-Fos, NF-kB1 (p50), alpha-4/beta-1 integrin, c-Rel (NF-kB subunit), ETS1, Lyn, H-Ras, NF-kB p50/c-Rel, ERK1/2, CIN85, EGR1, PP2A catalytic, BCAP, SOS1, PIP5KI, Btk, PKC-beta2, NFKBIA, PI3K cat class IA (p110-delta), Rac1, IP3 receptor, CDK6, Syk, Cyclin D2, PKC-beta, p70 S6 kinase1, Rb protein, Calmodulin, CalDAG-GEFIII, CDK4, MEKK1(MAP3K1), FKHR, ATF-2, IKK-alpha, CalDAG-GEFII, N-Ras, PLC-gamma, VAV-2, GSK3 alpha/beta, Bcl-XL, Actin cytoskeletal, Bcl-10, NF-kB, AKT(PKB), DAPP1, NF-kB p50/p65, PDK (PDPK1), PLC-gamma 2, p38 MAPK, Elk-1, TAK1(MAP3K7), NF-AT2(NFATC1), MEKK4(MAP3K4) |
| 7 | Development_Positive regulation of WNT/Beta-catenin signaling in the nucleus | 3.09E-11 | CBP, TWA1, DP1, Tcf(Lef), Alpha-1 catenin, TCF7L2 (TCF4), FOXP1, FOXM1, USP5, Jade-1, Beta-catenin, BCL9/B9L, Pin1, FOXK1, TLE, ZIP-kinase, p300, CBP/P300, NCOA2 (GRIP1/TIF2), FAM53B, Dsh, UBR5, Kindlin-2, Sirtuin1, CARF, UCHL5, FHL2, WIP1, RUNX, WNT, FOXK2, FOXO3A, HMGB2, SOX4, HDAC1, APPL, ERK2 (MAPK1), RUVBL2, SOX9, Frizzled, JRK |
| 8 | Signal transduction_Calcium-mediated signaling | 4.11E-11 | MLCP (cat), Tiam1, cPKC (conventional), RelA (p65 NF-kB subunit), Calcineurin A (catalytic), c-Fos, ERK1/2, 14-3-3, AMPK alpha subunit, Myosin II, EGR1, HDAC4, MEF2, HDAC5, p300, MUNC13, Rac1, IP3 receptor, RhoGDI alpha, MEK4(MAP2K4), COX-2 (PTGS2), PPCKC, MLCP (reg), I-kB, PKC-beta, ROCK, Calmodulin, PKC, MYH11, PPA5, ATF-2, TORC2, CaMKK, PPARGC1 (PGC1-alpha), Bcl-10, NURR1, NF-kB, AKT(PKB), p38 MAPK, Elk-1, NF-AT2(NFATC1), CaMKK2 |
| 9 | Development_Negative regulation of STK3/4 (Hippo) pathway and positive regulation of YAP/TAZ function | 4.4E-11 | MLCP (cat), G-protein alpha-12 family, PARD3, LPAR3, LPAR1, CD44, HIPK2, ASPP1, EGFR, ASPP2, LARG, ERK1/2, ARHGEF1 (p115RhoGEF), AGTR1, RASSF6, G-protein alpha-q/11, Angiotensin II, WBP-2, NEDD4, ILK, ZO-2, LPAR2, MOBKL1A, MLCP (reg), WW45, PDZ-RhoGEF, ARHGEF2, Nephrocystin-4, FRMD4A, STK4, PP1-cat alpha, LATS2, Actin cytoskeletal, TAZ, PJA2, PDK (PDPK1), Angiomotin (AMOT), ERK1 (MAPK3) |
| 10 | Stellate cells activation and liver fibrosis | 5.86E-11 | GRO-2, TNF-R2, PI3K cat class IA, PDGF-B, c-Fos, PTCH1, WNT3A, Tcf(Lef), TGF-beta receptor type II, H-Ras, TRAF6, IL1RAP, KLF6, Beta-catenin, TRADD, SOS, SMAD4, PDGF-R-alpha, NIK(MAP3K14), SARA, Dsh, Smoothened, TIMP1, IL-1 beta, I-kB, RIPK1, MMP-2, IL-1RI, Cyclin D1, COL1A2, ERK2 (MAPK1), AKT(PKB), PDGF receptor, NF-kB p50/p65, TNF-R1, ERK1 (MAPK3), Elk-1, Frizzled, TLR2, PDGF-R-beta, TNF-alpha |
| 11 | Cytoskeleton remodeling_Regulation of actin cytoskeleton organization by the kinase effectors of Rho GTPases | 8.51E-11 | MLCP (cat), BETA-PIX, RhoJ, Destrin, F-Actin cytoskeleton, Spectrin, PRK1, Myosin II, Caldesmon, Alpha-actinin, PAK1, RhoC, PIP5KI, MyHC, Rac3, Rac1, RhoGDI alpha, Talin, MLCP (reg), GIT1, ROCK, DMPK, Actomyosin, PAK, Rac1-related, Cdc42 subfamily, Vinculin, MSN (moesin), ERM proteins, MRLC, ARPC1B, RhoA-related, Actin cytoskeletal, Filamin A, TC10, MLCK |
| 12 | Signal transduction_Angiotensin II/AGTR1 signaling via Notch, Beta-catenin and NF-kB pathways | 9.56E-11 | NOTCH1 (NICD), E-cadherin, CBP, RelA (p65 NF-kB subunit), NOTCH1 receptor, IL-8, TRAF6, TCF7L2 (TCF4), ERK1/2, AGTR1, Beta-catenin, VEGF-A, TRPC6, PAK1, NIK(MAP3K14), Angiotensin II, p300, Rac1, I-kB, PRKD1, gamma-Secretase complex, PKC, HES1, WISP1, IKK-alpha, Connexin 43, MMP-2, IL-6, HEY2, Angiotensinogen, Cyclin D1, NCOA1 (SRC1), NF-kB, CTGF, ERK2 (MAPK1), AKT(PKB), NF-kB p50/p65, NOTCH1 (NEXT), PDK (PDPK1), c-Myc, ADAM17, p38 MAPK, TAK1(MAP3K7) |
| 13 | Leptin signaling in colorectal cancer | 1.04E-10 | E-cadherin, JAK2, PI3K cat class IA, RelA (p65 NF-kB subunit), c-Fos, PKC-delta, IL-8, TCF7L2 (TCF4), ERK1/2, Beta-catenin, Matrilysin (MMP-7), VEGF-A, Leptin receptor, NFKBIA, Rac1, CDK1 (p34), STAT3, p70 S6 kinase1, Survivin, PI3K reg class IA, IKK-alpha, IL-6, Bcl-XL, Cyclin D1, AKT(PKB), Cyclin B1, c-Src, Leptin, c-Myc, ADAM17 |
| 14 | Androgen receptor activation and downstream signaling in Prostate cancer | 1.6E-10 | E-cadherin, gp130, GAB1, JAK1, c-Cbl, NCOA3 (pCIP/SRC3), PI3K cat class IA (p110-beta), PKC-delta, STEAP1, KLF5, IL-8, EGFR, H-Ras, FGF1, Versican, Clusterin, Matrilysin (MMP-7), SGK1, VEGF-A, FGF2, SOS, FRS2, FEN1, APP, Bak, SPRY1, NCOA2 (GRIP1/TIF2), FGFR1, IL6RA, Kallikrein 3 (PSA), VIL2 (ezrin), STAT3, Rb protein, ErbB2, ADAM9, FRS2beta, N-Ras, PELP1, Caspase-2, IL-6, Bcl-XL, Cyclin D1, NCOA1 (SRC1), ERK2 (MAPK1), PSCA, AKT(PKB), Caveolin-1, IGF-1, PDK (PDPK1), c-Src, c-Myc, ADAM17, ERK1 (MAPK3), IL-6 receptor, TLR2 |
| 15 | Signal transduction_Angiotensin II/ AGTR1 signaling via p38, ERK and PI3K | 2.97E-10 | Pyk2(FAK2), JAK2, PI3K cat class IA, RECK, PDGF-B, PKC-delta, KLF5, ETS1, EGFR, H-Ras, ERK1/2, MEF2C, SP3, GCN5, AGTR1, HB-EGF, PKC-zeta, EGR1, MEF2A, MSK1/2 (RPS6KA5/4), HDAC4, SOS, Angiotensin II, p90Rsk, Syk, COX-2 (PTGS2), p70 S6 kinases, Calmodulin, FKHR, WISP1, CalDAG-GEFII, MMP-2, IL-6, G-protein beta/gamma, PPARGC1 (PGC1-alpha), Fyn, G-protein alpha-q, Cyclin D1, AKT(PKB), ATOX1, Catalase, OSF-2, PDK (PDPK1), c-Src, ADAM17, p38 MAPK, Elk-1, PDGF-D, PDGF-R-beta, ATP7A, MEKK4(MAP3K4) |
| 16 | Ligand-independent activation of Androgen receptor in Prostate Cancer | 1.07E-09 | JAK2, PI3K cat class IA, GAB1, WNT3A, NCOA3 (pCIP/SRC3), Tcf(Lef), EGFR, H-Ras, FGF1, Beta-catenin, PP2A regulatory, FGF2, SOS, PP2A catalytic, FRS2, NCOA2 (GRIP1/TIF2), DDX5, STAT5B, FGFR1, Kallikrein 3 (PSA), STAT3, Tip60, ErbB2, PI3K reg class IA, FRS2beta, N-Ras, c-Abl, Bcl-XL, Cyclin D1, NCOA1 (SRC1), HDAC1, ERK2 (MAPK1), AKT(PKB), IGF-1, PDK (PDPK1), c-Myc, ERK1 (MAPK3), Frizzled |
| 17 | Apoptosis and survival_Regulation of apoptosis by mitochondrial proteins | 1.21E-09 | p38alpha (MAPK14), NIX, Maspin, PLSCR3, Calcineurin A (catalytic), NOR1, IFI27, Aif, GC1QBP, PKC-delta, Apaf-1, LETM1, ERK1/2, Parkin, MUL1, RAD9A, Pin1, PP2C, PP2A catalytic, PARL, VDAC 2, Bak, Cytochrome c, Caspase-8, MFF, Metaxin 1, OPA1, RAD9, Bcl-B, OMA1, Caspase-10, SOD1, PINK1, ATF-2, SLC25A3, tBid, PP1-cat alpha, Beclin 1, Mitofusin 1, Caspase-2, Bcl-XL, NOXA, HtrA2, Cyclin A, MIDUO, Caveolin-1, Calpain 1(mu), DNM1L (DRP1), Mcl-1, p38 MAPK, Cathepsin D, Bid |
| 18 | Signal transduction_Angiotensin II/ AGTR1 signaling via RhoA and JNK | 1.27E-09 | PU.1, MLCP (cat), Pyk2(FAK2), JAK2, LBC, G-protein alpha-12 family, RECK, NCK1, c-Fos, ROCK2, PKC-delta, G-protein alpha-12, LARG, ERK1/2, ARHGEF1 (p115RhoGEF), AGTR1, VEGF-A, CRK, PAK1, G-protein alpha-q/11, Angiotensin II, PLC-beta1, Rac1, IP3 receptor, MEK4(MAP2K4), MLCP (reg), ROCK, PDZ-RhoGEF, Calmodulin, Endothelin-1, MEKK1(MAP3K1), Vinculin, ATF-2, MRLC, MMP-2, G-protein beta/gamma, Cyclin D1, CTGF, c-Src, PLK1, ECT2, MLCK |
| 19 | Development_SLIT-ROBO1 signaling | 1.28E-09 | Rictor, PI3K cat class IA, Calcineurin A (catalytic), NCK1, ROBO1, SRGAP1, F-Actin cytoskeleton, PAK2, SLIT3, Myosin II, LSP1, Rac1, ROCK, MENA, Calmodulin, NCK2, FLII, SLIT1, SLIT2, Fyn, Cytohesin3, AKT(PKB), Cytohesin1, SSH1L, Endophilin A2, CXCR4, ACTB |
| 20 | Transcription_HIF-1 targets | 1.48E-09 | G3P2, NIX, Heme oxygenase 1, TGF-beta 2, DEC2, PDGF-B, Lysyl oxidase, ROR-alpha, CITED2, P4HA2, GPI, VEGF-A, Cyclin G2, 5'-NTD, FGF2, GLUT3, Angiopoietin 2, MGF, Thrombospondin 1, MSH6, MDR1, AK3, TfR1, Mxi1, MSH2, ALDOA, F263, Transferrin, Endothelin-1, SOX2, TGF-beta 3, CX3CR1, LOXL4, MMP-2, Galectin-1, NOXA, CTGF, MCT4, ABCG2, HGF receptor (Met), ALDOC, Leptin, c-Myc, Mcl-1, SDF-1, CXCR4, LRP1, PKM2 |
| 21 | Signal transduction_PDGF signaling via MAPK cascades | 1.54E-09 | SPHK1, NOR1, PDGF-B, c-Fos, GAB1, PKC-delta, H-Ras, ERK1/2, EGR1, TRPC6, PDGF-R-alpha, SOS1, Tissue factor, p90Rsk, Rac1, COX-2 (PTGS2), GIT1, MAPKAPK2, HSP27, VAV-2, MMP-2, c-Abl, IL-6, Cyclin D1, PDGFR-ab, ERK2 (MAPK1), PDGF receptor, Stromelysin-1, c-Src, p27KIP1, c-Myc, p38 MAPK, Elk-1, GRB10, PDGF-D, Phox1 (PRRX1), PDGF-R-beta |
| 22 | Cell adhesion_Integrin-mediated cell adhesion and migration | 2.22E-09 | Tensin, BETA-PIX, alpha-3/beta-1 integrin, Tiam1, ITGB1, p190-RhoGEF, PARD3, alpha-6/beta-1 integrin, alpha-4/beta-1 integrin, PINCH, F-Actin cytoskeleton, DOCK1, LARG, ELMO1, ARHGEF1 (p115RhoGEF), PKC-zeta, Alpha-actinin, CRK, Beta-parvin, PAK1, ILK, Rac1, Syk, Talin, PKC, ITGA4, Vinculin, PKC-lambda/iota, VASP, VAV-2, Actin cytoskeletal, Alpha-parvin, GIT2, Collagen IV, c-Src, PARD6 |
| 23 | Immune response_IL-1 signaling pathway | 2.4E-09 | SPHK1, p38alpha (MAPK14), Sequestosome 1(p62), JAM2, c-IAP2, PI3K cat class IA, RelA (p65 NF-kB subunit), NF-kB1 (p105), NF-kB1 (p50), ECSIT, CD44, IL-8, ZFP36(Tristetraprolin), TRAF6, IL1RAP, ERK1/2, PKC-zeta, EGR1, FGF2, PGES, NIK(MAP3K14), MEK4/7, MEK4(MAP2K4), COX-2 (PTGS2), IL-1 beta, I-kB, MAPKAPK2, GRO-1, RUNX2, MEKK1(MAP3K1), MAP3K3, HSP27, IL-1RI, IL-6, TPL2(MAP3K8), NF-kB, AKT(PKB), NF-kB p50/p65, PDK (PDPK1), MYLK1, Elk-1, TAK1(MAP3K7), TNF-alpha |
| 24 | Cell cycle_Influence of Ras and Rho proteins on G1/S Transition | 2.6E-09 | MLCP (cat), MLK3(MAP3K11), PI3K cat class IA, RelA (p65 NF-kB subunit), ROCK2, E2F1, H-Ras, ERK1/2, PAK1, NFKBIA, Rac1, MEK4(MAP2K4), CDK6, STAT3, MLCP (reg), p70 S6 kinase1, Rb protein, PI3K reg class IA, CDK4, Cyclin A2, ATF-2, IKK-alpha, Tob1, MRLC, RGL2, Cyclin D1, AKT(PKB), NF-kB p50/p65, PDK (PDPK1), p27KIP1, c-Myc, MLCK |
| 25 | Development_Regulation of lung epithelial progenitor cell differentiation | 2.96E-09 | p38alpha (MAPK14), SMAD5, E-cadherin, NOTCH1 receptor, WNT3A, Tcf(Lef), BMP4, FOXP1, O-fucose, Napsin A, TITF1, SMAD9 (SMAD8), Beta-catenin, SMAD4, FOXJ1, Podoplanin, STAT3, Aquaporin 5, WNT, ELF5, BMPR1A, SMAD1, Jagged1, FOXP2, N-Myc, Frizzled, ERM |
| 26 | Cytoskeleton remodeling_PDGF signaling via calcium and Rho GTPases | 3.91E-09 | SLC31A1, Tiam1, Pyk2(FAK2), ABL2, CDK5, AKT1, PDGF-B, Lysyl oxidase, NCK1, F-Actin, DOCK1, CACNA1C, Beta-catenin, EBP50, IQGAP1, TRPC6, PAK1, PDGF-R-alpha, WASF subunit, WASF3 (WAVE3), PKC-epsilon, Rac1, IP3 receptor, E3KARP (NHERF2), Calmodulin, ARP2, PKC, Vinculin, N-WASP, VAV-2, c-Abl, Actin cytoskeletal, Fyn, PDGFR-ab, ATOX1, PDGF receptor, WRC, ALPHA-PIX, c-Src, PDGF-D, WaspIP, PDGF-R-beta, ATP7A |
| 27 | Cell cycle_Regulation of G1/S transition (part 2) | 4.53E-09 | E2F1/DP1 complex, RelA (p65 NF-kB subunit), Cyclin D3, c-Fos, DP1, E2F1, ERK1/2, Cyclin D, CDK6, Cyclin D2, E2F4/DP1 complex, Cyclin A1, Rb protein, E2F4, CDK4, Cyclin A2, IKK-alpha, CAK complex, Cyclin A, Cyclin D1, AKT(PKB) |
| 28 | Neurophysiological process_Dynein-dynactin motor complex in axonal transport in neurons | 5.01E-09 | Importin (karyopherin)-beta, CDK5, Dynein 1, cytoplasmic, light chains, TrkC, ERK1/2, DCTN1(p150Glued), Importin (karyopherin)-alpha, Sortilin, APP, TrkB, DYNC1I1, Ubiquitin, TMEM108, Rab-5A, Centractins, DYNLL, ORP1, Rab-7, Dynein 1, cytoplasmic, heavy chain, Tctex-1, Kinesin heavy chain, MAPRE3(EB3), DYNLT, PRNP, AKT(PKB), Kinesin light chain, Snapin, PAFAH alpha (LIS1), Dynein 1, cytoplasmic, intermediate chains, DYI2, NUDEL, Tubulin (in microtubules) |
| 29 | Signal transduction_PDGF signaling via PI3K/AKT and NFkB pathways | 5.72E-09 | RelA (p65 NF-kB subunit), PDGF-B, c-Fos, PI3K cat class IA (p110-beta), ETS1, ERK1/2, ETS2, Beta-catenin, YB-1, SGK1, PDGF-R-alpha, SOS1, DDX5, NFKBIA, Rac1, PI3K cat class IA (p110-alpha), p120GAP, Calmodulin, MYH11, FKHR, Tenascin-C, MMP-2, c-Abl, Inversin, Cyclin D1, PDGFR-ab, NF-kB, AKT(PKB), PDGF receptor, NF-kB p50/p65, PDK (PDPK1), c-Src, p27KIP1, c-Myc, Elk-1, PDGF-D, Phox1 (PRRX1), PDGF-R-beta |
| 30 | Immune response_Role of PKR in stress-induced antiviral cell response | 6.57E-09 | p38alpha (MAPK14), RelA (p65 NF-kB subunit), TRAF3, JAK1, PKR, IL-8, TRAF6, ERK1/2, IFN-alpha/beta receptor, Caspase-8, NFKBIA, MEK4(MAP2K4), IL-1 beta, I-kB, ATF-2, IKK-alpha, TARBP2, IL-1RI, IRF7, NFKBIB, IL-6, IRF3, NF-kB, MSK2, TAB2, TRIF (TICAM1), NF-kB p50/p65, TNF-R1, TLR3, c-Myc, TAK1(MAP3K7), TLR2, TNF-alpha |
