# Supplementary material for: Prognoses and genomic analyses of proteasome 26S subunit, ATPase (PSMC) family genes in clinical breast cancer
Source: Aging (Albany NY). 2021 Jul 30;13(14):17970. doi: 10.18632/aging.203345 (PMC8351721; doi:10.18632/aging.203345)
Supplement: Supplementary Table 8 [file aging-13-203345-s008.docx]

**Supplementary Table 8. Pathway analysis of genes coexpressed with proteasome 26S subunit, ATPase 6 (PSMC6) from public breast cancer databases using the MetaCore database (with *p*<0.01 set as the cutoff value).**

| # | Map | *p* Value | Network objects from active data |
| --- | --- | --- | --- |
| 1 | Cytoskeleton remodeling_Regulation of actin cytoskeleton organization by the kinase effectors of Rho GTPases | 7.91E-15 | BETA-PIX, RhoA, WRCH-1, RhoJ, Destrin, F-Actin cytoskeleton, Spectrin, SLC9A1, PRK1, Myosin II, Caldesmon, Alpha-actinin, CPI-17, Alpha adducin, RhoC, LIMK1, RhoB, PIP5KI, MyHC, LIMK, MRCK, Talin, GIT1, ROCK, DMPK, Actomyosin, MRCKalpha, PAK, Rac1-related, Cdc42 subfamily, Vinculin, MSN (moesin), ERM proteins, MRLC, ARPC1B, RhoA-related, CDC42, Actin cytoskeletal, Filamin A, TC10, MLCK |
| 2 | Signal transduction_Calcium-mediated signaling | 3.98E-13 | RhoA, 14-3-3 epsilon, cPKC (conventional), MARK2, RelA (p65 NF-kB subunit), Calcineurin A (catalytic), c-Fos, MEK3(MAP2K3), c-Jun, ERK1/2, 14-3-3, CaMK I, CABIN1, AMPK alpha subunit, CBM complex, Myosin II, EGR1, HDAC4, MEF2, HDAC5, CaMK IV, MUNC13, p47-phox, IP3 receptor, CARD11, I-kB, ROCK, Calmodulin, PKC, NUR77, MYH11, PKC-alpha, MALT1, TORC2, CaMKK, CREB1, PPARGC1 (PGC1-alpha), JNK(MAPK8-10), Bcl-10, NF-kB, AKT(PKB), Elk-1, NF-AT2(NFATC1), IL-2, CaMKK2 |
| 3 | Chemotaxis_Lysophosphatidic acid signaling via GPCRs | 4.63E-11 | RhoA, E-cadherin, cPKC (conventional), G-protein alpha-12 family, AKT1, c-Fos, PKC-delta, Tcf(Lef), EGFR, G-protein alpha-i family, F-Actin cytoskeleton, c-Jun, ERK1/2, PRK1, ARHGEF1 (p115RhoGEF), HB-EGF, Beta-catenin, EGR1, HDAC7, G-protein alpha-q/11, LIMK, TRIP6, PLD2, CD36, IP3 receptor, LPAR2, p130CAS, ROCK, PRKD1, Bax, AP-1, GSK3 beta, Caspase-7, PAK, PDZ-RhoGEF, PKC, PLC-beta, FKHR, Vinculin, PLEKHG2, HAS2, Caspase-3, PLC-delta 1, FasR(CD95), Cyr61, CREB1, Bcl-XL, MEK1/2, G-protein beta/gamma, Rho GTPase, CDC42, Actin cytoskeletal, TAZ, JNK(MAPK8-10), MSK1, MKL1, CTGF, AKT(PKB), c-Src, PLC-beta3, ADAM17, Elk-1, PREX1 |
| 4 | Reproduction_Gonadotropin-releasing hormone (GnRH) signaling | 1.11E-10 | Pyk2(FAK2), GnRH1, GRB2, c-Fos, JunB, ATF-3, PKC-delta, MEK3(MAP2K3), c-Jun, ERK1/2, CaMK I, CACNA1C, EGR1, c-Jun/c-Fos, PKA-reg (cAMP-dependent), HDAC4, SOS, HDAC5, G-protein alpha-q/11, MKK7 (MAP2K7), p90Rsk, IP3 receptor, MEF2D, Shc, Dynamin-1, AP-1, PER1, G-protein alpha-s, Calmodulin, NUR77, PLC-beta, PACAP, Adenylate cyclase, MKP-1, MEK2(MAP2K2), CREB1, CDC42, JNK(MAPK8-10), c-Src, Secretogranin II, Elk-1, PKA-cat (cAMP-dependent) |
| 5 | Development_Positive regulation of STK3/4 (Hippo) pathway and negative regulation of YAP/TAZ function | 1.58E-10 | RhoA, GCGR, Willin, E-cadherin, SCRIB, MPP5, Cullin 2, STK3, beta-TrCP, Alpha-1 catenin, 14-3-3, AMPK alpha subunit, Casein kinase I epsilon, AMPK gamma subunit, Beta-catenin, PKA-reg (cAMP-dependent), PP2A cat (alpha), Alpha-catenin, RASSF2, WW45, MALS-3, Schwannomin (NF2), G-protein alpha-s, LKB1, STK4, Adenylate cyclase, CCDC85C, FasR(CD95), LIF, RASSF5, LATS2, Mol1b, Actin cytoskeletal, Skp2/TrCP/FBXW, TAZ, PEZ, Angiomotin (AMOT), LRR-1, AMOTL1 (Jeap), Axin, PKA-cat (cAMP-dependent) |
| 6 | CHDI_Correlations from Replication data_Causal network (positive correlations) | 1.74E-10 | RhoA, Pyk2(FAK2), MHC class II, PI3K cat class IA, Calcineurin A (catalytic), CD45, Slp76, ICAM1, CD44, MEK3(MAP2K3), c-Jun, PSMC2, MSK1/2 (RPS6KA5/4), HDAC7, MEF2, NIK(MAP3K14), LAT, CaMK IV, IP3 receptor, VIL2 (ezrin), I-kB, ROCK, PKC-theta, Calmodulin, PKC-alpha, ZAP70, CD3, PLC-gamma 1, ITK, Caspase-3, CREB1, IP3R1, G-protein beta/gamma, PSD-95, CD83, JNK(MAPK8-10), CD40(TNFRSF5), NF-kB, AKT(PKB), HSP70, Calcineurin B (regulatory), SDF-1, CXCR4, MEKK4(MAP3K4) |
| 7 | Development_Negative regulation of WNT/Beta-catenin signaling in the cytoplasm | 2.1E-10 | E-cadherin, HECTD1, LRP5/LRP6, Prickle-1, NOTCH1 receptor, c-Cbl, STK3, Tcf(Lef), PP1-cat, RIPK4, Amer1, beta-TrCP, Presenilin 1, Alpha-1 catenin, Casein kinase I epsilon, Beta-catenin, CYLD, PP2A catalytic, DAB2, Nucleoredoxin, SENP2, Dsh, A20, Casein kinase I alpha, YAP1/TAZ, G-protein alpha-13, MAP1LC3A, WWP1, PEG3, STK4, SIAH1, PKC-alpha, Porf-2, WNT, Beclin 1, GSK3 alpha/beta, LATS2, G-protein beta/gamma, Skp2/TrCP/FBXW, TAZ, Cyclin D1, NKD2, DACT3, Axin, Frizzled |
| 8 | Neurophysiological process_Dynein-dynactin motor complex in axonal transport in neurons | 2.24E-10 | Importin (karyopherin)-beta, Dynein 1, cytoplasmic, light chains, TrkC, ERK1/2, Importin (karyopherin)-alpha, HDAC6, TrkB, DYNC1I1, Vimentin, JSAP1, Ubiquitin, Hap-1, NGF, TMEM108, Rab-5A, Centractins, DYNLL, ORP1, Rab-7, Dynein 1, cytoplasmic, heavy chain, Tctex-1, Alpha-centractin, RILP (Rab interacting lysosomal protein), Kinesin heavy chain, MAPRE3(EB3), DYNLT, PRNP, AKT(PKB), Kinesin light chain, Dynein 1, cytoplasmic, intermediate chains, MAPRPE1(EB1), DYI2, NUDEL, Tubulin (in microtubules) |
| 9 | Immune response_IL-3 signaling via JAK/STAT, p38, JNK and NF-kB | 2.28E-10 | Ephrin-B1, PU.1, ARNT, STAT5A, MHC class II, PI3K cat class IA, Cyclin D3, c-Fos, ICAM1, Tyk2, C/EBPbeta, XBP1, Lyn, MEK3(MAP2K3), RXRA, RAR-alpha/RXR-alpha, Bcl-6, MKK7 (MAP2K7), CD69, STAT3, I-kB, SRP9, Survivin, Cyclin A2, DPF3, MKP-1, RARalpha, NOTCH1 precursor, IL-6, Oncostatin M, Bcl-XL, TACI(TNFRSF13B), STAT5, Cyclin D1, IL3RA, CD40(TNFRSF5), NF-kB, STAT6, IRE1, AKT(PKB), NF-kB p50/p65, c-Src, c-Myc, Mcl-1, P-selectin, C3aR, BMP2, PKM2, E-selectin |
| 10 | Oxidative stress_ROS-induced cellular signaling | 3.66E-10 | Casein kinase II, alpha chains, NOTCH1 (NICD), Thioredoxin, Chk2, Tuberin, ACACA, RelA (p65 NF-kB subunit), SREBP1 (nuclear), KEAP1, SCD, HIF1A, SRX1, ERK1/2, AMPK alpha subunit, EGR1, c-Jun/c-Fos, PKA-reg (cAMP-dependent), Pin1, NIK(MAP3K14), Isoform p66 Shc, Cytochrome c, FASN, HSPA1A, GSTP1, Syk, HSF1, NOTCH3 (3ICD), PRKD1, Bax, Sirtuin1, GSK3 beta, GPX1, PKC, LKB1, PTEN, DLC1 (Dynein LC8a), IRP2, GRP75, MDM2, c-Abl, IL-6, GADD45 alpha, Cyclin D1, JNK(MAPK8-10), HIF-prolyl hydroxylase, NF-kB, SAE2, AKT(PKB), NF-kB p50/p65, NRF2, c-Src, ADAM17, APEX, TNF-alpha |
| 11 | Development_PTHR1 in bone and cartilage development | 4.35E-10 | RhoA, G-protein alpha-12 family, MMP-13, c-Fos, PTCH1, G-protein alpha-12, PP1-cat, ERK1/2, MEF2C, PTHR1, ALPL, Beta-catenin, c-Jun/c-Fos, PP2A regulatory, PKA-reg (cAMP-dependent), HDAC4, PP2A catalytic, G-protein alpha-q/11, QSK, PLD2, G-protein alpha-13, MEF2D, GSK3 beta, G-protein alpha-s, PKC, PLC-beta, PKC-alpha, Bone sialoprotein, TORC2, Adenylate cyclase, QIK, MKP-1, LRP6, CREB1, FZD1, IL-6, MEK1/2, Cyclin D1, Osteoprotegerin, IGF-1, PKA-cat (cAMP-dependent), SOX9, BMP2 |
| 12 | Development_Positive regulation of WNT/Beta-catenin signaling in the cytoplasm | 6.26E-10 | Casein kinase II, alpha chains, GSKIP, HECTD1, LRP5/LRP6, BIG1, GRB2, SIAH2, IRS-2, Tcf(Lef), PP1-cat, RIPK4, 14-3-3 zeta/delta, Alpha-1 catenin, c-Jun, 14-3-3, TGT, Beta-catenin, HSP105, TGIF, SMAD4, PP2A catalytic, RNF220, Dsh, USP47, ZBED3, CDK1 (p34), Miz-1, SIAH1, USP9X, USP25, WNT, USP7, GSK3 alpha/beta, PP2C alpha, TBLR1, JNK(MAPK8-10), AKT(PKB), NKD2, Axin, PKA-cat (cAMP-dependent), Frizzled, SET7 |
| 13 | Immune response_M-CSF-receptor signaling pathway | 7.47E-10 | PU.1, RhoA, Pyk2(FAK2), STAT5A, PI3K cat class IA, ERK5 (MAPK7), GRB2, c-Fos, c-Cbl, Tyk2, FMIP, Tcf(Lef), ETS1, PRIM2A, c-Jun, ERK1/2, Beta-catenin, c-Jun/c-Fos, GAB3, STAT5B, PLD2, IP3 receptor, STAT3, Syk, Shc, p120GAP, AP-1, DAP12, Calmodulin, PKC, IL-34, PLC-gamma, MEK2(MAP2K2), CDC42, WASP, Fyn, Cyclin D1, NF-kB, AKT(PKB), c-Src, c-Myc, TSAD, Elk-1 |
| 14 | Neurogenesis_NGF/ TrkA MAPK-mediated signaling | 1.2E-09 | SPHK1, Sequestosome 1(p62), SHPS-1, MATK, ERK5 (MAPK7), GRB2, c-Fos, NF-kB1 (p50), APS, JunB, Fra-1, PKC-delta, MEK3(MAP2K3), c-Jun, ERK1/2, MEF2C, HB-EGF, EGR1, PP2A regulatory, SGK1, PKA-reg (cAMP-dependent), SOS, PP2A catalytic, C3G, SH2B, p90Rsk, IP3 receptor, RAP-1A, p130CAS, NGF, Shc, AP-1, Calmodulin, NUR77, PLC-gamma 1, SNX26 (TCGAP), CREB1, MEK1/2, PLAUR (uPAR), SUR-8, Cyclin D1, M-Ras, MSK1, SORBS1, MMP-10, RGS2, PVR, JMJD3, SHB, c-Src, Elk-1, PKA-cat (cAMP-dependent) |
| 15 | Signal transduction_Angiotensin II/ AGTR1 signaling via p38, ERK and PI3K | 1.63E-09 | Pyk2(FAK2), PI3K cat class IA, ERK5 (MAPK7), GRB2, PKC-delta, KLF5, ETS1, EGFR, eIF4E, ERK1/2, MEF2C, SP3, GCN5, PDGF-A, HB-EGF, EGR1, MSK1/2 (RPS6KA5/4), HDAC4, SOS, MNK1, Angiotensin II, PLD2, p90Rsk, p47-phox, CaMK II delta, Syk, p130CAS, Shc, Calmodulin, NUR77, FKHR, SOD3 (EC-SOD), cPLA2, MMP-14, CREB1, IL-6, MEK1/2, G-protein beta/gamma, PPARGC1 (PGC1-alpha), Fyn, G-protein alpha-q, Cyclin D1, AKT(PKB), c-Src, ADAM17, Elk-1, PKA-cat (cAMP-dependent), PDGF-R-beta, ATP7A, MEKK4(MAP3K4) |
| 16 | Development_VEGF signaling via VEGFR2 - generic cascades | 3.21E-09 | SPHK1, RhoA, Pyk2(FAK2), PI3K cat class IA, Calcineurin A (catalytic), GRB2, PAK2, MEK3(MAP2K3), TCF7L2 (TCF4), c-Jun, eIF4E, ERK1/2, Beta-catenin, c-Jun/c-Fos, SOS, MNK1, p90Rsk, IP3 receptor, I-kB, Shc, COX-1 (PTGS1), p120GAP, GSK3 beta, Calmodulin, PKC, PI3K reg class IA, Vinculin, PKC-alpha, PLC-gamma 1, MEK2(MAP2K2), eNOS, CREB1, PLAUR (uPAR), CDC42, Actin cytoskeletal, Fyn, MSK1, AKT(PKB), HSP90, NF-kB p50/p65, SHB, c-Src, TSAD, Calcineurin B (regulatory), ERK1 (MAPK3), NF-AT2(NFATC1), MLCK |
| 17 | IL-6 signaling in breast cancer cells | 3.5E-09 | E-cadherin, PI3K cat class IA, RelA (p65 NF-kB subunit), GRB2, c-Fos, NF-kB1 (p50), ESR1 (nuclear), C/EBPbeta, ERK1/2, JAB1, SOS, Vimentin, MDR1, STAT3, Shc, MUC1, Survivin, PI3K reg class IA, Fascin, CYP19, IL-6, Bcl-XL, MEK1/2, AKT2, Cyclin D1, SNAIL1, AKT(PKB), Jagged1, c-Myc, Mcl-1, Tensin 4, NOTCH3 |
| 18 | Signal transduction_CXCR4 signaling via MAPKs cascades | 3.5E-09 | Plectin 1, RhoA, RelA (p65 NF-kB subunit), c-Fos, NF-kB1 (p50), G-protein alpha-i family, c-Jun, ERK1/2, EGR1, p90RSK2(RPS6KA3), G-protein alpha-i2, G-protein alpha-13, CD69, Ubiquitin, ROCK, AP-1, PAK, ZAP70, CD3, CD3 zeta, CREB1, IL-6, MEK1/2, G-protein beta/gamma, JNK(MAPK8-10), CTGF, c-Src, Elk-1, SDF-1, CXCR4, IL-2, PREX1 |
| 19 | Immune response_TCR alpha/beta signaling pathway | 5.35E-09 | STIM1, MHC class II, Calcineurin A (catalytic), STK39, CD45, Slp76, TRAF3, c-Fos, TRAF2, GLK(MAP4K3), ERK1/2, CABIN1, c-Jun/c-Fos, E2N(UBC13), Csk, Pannexin-1, Caspase-8, LAT, TRPV1, 14-3-3 theta, CaMK IV, IP3 receptor, CARD11, I-kB, MEF2D, AP-1, PKC-theta, Calmodulin, PKC-alpha, ZAP70, CD3, MALT1, PLC-gamma 1, ITK, P2X1, CD4, TAB1, MEK1/2, WASP, Fyn, Bcl-10, AKT(PKB), NF-kB p50/p65, UEV1A, Calcineurin B (regulatory), NF-AT2(NFATC1), IL-2, WaspIP |
| 20 | Apoptosis and survival_Regulation of apoptosis by mitochondrial proteins | 6.13E-09 | NIX, PLSCR3, Calcineurin A (catalytic), NOR1, IFI27, MPTP complex, PKC-delta, Fis1, Cathepsin H, 14-3-3 zeta/delta, ERK1/2, MAP1, BOK, BFL1, Pin1, PP2C, PP2A catalytic, PARL, VDAC 2, Bik, Cytochrome c, Caspase-8, MFF, OPA1, BMF, JSAP1, RAD9, NIP3, GZMH, Bax, OMA1, NUR77, SOD1, Kv1.3, SLC25A3, PP1-cat alpha, Beclin 1, Mitofusin 1, Bcl-XL, Cyclin A, VDAC 1, JNK(MAPK8-10), JNK2(MAPK9), MIDUO, Caveolin-1, Calpain 1(mu), DNM1L (DRP1), Mcl-1, Calcineurin B (regulatory), Smac/Diablo, Granzyme K |
| 21 | Cell adhesion_Tight junctions | 6.26E-09 | RhoA, Rich1, JAM2, Cingulin, MUPP1, EPB41, MPP5, F-Actin, ARP3, ACTR3, Myosin II, Claudin-1, Actin, DNMBP(TUBA), ROCK, Actomyosin, Myosin VIIA, PDZ-RhoGEF, Tubulin alpha, p114-RhoGEF, MRLC, CGNL1, Occludin, CDC42, Actin cytoskeletal, Angiomotin (AMOT), PARD6, Tubulin (in microtubules) |
| 22 | Immune response_Antigen presentation by MHC class II | 6.66E-09 | MHC class II alpha chain, RhoA, Dectin-1, MHC class II, PI3K cat class IA, AKT1, HSC70, LLIR, c-Cbl, PKC-delta, CLEC9A, MYO1E, CLEC10A, ERK1/2, Fc alpha receptor, HSP90 alpha, CD79 complex, PLEKHM2, PSD4, ARL14, Dynamin-2, RhoB, MAP1LC3A, Syk, 14-3-3 beta/alpha, Dynamin-1, ORP1, Rab-7, ARHGEF2, CD74, Dynein 1, cytoplasmic, heavy chain, PKC, Alpha-centractin, CD79A, SPPL2a, RILP (Rab interacting lysosomal protein), Gie1, Kinesin heavy chain, PKC-alpha, CD79B, CD4, Beclin 1, Fc gamma RII beta, CDC42, JNK(MAPK8-10), Clathrin, TRIF (TICAM1), HSP90, CD23, Kinesin light chain, MHC class II beta chain, R-Ras, Dynein 1, cytoplasmic, intermediate chains, LRP1, Tubulin (in microtubules) |
| 23 | Development_Negative regulation of WNT/Beta-catenin signaling in the nucleus | 6.77E-09 | KDM2, LRP5/LRP6, AKT1, TRRAP, Calcineurin A (catalytic), RUNX3, c-Cbl, HBP1, Oct-3/4, PKC-delta, Tcf(Lef), CDX2, Alpha-1 catenin, TCF7L2 (TCF4), 14-3-3, Jade-1, Casein kinase I epsilon, Beta-catenin, BCL9/B9L, PC1-CTT, CHD8, TLE, SENP2, Dsh, Menin, NF-AT5, RNF43, GSK3 beta, Nephrocystin-4, HDAC2, WNT, eNOS, HIC1, LATS2, P15RS, CHIBBY, RANBP3, CtBP, TBLR1, HIC5, Axin, SOX9, CDX1, Frizzled, Histone H1 |
| 24 | Signal transduction_Angiotensin II signaling via Beta-arrestin | 8.88E-09 | RhoA, GRAF2, GRK6, EGFR, SET, eIF4E, ERK1/2, 14-3-3, CACNA1C, PP2A catalytic, MNK1, Angiotensin II, Casein kinase II, alpha' chain (CSNK2A2), p90Rsk, STAR, ROCK, GSK3 beta, AP-2 alpha subunits, GRK5, PLC-gamma 1, MRLC, Beta-adaptin 2, Bcl-XL, TRPV4, DGK, Clathrin, AKT(PKB), MYLK1, c-Src, Clathrin heavy chain, SSH1L, MLCK, p23 co-chaperone |
| 25 | Immune response_B cell antigen receptor (BCR) pathway | 9.37E-09 | STIM1, RelA (p65 NF-kB subunit), LRRK1, Calcineurin A (catalytic), GRB2, c-Fos, NF-kB1 (p50), ICAM1, ETS1, Lyn, PIP5KIII, MEK3(MAP2K3), ERK1/2, EGR1, CD79 complex, PP2A catalytic, BCAP, PIP5KI, CD19, Btk, PI3K cat class IA (p110-delta), IP3 receptor, CARD11, Syk, HPK1(MAP4K1), Shc, GSK3 beta, Rb protein, Calmodulin, CD79A, CalDAG-GEFIII, FKHR, MALT1, PLC-gamma, CD79B, MEK2(MAP2K2), GSK3 alpha/beta, Bcl-XL, MEK1/2, CDC42, WASP, Actin cytoskeletal, Bcl-10, NF-kB, AKT(PKB), DAPP1, NF-kB p50/p65, Calcineurin B (regulatory), PLC-gamma 2, Elk-1, NF-AT2(NFATC1), MEKK4(MAP3K4) |
| 26 | Chemotaxis_SDF-1/ CXCR4-induced chemotaxis of immune cells | 1.19E-08 | BETA-PIX, RhoA, Pyk2(FAK2), PI3K cat class IA, CD45, CALDAG-GEFI, ICAM1, G-protein alpha-i family, F-Actin cytoskeleton, PAK2, ERK1/2, PKA-reg (cAMP-dependent), Csk, LIMK1, Btk, G-protein alpha-13, Talin, RAP-1A, p130CAS, Shc, PIPKI gamma, PAK, PLC-beta, Vinculin, PLEKHG2, ZAP70, CD3, CD3 zeta, ITK, RASSF5, MEK1/2, G-protein beta/gamma, CDC42, WASP, Fyn, AKT(PKB), SFK, TSAD, PLC-gamma 2, SDF-1, CXCR4 |
| 27 | Cell adhesion_Histamine H1 receptor signaling in the interruption of cell barrier integrity | 1.3E-08 | RhoA, VE-cadherin, Pyk2(FAK2), E-cadherin, PKC-delta, Myosin II, Beta-catenin, Alpha-actinin, CPI-17, G-protein alpha-q/11, LIMK1, Alpha-catenin, IP3 receptor, Talin, p130CAS, ROCK, Calmodulin, PLC-beta, Vinculin, PKC-alpha, MRLC, Occludin, G-protein beta/gamma, Actin cytoskeletal, LIMK2, MELC, c-Src, MLCK |
| 28 | Immune response_Function of MEF2 in T lymphocytes | 1.33E-08 | HDAC9, ERK5 (MAPK7), Calcineurin A (catalytic), c-Jun, MEF2C, 14-3-3, CaMK I, CACNA1C, CABIN1, HDAC4, HDAC7, MEF2, HDAC5, LAT, NCOA2 (GRIP1/TIF2), CaMK IV, IP3 receptor, MEF2D, CARM1, Calmodulin, NUR77, HDAC2, ZAP70, CD3, MAP3K3, PCAF, PLC-gamma 1, CaMKK, Calcineurin A (beta), IL-2 |
| 29 | WNT signaling in gastric cancer | 1.99E-08 | CD44, WNT9A, Tcf(Lef), beta-TrCP, TCF7L2 (TCF4), Beta-catenin, WNT10A, Dsh, Casein kinase I alpha, WNT1, Ubiquitin, WNT2B, GSK3 beta, SIAH1, WNT, WNT 8A, WNT10B, Skp2/TrCP/FBXW, Cyclin D1, SFRP1, Axin, FBXW11, Frizzled, TNF-alpha |
| 30 | G-protein signaling_Regulation of RAC1 activity | 1.99E-08 | ARCGAP22, SRGAP2(WRP), Slp76, ABR, BCR, ACK1, ARHGAP9, ARHGAP12, CD19, VIL2 (ezrin), ARHGEF4, ARHGAP24, ARHGEF2, GGTase-I, ZAP70, CD3, Rap1GDS1, DEF6, Caspase-3, p200RhoGAP, RacGAP1, Caspase-1, RalBP1, ECT2 |
